# Targeted inhibition of the CREB1-CtIP axis enhances the efficacy of abiraterone combined with radiotherapy in prostate cancer

**DOI:** 10.1038/s41419-026-08633-0

**Published:** 2026-03-30

**Authors:** Xu Han, Liang Song, Yuankang Feng, Zihao Wang, Lina Wang, Ruoyang Liu, Yu Liu, Ningyang Li, Saiyu Ma, Fubo Lu, Jinjian Yang, Zhenlin Huang, Zhankui Jia

**Affiliations:** 1 Department of Urology, The First Affiliated Hospital of Zhengzhou University, Zhengzhou, China; 2 Department of Urology, Tianjin Institute of Urology, The Second Hospital of Tianjin Medical University, Tianjin, China; 3 Department of General Medicine, Zhengzhou Central Hospital Affiliated to Zhengzhou University, Zhengzhou, China; 4 Nanozyme Medical Center, School of Basic Medical Sciences, Zhengzhou University, Zhengzhou, China

**Keywords:** Prostate cancer, Prostate cancer

## Abstract

In the treatment of locally advanced prostate cancer (PCa), abiraterone acetate (AA) serves both as a commonly used therapeutic agent and a radiosensitizer when combined with radiation therapy (RT). However, this combination therapy is not effective for all patients, and prolonged treatment may lead to decreased therapeutic sensitivity. Our study found that the combination of abiraterone (Abi, the active component of abiraterone acetate in vivo) and RT increases the expression of CtBP-interacting protein (CtIP) in prostate cancer cells, and elevated CtIP levels in PCa are associated with poor prognosis. CtIP has been demonstrated to be a key protein in the homologous recombination repair (HR) pathway of DNA damage repair (DDR). Furthermore, we observed that both Abi and RT enhance the transcriptional activity of CREB1 via phosphorylation, thereby modulating CtIP expression. Additionally, during the transition from normal prostate cells to prostate cancer cells, DNA demethylases (TETs) reduce DNA methylation levels in the promoter region of CtIP, facilitating the binding of CREB1 to the CtIP promoter. Finally, our in vitro and in vivo experiments indicate that the CREB1 phosphorylation inhibitor 666-15 significantly enhances the therapeutic efficacy of Abi-RT combination therapy. In summary, our study reveals that inhibition of the CREB1-CtIP axis effectively improves the therapeutic outcomes of Abi-RT combination therapy, which may offer a novel clinical strategy for the treatment of prostate cancer.

## Introduction

Prostate cancer (PCa) is a common malignancy affecting male health. Due to its slow progression and absence of symptoms in the early stage, it is often overlooked by patients, leading to a high rate of late-stage diagnosis, which further increases treatment difficulty and mortality [1–3]. Androgen deprivation therapy (ADT) is an effective treatment for PCa [4], but monotherapy often yields suboptimal outcomes in advanced patients. In recent years, studies have proposed combining ADT with radiation therapy (RT), novel endocrine therapy, or chemotherapy to improve overall treatment efficacy [5].

Abiraterone acetate (AA), a CYP17A1 inhibitor, is a prodrug of abiraterone (Abi) that exerts pharmacological effects only after hydrolysis of the acetate group by hepatic esterases and other enzymes in vivo to form the active metabolite Abi. It is widely used as an ADT agent for PCa treatment [6]. Radiotherapy is also a core curative modality for PCa [7], applicable to all disease stages and particularly effective for early localized or locally advanced cases [8, 9]. It primarily induces DNA damage in cancer cells through high-energy ionizing radiation (IR), impairing their proliferative capacity and leading to cell death during mitosis [10, 11]. Recent studies have shown that the androgen receptor (AR) can regulate the expression of genes involved in DNA damage response (DDR) [12, 13]. DDR encompasses multiple repair pathways, including direct repair and double-strand break repair such as homologous recombination (HR) and non-homologous end joining (NHEJ) [14]. Abi inhibits AR activity by suppressing androgen biosynthesis, which forms the theoretical basis for its combination with RT. Despite recommendations in guidelines worldwide for AA-RT combination therapy to improve the prognosis of advanced PCa patients [15–18], numerous clinical trials have failed to demonstrate a significant improvement in overall survival [19–23]. Therefore, elucidating the underlying mechanisms and identifying more effective radiosensitizers hold important clinical significance.

CREB1 is a transcription factor with a molecular weight of 43 kDa. Its transcriptional activity is activated upon phosphorylation at the Ser133 site within its kinase-inducible domain [24]. Kinases known to phosphorylate CREB1 include ATM, PKA, PKB, MAPK, and p90 ribosomal S6 kinase (p90RSK) [25]. CREB1 regulates various biological processes such as cell proliferation, differentiation, and neuroadaptive responses [26]. Immunohistochemical analysis of primary and bone metastatic prostate cancer tissues showed no detectable phosphorylated CREB1 (pCREB1) in normal benign prostate glands, while all examined poorly differentiated prostate cancer and bone metastasis samples showed positive pCREB1 staining [27]. pCREB1 levels positively correlated with tumor differentiation degree and metastatic range, suggesting a key role in tumor progression and metastasis. In LNCaP prostate cancer cells, CREB1 activation is associated with IR and cyclic adenosine monophosphate (cAMP)-induced neuroendocrine differentiation [28]. Recent studies have also found an association between CREB1 phosphorylation and resistance to AA [29]. However, no studies have yet explored the potential relationship between CtIP and CREB1.

CtIP, a critical protein in DDR [30], has been extensively reported in previous studies. Studies have shown that CtIP plays a central role in the selection of the HR pathway [31, 32], making CtIP-targeted therapy a potential strategy to enhance radiosensitivity. During our investigation into the clinical significance and experimental validation of Abi combined with RT for PCa treatment, we first discovered that CtIP expression is regulated by Abi and RT independently of AR. Subsequent studies identified CREB1 as a transcriptional regulator of CtIP. Mechanistic studies demonstrated that Abi and RT enhance CREB1 transcriptional activity through phosphorylation, thereby upregulating CtIP expression via transcriptional regulation. Notably, we found that during the malignant transformation of normal prostate cells to cancer cells, TET family enzymes induce a reduction in DNA methylation in the CtIP promoter region, creating a more favorable environment for CREB1 transcriptional binding. These findings provide a theoretical basis for targeting the CREB1-CtIP axis to enhance PCa radiosensitivity and propose a novel therapeutic strategy to improve clinical outcomes and optimize the efficacy of molecular targeted therapy.

## Materials and methods

### Data mining

We performed gene localization and transcription factor prediction for CtIP using the following resources: NCBI Gene (https://www.ncbi.nlm.nih.gov/gene/) database [33], Human Transcription Factor (Human TFDB; http://bioinfo.life.hust.edu.cn/HumanTFDB/) Database [34], The Cancer Genome Atlas (TCGA; https://portal.gdc.cancer.gov/) [35], and Cistrome DB (https://cistrome.org/) [36, 37]. Data visualization was conducted through the UCSC Genome Browser (https://genome.ucsc.edu/) [38], Gene Expression Profiling Interactive Analysis (GEPIA; http://gepia.cancer-pku.cn/) [39], and Integrative Genomics Viewer (IGV).

Analysis of 25 ChIP-seq datasets from the Gene Expression Omnibus (GEO; accession numbers: GEM759657, GEM759658, GEM2219855, GEM1527843, GEM1873041, GEM1873042, GEM1873046, GEM1862989, GEM1862991, GEM1527826, GEM503906, GEM2931699, GEM2931700, GEM2495657, GEM2495658, GEM2534513, GEM2527442, GEM1940197, GEM803531, GSM4387106, GSM897576, GSM897577, GSM952455, GSM952456, GSM952457; https://www.ncbi.nlm.nih.gov/geo) [33] was performed with subsequent visualization using UCSC and IGV.

Gene enrichment analysis for CtIP and CREB1 was conducted through Gene Set Enrichment Analysis (GSEA; https://www.gsea-msigdb.org/gsea/index.jsp) [40], Gene Ontology (GO; https://geneontology.org/) [41], Enrichr (http://amp.pharm.mssm.edu/Enrichr) [42–44], WikiPathways (https://www.wikipathways.org/) [45], and TCGA. CtIP expression in prostate cancer cell lines was investigated using TCGA, with prognostic correlations analyzed by TCGA, GEPIA and validated through the Human Protein Atlas (HPA; https://www.proteinatlas.org/) [46].

DNA methylation analysis of the CtIP promoter region, along with methylation assessment and motif prediction at CREB1 binding sites proximal to CtIP, was performed using the following resources: TCGA, UCSC, MethMotif (https://methmotif.org/) [47], JASPAR (https://jaspar.elixir.no/) [48], and the University of Alabama at Birmingham Cancer Data Analysis Portal (UALCAN; https://ualcan.path.uab.edu/index.html) [49, 50]. Finally, synergistic effects of drug combinations were evaluated using SynergyFinder 3.0 (https://synergyfinder.fimm.fi/) [51].

### Cell lines, cell culture and transfection

The RWPE-1, LNCaP, VCaP, 22Rv1, C4-2, DU145, PC-3, and 293 T cell lines were purchased from the Cell Bank of the Chinese Academy of Sciences (Shanghai, China). The C4-2B cell lines were purchased from SunnCell (Wuhan, China). RWPE-1 cells were cultured in Keratinocyte-SFM medium at 37 °C in 5% CO_2_. LNCaP, 22Rv1, C4-2, and C4-2B cells were cultured in RPMI-1640 medium supplemented with 10% FBS at 37 °C in 5% CO_2_. 293T, DU145 and VCaP cells were cultured in DMEM medium supplemented with 10% FBS at 37 °C in 5% CO_2_. PC-3 cells were cultured in Ham’s F-12 medium supplemented with 10% FBS at 37 °C in 5% CO_2_.

Abiraterone was gradually added to the culture medium of C4-2B cells at sequential concentrations of 1, 2, 5, 10, 20 and 30 μM, while the control group received an equal volume of DMSO. Following continuous culture for approximately 6 months, the cells were further cultured in medium containing 10 μM abiraterone for 3 months. Thus, abiraterone-resistant C4-2B cells (C4-2B-AbiR) were successfully established.

JetPRIME® in vitro DNA and shRNA transfection reagent (Polyplus-transfection, Strasbourg, France) was used for cell transfection, following the manufacturer’s protocol (shRNA sequence information in Supplementary Table S1). In addition, the CREB Dominant-Negative Vector Set (Takara Bio (valid) # 631925) was obtained for overexpressing wild-type CREB1 and mutated CREB. Plasmids of HA-CtIP were synthesized and purchased from the Public Protein/Plasmid Library in Jiangsu, China.

### Reverse transcription-quantitative polymerase chain reaction (RT-qPCR)

Total RNA was extracted using the Total RNA Extraction Kit (Boxbio, Beijing, China). Subsequently, cDNA was synthesized using the NovoScript® Plus All-in-one 1st Strand cDNA Synthesis SuperMix (Novoprotein, Shanghai, China). The gene transcripts of interest were quantified using the QuantStudio Three Real-Time PCR System (Thermo Fisher) using the NovoStart® SYBR qPCR SuperMix Plus (Novoprotein), with ACTB as an internal control. Relative gene expression was quantified using the 2^–ΔΔCt^ method. The Ct values from technical replicates were averaged, and the ΔCt for each biological replicate was calculated by subtracting the Ct value of the reference gene from that of the target gene. The ΔΔCt was then derived by subtracting the average ΔCt of the control group from the ΔCt of each experimental sample. The final relative expression is reported as 2^–ΔΔCt. The primer sequence used during our quantitative real-time polymerase chain reaction (qRT-PCR) test is shown in Supplementary Table S2.

### Chromatin immunoprecipitation followed by qPCR (ChIP-qPCR)

ChIP-qPCR was performed using the SimpleChIP® Enzymatic Chromatin IP Kit (Magnet Beads, #9003; Cell Signaling Technology). Briefly, 5 × 10⁶ cells were cross-linked with 1% formaldehyde for 10 min at room temperature, followed by quenching with 2 mL of 10× glycine (5 min). Cells were washed twice with ice-cold phosphate-buffered saline (PBS), resuspended in lysis Buffer A containing 1 mM DTT and protease inhibitor cocktail, and incubated on ice for 10 min. Nuclei were pelleted by centrifugation and digested in Buffer B with 0.5 U/μL micrococcal nuclease and 1 mM DTT at 37 °C for 20 min. Digestion was terminated by adding 50 mM EDTA. Chromatin was fragmented using an Xinzhi sonicator equipped with a 1/8-inch probe (3 × 20-s pulses), and 2% of the sonicated lysate was retained as input DNA. Sonicated chromatin was immunoprecipitated overnight at 4 °C with anti-pCREB1 (#9198; Cell Signaling Technology) or IgG isotype control (#3900; Cell Signaling Technology). Protein G Magnetic Beads were added for 2 h at 4 °C, followed by magnetic separation and sequential washes. DNA-protein crosslinks were reversed by incubation with Proteinase K at 65 °C for 2 h. DNA was purified using the kit’s spin columns and quantified by qPCR to assess target enrichment. The primer sequence used is shown in Supplementary Table S2.

### RNA sequencing (RNA-seq)

Total RNA was isolated from samples using TRIzol reagent (Invitrogen, Carlsbad, CA, USA) according to the manufacturer’s protocol. RNA quality was assessed with a Bioanalyzer 2100 system (Agilent, Santa Clara, CA, USA) and RNA 6000 Nano LabChip Kit, ensuring RNA Integrity Numbers > 7.0. Polyadenylated [poly(A)+] mRNA was enriched from 10 µg of total RNA using oligo(dT)-conjugated magnetic beads (Invitrogen, Waltham, MA, USA). Purified RNA was fragmented into ~200–300 nucleotide segments via incubation with divalent cations at 94 °C for 5 min. Fragmented RNA was reverse-transcribed into cDNA using the Illumina TruSeq Stranded mRNA Library Prep Kit (Illumina, San Diego, CA, USA), and libraries were size-selected to generate inserts averaging 300 bp (±50 bp) for paired-end sequencing. Final libraries were sequenced on an Illumina HiSeq 4000 platform (lc-bio, Hangzhou, China) following standard protocols.

### Immunoprecipitation

The treated cells were lysed in ice-cold IP lysis buffer (Servicebio, G2038) supplemented with a complete protease and phosphatase inhibitor cocktail. For pre-clearing, Protein A/G magnetic beads (HY-K0202, MCE) were incubated with the protein lysate. Subsequently, the corresponding antibodies were introduced and incubated at 4 °C for 2 h. Following this, Protein A/G magnetic beads were added, and the mixture was incubated overnight at 4 °C. The beads were then boiled in Sodium Dodecyl Sulfate (SDS) loading buffer for subsequent western blot analysis. Primary antibodies used for immunoprecipitation included those against BRD4, CREM, SPI1, pan-phospho-serine/threonine, mouse IgG control, and rabbit IgG control. The corresponding antibody information is provided in Supplementary Table S4.

### Western blot (WB)

Total protein was extracted from cells using RIPA lysis buffer (Beyotime, Shanghai, China) supplemented with 1% PMSF (Solarbio, Beijing, China) and 1% phosphatase inhibitor cocktail A (Beyotime). Protein concentrations were quantified using a bicinchoninic acid (BCA) assay kit (Solarbio). Proteins were separated by SDS-Polyacrylamide Gel Electrophoresis (PAGE) (Epizyme, Shanghai, China) and subsequently transferred onto PVDF membranes. After blocking with QuickBlock™ Blocking Buffer (Beyotime) for 30 min at room temperature, membranes were incubated with primary antibodies overnight at 4 °C. Following three washes with TBST, membranes were incubated with fluorescent dye-conjugated secondary antibodies for 1 h at room temperature in the dark. Protein signals were visualized using an Odyssey CLx Infrared Imaging System (Gene Company Limited, Hong Kong, China). The band intensities were quantified using Image Studio software. The intensity of the target protein band was normalized to that of the internal control protein GAPDH for each sample to determine the relative protein levels. The normalized values of the experimental groups were then compared to the average normalized value of the control group to calculate the fold change. The corresponding antibody information is provided in Supplementary Table S4.

### cAMP and PKA activity assays

Intracellular cAMP concentrations were measured using the Direct Cyclic AMP ELISA Kit (ab133038, Abcam). Following cell lysis with 0.1 M HCl, lysates were incubated in anti-rabbit IgG-coated wells with alkaline phosphatase-conjugated cAMP antigen and specific antibody. Substrate development using pNpp preceded reaction termination, with yellow chromogen intensity quantified spectrophotometrically at 405 nm. All measurements underwent blank control normalization to ensure analytical accuracy.

The protein kinase A (PKA) enzymatic activity was quantified with the EIAPKA Colorimetric Activity Kit (Thermofisher) through the following procedure: Cellular lysates were mixed with ATP in microplate wells coated with a synthetic peptide specifically recognized by PKA, enabling kinase-mediated phosphorylation. Phosphorylated peptides were subsequently detected using a primary antibody targeting the modified substrate, followed by an HRP-coupled secondary antibody. 3,3',5,5'-Tetramethylbenzidine (TMB) substrate and stop solution were used, and the absorbance was measured at 450 nm. The protein concentration of the cell lysis was used as the reference.

### Cell counting kit-8 assay (CCK8)

Cellular proliferation was assessed by CCK-8 assay (Dojindo, Tokyo, Japan) with about 4 × 10³ cells seeded per well in 96-well plates. Following 24-h preconditioning under standard culture conditions (37 °C, 5% CO_2_), 10 μL CCK-8 reagent was introduced to each microculture unit. Post 4-h chromogenic reaction, optical density readings were obtained at 450 nm using a full-wavelength microplate reader (Perlong DNM-9602G), with three technical replicates performed per experimental condition.

### Colony formation assay

Cells were density-optimized based on growth kinetics and morphology, then seeded in 6-well plates overnight. Cultures were maintained under standard conditions (37 °C, 5% CO_2_) for 7–14 days until macroscopic clones (50–200 cells/clone) emerged. Cellular architectures were preserved through 4% paraformaldehyde fixation (30 min), followed by PBS washes to eliminate fixative residuals. Clone visualization was enhanced by 0.1% crystal violet staining (Beyotime, 15 min), with colony quantification performed using standardized counting protocols.

Following density optimization guided by growth kinetics and morphological characteristics, cells were equilibrated in 24-well plates for 12–16 h. Experimental groups received Dimethyl Sulfoxide (DMSO,vehicle control), 666-15, and JQ1 at varying concentrations. Cultured cells underwent synchronized termination at predefined intervals. Fixed samples were stained with crystal violet and microscopically analyzed.

### Bisulfite genomic sequence polymerase chain reaction (BSP)

Bisulfite sequencing (BSP) was conducted following established protocols. A 2100bp genomic region flanking the transcription start site was retrieved from NCBI (GRCh38/hg38). CpG island prediction within the promoter’s specific region was performed using UCSC Genome Browser (2013 Assembly). Bisulfite-converted genomic DNA underwent PCR amplification with primers designed via MethPrimer (v2.0, www.urogene.org). Sequencing reactions were executed on an ABI3730XL platform, with methylation patterns analyzed using BiQ Analyzer software.

### Methylation-specific polymerase chain reaction (MSP)

Genomic DNA was isolated from prostate cancer cell lines and normal prostate epithelial cells (RWPE-1) using a commercial extraction kit (ELK Biotechnology, EP007), adhering to manufacturer specifications. Bisulfite conversion was performed on purified DNA with a Methylation-Gold Kit. PCR amplification employed MethPrimer-designed primers (Supplementary Table S3) targeting specific CpG loci. Amplified products (10 μl) mixed with loading dye were electrophoresed on 2% agarose gels (100 V, 30 min). DNA bands were visualized under UV transillumination and documented using a gel imaging system.

### Comet assay

Alkaline comet assays were performed with the Trevigen comet assay kit (C2041M, Beyotime, Shanghai, China) using the manufacturer’s instructions. Briefly, cell suspensions were embedded in LM (low-melting) Agarose and deposited on Comet Assay Slide (FSL061-5pcs, Beyotime, Shanghai, China). Slides were incubated for 1–2 h at 4 °C in lysis solution, followed by immersing slides in freshly prepared alkaline unwinding solution (pH > 13) for 20–60 min at room temperature in the dark. Electrophoresis was carried out for 30 min at 21 V in electrophoresis solution (pH > 13). Slides were then stained (C4-2B, Propidium Iodide; PC-3, DAPI). Finally, samples were mounted with antifade medium and imaged using a BX53F2 microscope (Olympus Life Science, Tokyo, Japan). Tail DNA content was analyzed with Comet score 1.5 software. DNA strand breakage was expressed as “comet tail moment”. The tail moment was measured for a minimum of 50 cells per sample, and average damage from 3 independent experiments was calculated.

### Immunofluorescence staining and laser scanning confocal microscopy

Cells were seeded in 35-mm confocal dishes (BS-20-GJM; Biosharp, Beijing, China) for 24 h. After treatment, cells were washed three times with PBS. DNA damage was detected using a commercial kit (DNA Damage Detection Kit, #C2037S; Beyotime, Shanghai, China). Briefly, cells were fixed with 4% paraformaldehyde for 15 min at room temperature. Following PBS washes, samples were blocked with immunostaining blocking buffer for 10–20 min at room temperature. Cells were then incubated with a γ-H2AX mouse monoclonal antibody (1:200 dilution) for 1 h at room temperature or overnight at 4 °C. After washing, Alexa Fluor 488-conjugated anti-mouse IgG secondary antibody (1:500) was applied for 1 h at room temperature. Nuclei were counterstained with 4′,6-diamidino-2-phenylindole (DAPI) for 5 min. Finally, samples were mounted with antifade medium and imaged using a LSM 880 laser scanning confocal microscope (Carl Zeiss, Germany).

### HR repair reporter

The HR repair reporter assay was conducted as described previously [52, 53]. In brief, cells were transfected with pLCN double‑strand break (DSB) Repair Reporter (Addgene plasmid #98895), pCAGGS DRR mCherry Donor EF1a BFP (Addgene plasmid #98896), and pCBASceI plasmid (Addgene plasmid #26477) for 72 h before fluorescence-activated cell sorting analysis (these plasmids were gifts from Jan Karlseder). BFP-positive cells were gated for mCherry analysis.

### Synergy calculations

Synergy data were analyzed with online software SynergyFinder 3.0. ZIP synergy scores over 10 were considered to be synergistic, ZIP synergy scores between 0 and 10 were considered to be additive, and ZIP synergy scores below 0 were considered antagonistic.

### In vivo tumor xenograft model

1. For the subcutaneous xenograft male BALB/c nude model, 1 × 10^6^ C4-2B cells with or without CtIP stable overexpression were suspended in the Matrigel (Becton, Dickinson and Company, Franklin Lakes, NJ, USA) in a 1:1 ratio, extracted with a 1-mL syringe, and injected into the subcutaneous at a 45° angle of male BALB/c nude mice to establish tumors. After 7 days, mice were randomized into different groups. For combination therapy experiments, mice were randomized into different treatment groups (5 mice per group), including vehicle (DMSO), Abiraterone (60 mg/kg, Intraperitoneal injection, twice a week), and IR (8 Gy) on the 7th day (Abi was administered 4 h prior to IR.). After 35 days, the mice were sacrificed, and the tumor masses were removed and photographed. Tumor volume and body weight were measured every 7 days, with tumor volume calculated as follows: (short diameter)^2^ × (long diameter)/2.
2. To evaluate the effect of the CREB1 phosphorylation inhibitor 666-15, 1 × 10^6^ C4-2B cells were injected into the flanks of male BALB/c nude mice to construct a subcutaneous xenograft tumor model. After 7 days, mice were randomized into different treatments, including DMSO, Abi (60 mg/kg, Intraperitoneal injection, twice a week), 666-15 (10 mg/kg, Intraperitoneal injection, 5 days a week), and IR (8Gy) on 7th day (Abi and 666-15 was administered 4 h prior to IR.). After 35 days, the mice were sacrificed, and the tumor masses were removed and photographed. Tumor volume and body weight were measured every 7 days, with tumor volume calculated as follows: (short diameter)^2^ × (long diameter)/2.

In all cases, male BALB/c nude mice (age 3–5 weeks) were purchased from the Sipeifu Company (Beijing, China). Mice were anesthetized by intraperitoneal injection of tribromoethyl alcohol (HY-B1372, MedChemExpress, Monmouth Junction, NJ, USA) before irradiation. Tumor xenografts were selectively irradiated using a PXI X-RAD320 biological irradiator (Precision X-Ray Inc., North Branford, CT, USA), with lead collimators (5 mm thickness) shielding adjacent normal tissues. The mice were euthanized by CO_2_ asphyxia and subsequent cervical dislocation. All experimental procedures were approved by the Institutional Animal Care Committee and the local animal ethics committee. All methods were performed in accordance with relevant guidelines and regulations and in accordance with ARRIVE guidelines (Approval number: 2024-KY-1207-001).

### In vivo toxicity of combined therapy

In the toxicity study, subcutaneous xenograft tumors were established by injecting 1 × 10⁶ C4‑2B cells into the flank of male BALB/c nude mice (3–5 weeks old). Seven days later, the mice were randomly assigned to four groups (*n* = 5 per group). One group served as the control and received intravenous injections of DMSO. The other three groups underwent the same treatments, including Abi (60 mg/kg, intraperitoneal injection, twice weekly), 666-15 (10 mg/kg, intraperitoneal injection, 5 days per week), and local irradiation with 8Gy at the site of subcutaneous xenograft tumor establishment (Abi and 666-15 were administered 4 h prior to IR.). Mice were euthanized at 1 h, 1 day, and 28 days following the respective combination treatments. Major organs, including the heart, liver, spleen, lungs, and kidneys, were harvested for histopathological examination. Blood samples were collected from euthanized mice, and serum biomarkers of hepatic and renal function were measured using an automated biochemical analyzer (Indiko, Thermo Fisher Scientific). All methods were performed in accordance with relevant guidelines and regulations and in accordance with ARRIVE guidelines (Approval number: 2024-KY-1207-001).

### Statistical analysis

All data were analyzed using GraphPad Prism 9.0 (LaJolla, USA) and expressed as the mean ± SD. Student’s *t*-test or ANOVA was used to analyze the groups unless otherwise indicated. *P* values < 0.05 indicated statistical significance.

## Results

### CtIP is involved in the administration of the abiraterone and RT combination therapy

**Fig. 1:**
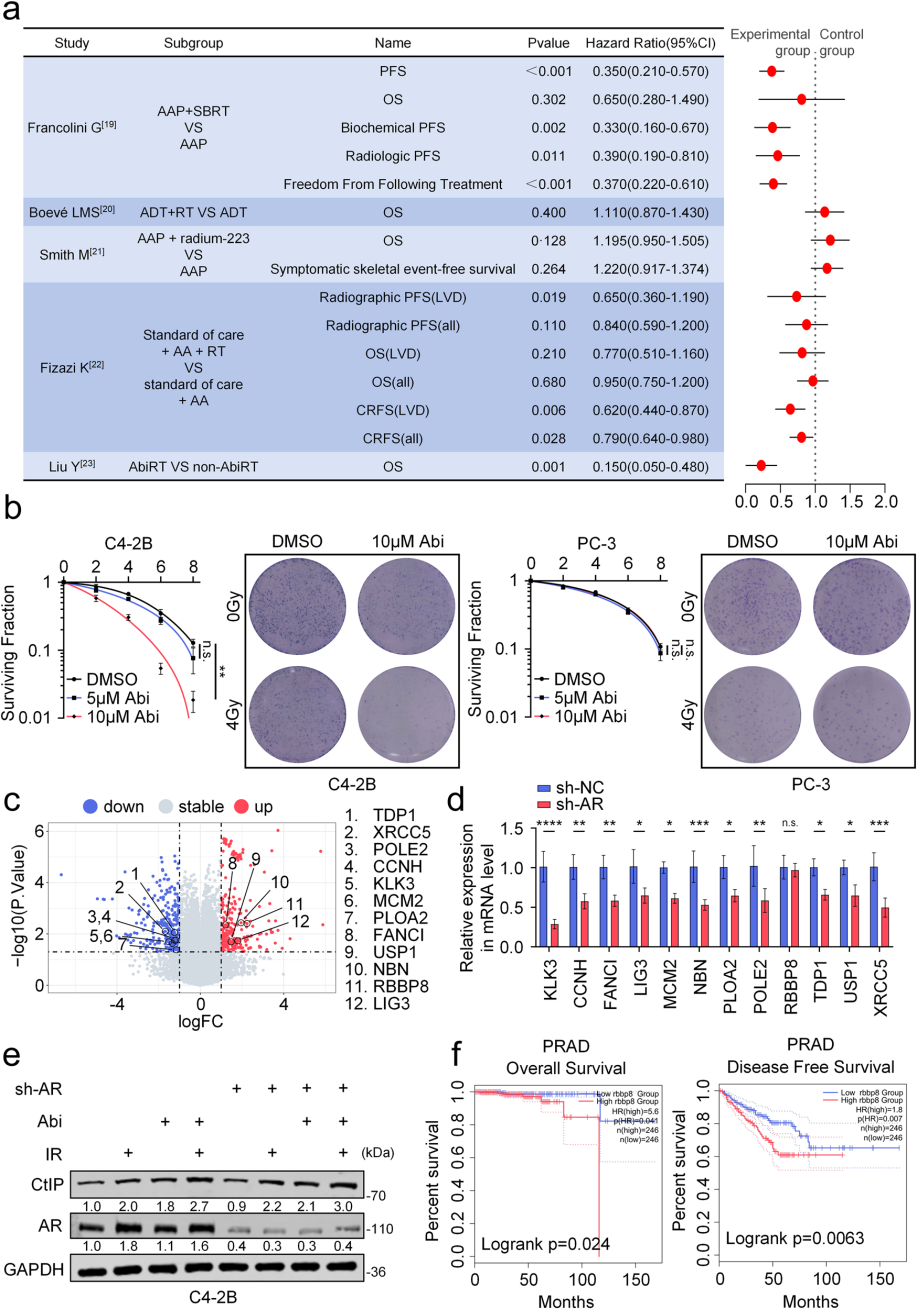
CtIP is involved in the administration of the abiraterone and RT combination therapy. **a** Multiple clinical trials investigating the combined efficacy of abiraterone acetate (AA) with radiotherapy, with relevant survival outcomes as primary endpoints. The left table summarizes the trial sources, study groups, and corresponding results, while the right forest plot illustrates hazard ratios (HR) with 95% confidence intervals (95% CI) for survival outcomes. **b** Clonogenic survival was reduced with the combined treatment of Abi (added 48 h prior to IR) combined with IR in C4-2B cells, only very minor effects in PC-3 cells. Representative images are shown in the right panels of each section. n.s., no significance; ^**^P < 0.01, unpaired *t*-test. **c** The sequencing analysis of C4-2B cells treated with either Abi (10 μM) or DMSO for 48 h is presented. Triplicate samples were analyzed per group. The corresponding volcano plot illustrates the up- and down-regulated genes following Abi treatment. **d** The expression of Genes related to DNA damage repair and other key genes (KLK3) in prostate cancer cells (C4-2B) after AR knockdown. n.s., no significance; ^*^P < 0.01, ^**^P < 0.01, ^***^P < 0.001, ^****^P < 0.0001, unpaired *t*-test. **e** Western blot analysis was performed to assess the expression of CtIP protein in C4-2B cells under control group and following 48 h of experimental treatment, including with or without AR knockdown, and monotherapy or combination therapy with Abi (10 μM, added 48 h prior to irradiation) and/or IR (2Gy). **f** Disease-free survival analysis and Overall survival for patients with high and low expression of CtIP from TCGA database. AAP abiraterone acetate and prednisone, SBRT stereotactic body radiation therapy, ADT androgen-deprivation therapy, AbiRT patients receiving cRT before abiraterone failure, non-AbiRT, patients who did not receive cRT before abiraterone failure; cRT cytoreductive radiotherapy.

In recent years, guidelines from multiple countries and regions have recommended the combination of AA and RT as a strategy to enhance treatment efficacy in advanced prostate cancer. We systematically analyzed multiple clinical trials evaluating the efficacy of AA combined with RT. Our synthesis of trial outcomes revealed that while combination therapy significantly improved progression-free survival (PFS), particularly in oligometastatic prostate cancer patients (HR < 1, *P* < 0.05), it failed to demonstrate a comparable benefit in overall survival (OS). These findings suggest that the AA-RT combination does not universally achieve theoretical therapeutic advantages, especially in cases of complex or polymetastatic prostate cancer (Fig. 1a) [19–23]. To investigate the impact of AA on radiotherapy, we selected AR-positive (C4-2B) and AR-negative (PC-3) cell lines representing metastatic prostate cancer. After 48 h of treatment with 10 μM abiraterone (Abi, the active component of abiraterone acetate in vivo), C4-2B cell growth was significantly inhibited, whereas PC-3 cells required 20 μM Abi for partial inhibition (Fig. S1a). To assess Abi’s effect on RT sensitivity, cells were treated with Abi (0, 5, 10 μM) for 48 h followed by ionizing radiation (IR; 0, 2, 4, 6, 8 Gy). Results showed that in C4-2B cells, only 10 μM Abi combined with IR significantly differed from monotherapy, but no similar effect was observed in PC-3 cells (Fig. 1b). RNA-seq analysis of C4-2B cells treated with 10 μM Abi for 48 h identified DNA damage repair (DDR) pathway genes (|log2FC| > 1, *P* < 0.05). Results revealed 6 downregulated genes (*TDP1, XRCC5, POLE2, CCNH, MCM2, PLOA2*) and 5 upregulated genes (*FANCI, USP1, NBN, RBBP8* (*CtIP*)*, LIG3*), with KLK3 as an AR downstream reference gene (Fig. 1c). Previous studies have demonstrated that AR regulates DDR-related genes [12, 13]. To verify this relationship, we knocked down AR in C4-2B cells (Fig. S1b, c) reduced the expression of all genes except CtIP (Fig. 1d). To investigate Abi-mediated regulation of CtIP at the protein level, we performed Western blot analysis. The results demonstrated that CtIP expression in C4-2B cells treated with Abi (10 μM, 48 h) exhibited a peak followed by progressive attenuation (Fig. S1d). Both Abi and IR increased CtIP expression in AR-positive C4-2B and AR-negative PC-3 cells, independent of AR regulation (Figs. 1e and S1e).

To investigate the role of CtIP in combination therapy, we hypothesized that elevated expression of CtIP compromises the radiosensitizing effect of Abi. CtIP overexpression in C4-2B cells (Fig. S1f, g) reduced γH2AX immunofluorescence and protein levels in Abi-IR therapy (Fig. S1h, i). Colony formation and CCK8 assays demonstrated enhanced RT efficacy with Abi, which was reversed by CtIP overexpression (Figs. S1j, k and S2a). In PC-3 cells, while Abi failed to demonstrate an effect in enhancing radiosensitivity, overexpression of CtIP similarly reduced sensitivity to radiotherapy (Figs. S1f–k and S2a). Cell-derived(C4-2B) xenograft experiments in BALB/c nude mice yielded consistent in vivo results (Fig. S2b-e). Collectively, our results show that increased levels of CtIP overcome the radio-sensitization mediated by Abi. TCGA-based DFS and OS analyzes revealed worse outcomes in CtIP-high prostate cancer patients. HPA database immunohistochemistry data further showed higher CtIP expression in high-grade versus low-grade prostate cancer (Figs. 1f and S2f), suggesting that CtIP levels negatively correlate with prognosis.

### CtIP promotes DDR via HR to influence radiosensitivity in prostate cancer cells

**Fig. 2:**
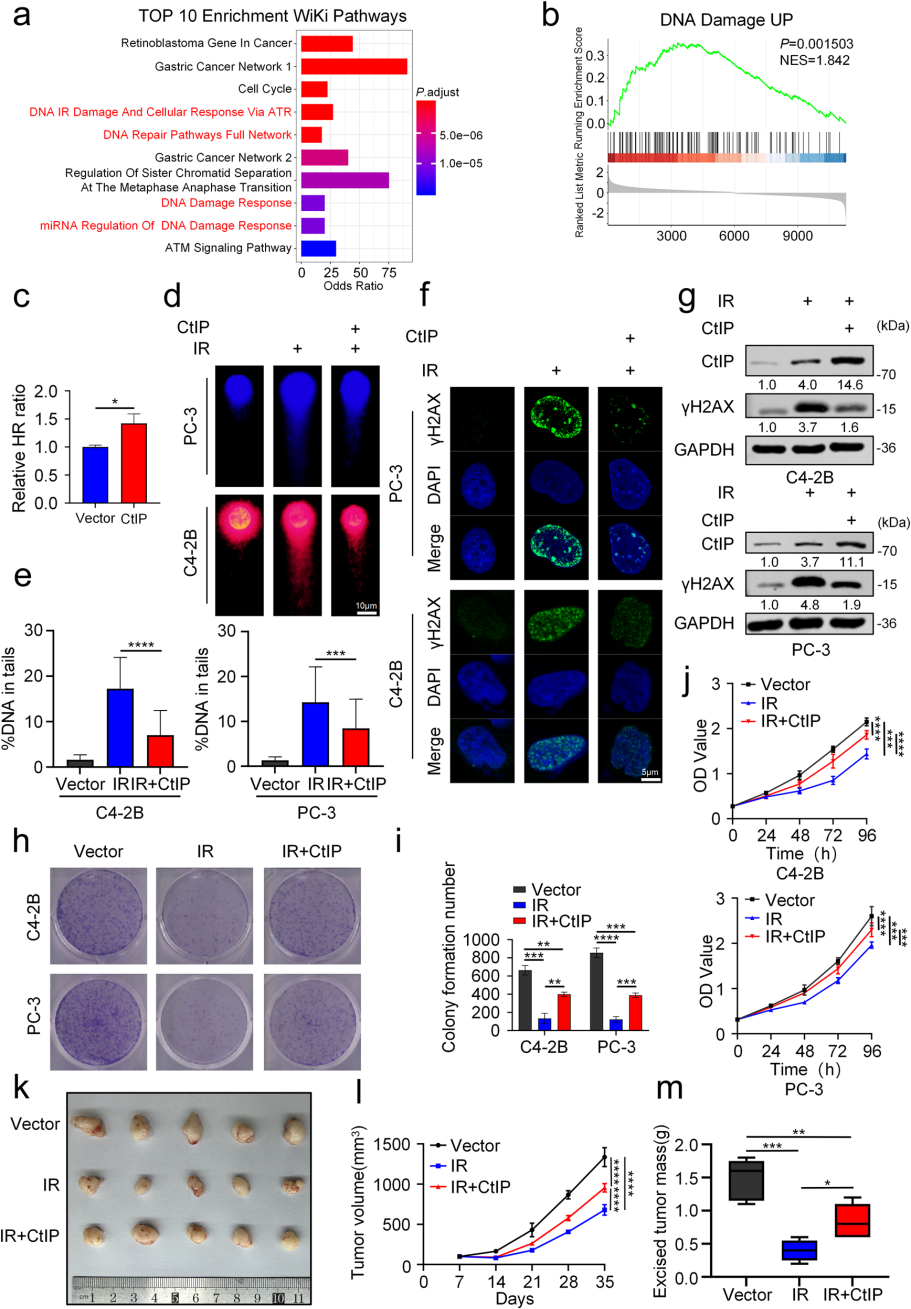
CtIP promotes DDR via HR to influence radiosensitivity in prostate cancer cells. **a** WikiPathways enrichment analysis was performed via the Enrichr database using a gene set comprising the top 100 genes exhibiting the highest co-expression with CtIP (identified by expanding its functionally associated gene set via the ARCHS4 RNA-seq gene-gene co-expression matrix). **b** Gene Set Enrichment Analysis (GSEA) enrichment analysis was conducted on differentially expressed genes identified from the TCGA prostate cancer (PRAD) cohort, which was stratified based on CtIP expression levels (high vs. low) and analyzed using the R software. **c** Homologous recombination repair efficiency was assessed by fluorescent reporter assay in control HeLa cells versus those following 48-h CtIP overexpression. The mCherry‑positive/BFP‑positive cell ratios were quantified (*n* = 3 biological replicates). ^*^P < 0.05, unpaired *t*-test. **d**, **e** Neutral comet assays evaluating DNA damage repair kinetics in C4-2B and PC-3 cells following a 48-h incubation after treatment. Treatment conditions included: control group; IR (4Gy); IR combined with CtIP overexpression (added 48 h prior to IR). DNA damage was quantified as % tail DNA (≥ 50 cells/group). Scale bars: 10 μm. ^***^P < 0.001, ^****^P < 0.0001, unpaired *t*-test. **f** Representative images of γH2AX(Ser139) foci in C4-2B and PC-3 cells following a 48-h incubation after treatment. Treatment conditions included: control group; IR (4Gy); IR combined with CtIP overexpression (added 48 h prior to IR). Scale bars: 5 μm. **g** Western blot analysis of indicated proteins in C4-2B and PC-3 cells following a 48-h incubation after treatment. Treatment conditions included: control group; IR (4Gy); IR combined with CtIP overexpression (added 48 h prior to IR). Representative images (**h**) and quantitative analysis (**i**) of colony formation assays in C4-2B and PC-3 cells following a 14-day incubation after treatment. Treatment conditions included: control group; IR (4Gy); IR combined with CtIP overexpression (added 48 h prior to IR). ^**^P < 0.01, ^***^P < 0.001, ^****^P < 0.0001, ANOVA. **j** CCK8 OD value (measured every 24 h for 96 h) and statistical analysis were assessed in C4-2B and PC-3 cells under three treatment conditions: control group; IR (4Gy); IR combined with CtIP overexpression (added 48 h prior to IR). ^***^P < 0.001, ^****^P < 0.0001, ANOVA. The representative image illustrates the C4-2B tumor xenograft model (**k**) in male BALB/c nude mice (*n* = 5), together with the statistical data of tumor volume (**l**) and weight (**m**) from different treatment groups. The groups consisted of: the control group; IR (8Gy) irradiation alone; and IR combined with CtIP overexpression. ^*^P < 0.05, ^**^P < 0.01, ^***^P < 0.001, ^****^P < 0.0001, ANOVA.

Previous studies have identified CtIP as a key protein in homologous recombination (HR) [54], while radiotherapy (RT) primarily kills cancer cells by inducing DNA double-strand breaks to inhibit proliferation [10]. To test this mechanism, we employed a dual bioinformatics approach. First, CtIP-associated gene sets (Supplementary Table S5) were generated using the ARCHS4 co-expression matrix on the Enrichr platform, and the top 100 correlated genes were analyzed via WikiPathways and GO enrichment. Second, GSEA was performed after stratifying samples from the TCGA prostate cancer cohort into high-expression and low-expression groups based on the average expression levels of CtIP. Collectively, these analyzes demonstrated CtIP’s participation in DDR pathways (Figs. 2a, b and S3a). Therefore, we aimed to investigate CtIP’s impact on radiosensitivity in prostate cancer.

Given CtIP’s role in DDR, we hypothesized that CtIP overexpression reduces RT sensitivity in Prostate Cancer Cells. To test this, we selected C4-2B and PC-3 cells (AR-positive and AR-negative bone metastasis-derived prostate cancer cell lines, respectively) with low and comparable baseline CtIP expression (Fig. S3b, c). Experimental results demonstrated that CtIP overexpression increased HR repair rates, confirming its critical role in HR (Fig. 2c). Comet assays revealed reduced IR-induced DNA damage in CtIP-overexpressing cells (Fig. 2d, e). Western blot (WB) and immunofluorescence analyzes showed significantly decreased γH2AX protein levels and fluorescence intensity in CtIP-overexpressing cells compared to IR-only groups (Fig. 2f, g). Colony formation and CCK8 assays further confirmed reduced radiosensitivity with CtIP overexpression (Fig. 2h–j).

To strengthen these findings, C4-2B cell-derived xenograft experiments in BALB/c nude mice yielded in vivo results consistent with in vitro trends (Figs. 2k–m and S3d). In summary, our findings are consistent with previous reports identifying CtIP as a key gene in the homologous recombination pathway [54]. Moreover, we have further validated in the prostate cancer cell lines C4-2B and PC-3 that overexpression of CtIP reduces the sensitivity of metastatic prostate cancer cells to IR.

### CREB1 transcriptionally regulates CtIP expression

**Fig. 3:**
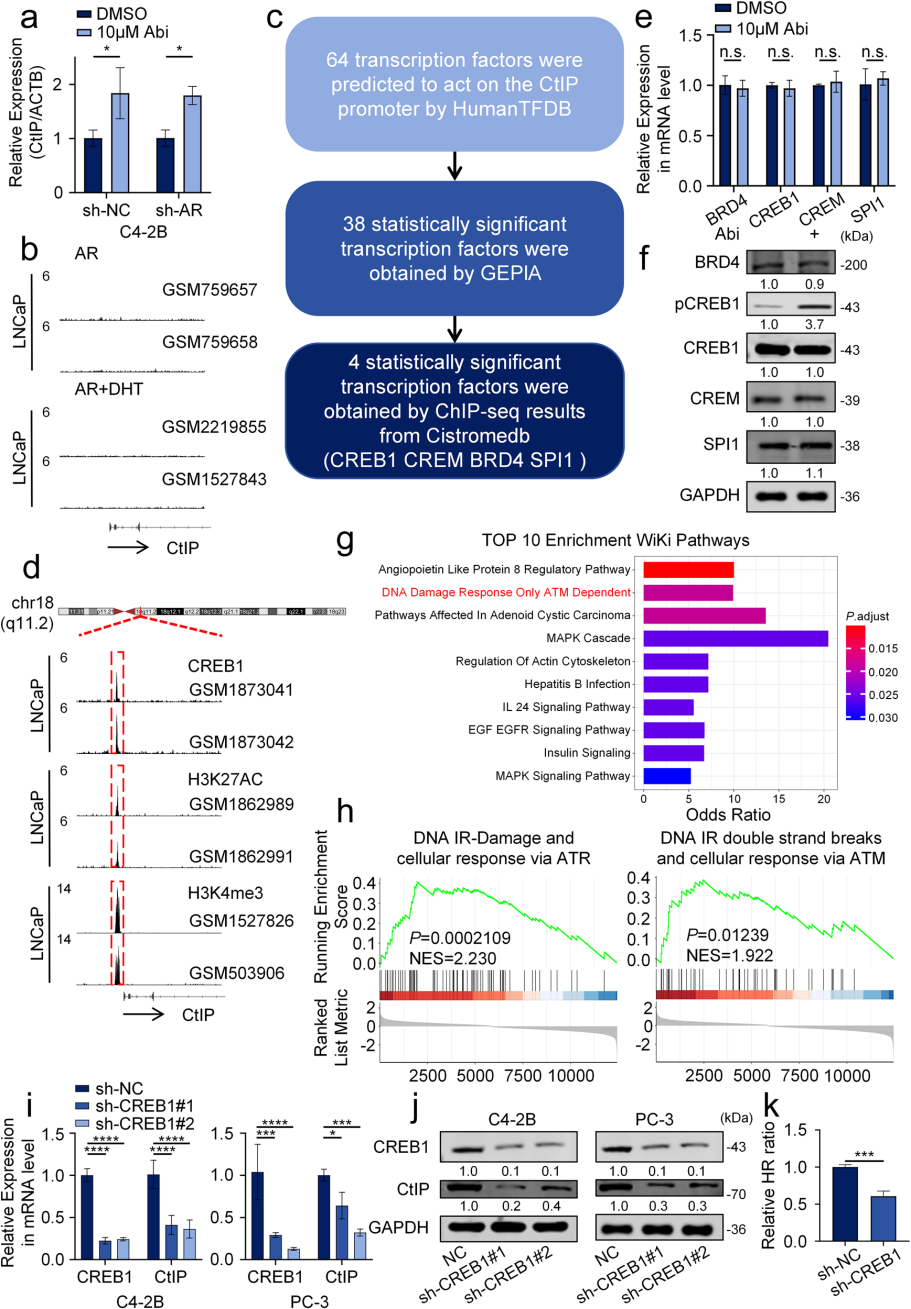
CREB1 transcriptionally regulates CtIP expression. **a** Western blot analysis of indicated proteins in C4-2B cells following a 48-h incubation after treatment. Treatment conditions included: control group; Abi (10 μM); AR knockdown; AR knockdown combined with Abi. ^*^P < 0.05, unpaired *t*-test. **b** The ChIP-seq results showed the binding of AR to the CtIP promoter region. **c** The process of screening transcription factors: CREB1, CREM, BRD4, SPI1. **d** The ChIP-seq results showed the binding of CREB1, H3K27ac and H3K4me3 to the CtIP promoter region (red dotted line area). **e** RT-qPCR analysis was performed to examine the expression levels of CREB1, CREM, BRD4, and SPI1 in C4-2B cells after treatment with Abi (10 μM) for 24 h. **f** Western blot analysis was performed to examine the expression levels of CREB1, phosphorylated CREB1 (pCREB1), CREM, BRD4, and SPI1 in C4-2B cells after treatment with Abi (10 μM) for 48 h. **g** WikiPathways enrichment analysis was performed via the Enrichr database using a gene set comprising the top 100 genes exhibiting the highest co-expression with CREB1 (identified by expanding its functionally associated gene set via the ARCHS4 RNA-seq gene-gene co-expression matrix). **h** Gene Set Enrichment Analysis (GSEA) enrichment analysis was conducted on differentially expressed genes identified from the TCGA prostate cancer (PRAD) cohort, which was stratified based on CREB1 expression levels (high vs. low) and analyzed using the R software. RT-qPCR (**i**) and Western blot (**j**) analysis showed the changes in CtIP expression with/without knockdown CREB1 in C4-2B and PC-3 cells. ^*^P < 0.05, ^***^P < 0.001, ^****^P < 0.0001, unpaired *t*-test. **k** HR repair reporter assay in Control and knockdown CREB1 HeLa cells. The population of mCherry+ cells in BFP+ cells was analyzed. Data from three biological replicates were presented. ^***^P < 0.001, unpaired *t*-test.

Previous studies have demonstrated that the androgen receptor (AR) transcriptionally regulates genes associated with DDR [12], thereby influencing the radiosensitivity of prostate cancer—a finding that our experimental results also corroborate (Fig. 1d). However, our research further revealed that AR does not regulate the expression of CtIP (Figs. 1e, 3a and S4a, b). Chromatin immunoprecipitation sequencing (ChIP-seq) data from the GEO database further confirmed the absence of AR binding peaks in the promoter region of CtIP (Fig. 3b). Notably, Abi treatment significantly increased the RNA expression level of CtIP (Fig. 3a), leading us to hypothesize that a transcription factor activated by Abi promotes CtIP expression. Through bioinformatic screening, we identified four candidate transcription factors: CREB1, CREM, BRD4, and SPI1 (Figs. 3c, d and S4c, d). However, Abi treatment did not alter the mRNA or protein levels of these factors (Fig. 3e, f). A review of relevant literature revealed that the activation of these transcription factors is associated with their phosphorylation [55–58]. Therefore, we examined changes in their phosphorylation status following Abi treatment by WB (or by immunoprecipitation assay when specific phospho-antibodies were unavailable). The results indicated that CREB1 phosphorylation was the most significantly enhanced (Figs. 3f and S4e). Concurrently, ChIP-seq analysis from the GEO database showed overlapping binding peaks of CREB1, pCREB1, H3K27ac, and H3K4me3 in the CtIP promoter region (Figs. 3d and S4d), suggesting transcriptional activity in this region. Thus, CREB1 was identified as the transcription factor responsible for mediating Abi-induced regulation of CtIP. Similarly, bioinformatic enrichment analysis of CREB1 (using the same methodology as applied to CtIP) indicated that CREB1, as a transcription factor, is involved in the DDR process (Figs. 3g, h and S5a); these results are consistent with prior studies [59]. In C4-2B, PC-3, and LNCaP cells, knockdown of CREB1 significantly reduced both mRNA and protein levels of CtIP, as confirmed by RT-qPCR and WB (Figs. 3i, j and S5b, c). Moreover, CREB1 knockdown impaired homologous recombination (HR) repair efficiency (Fig. 3k), confirming its regulatory role in the CtIP-mediated HR pathway. Based on predictions from the JASPAR database, we identified two high-potential binding motifs within the CtIP promoter region (chr18:22931328-22933428) (Fig. S5d).

In summary, this study demonstrates that CREB1 is a transcription factor activated by Abi that regulates CtIP expression.

### Abi and IR regulate CtIP expression by activating CREB1 transcriptional activity via phosphorylation

**Fig. 4:**
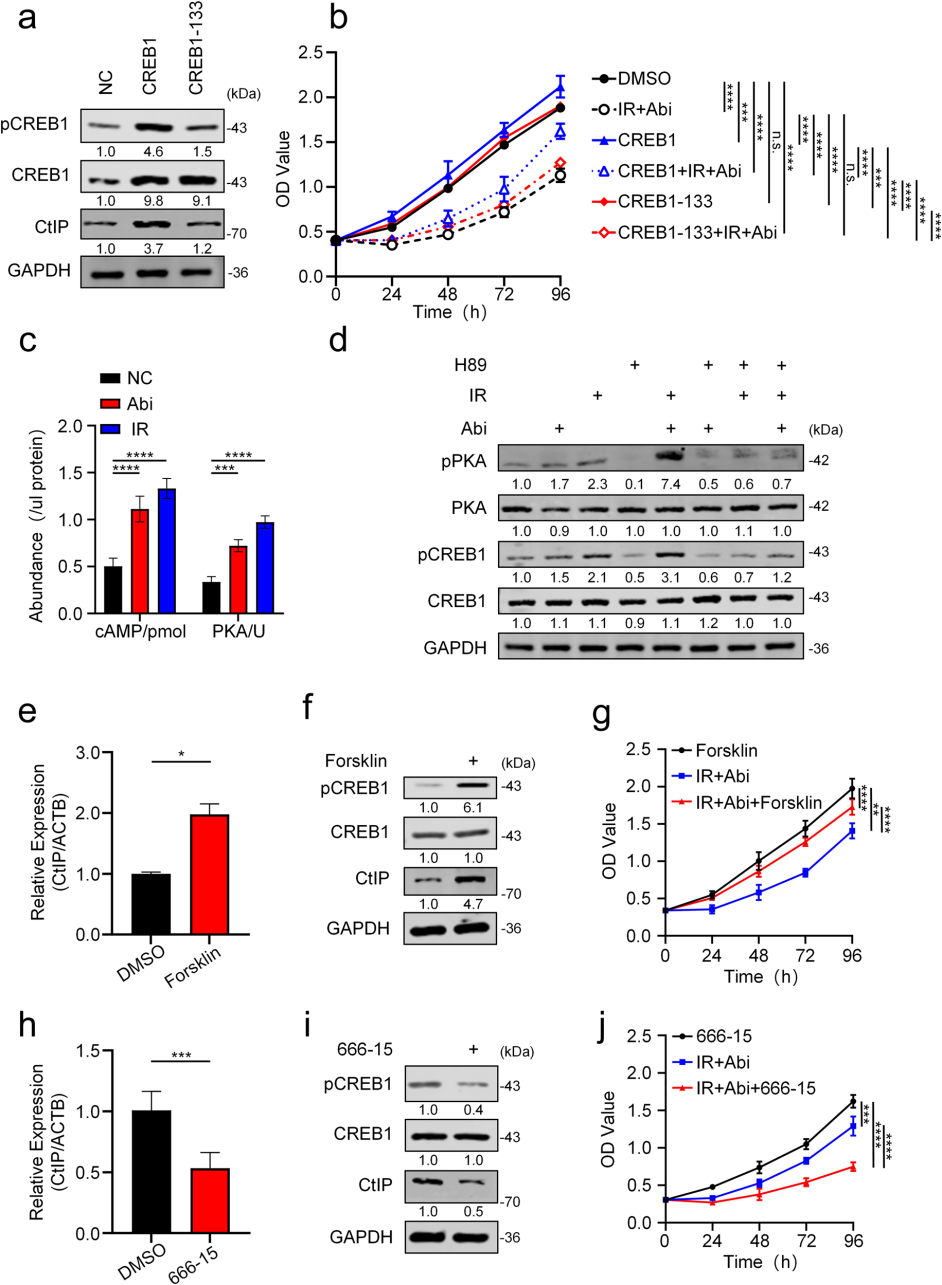
Abi and IR regulate CtIP expression by activating CREB1 transcriptional activity via phosphorylation. **a** C4-2B cells were transfected with the wild type CREB1 construct (CREB1), the CREB1 mutant construct, which contained a mutation at S133A (CREB1-133), or vehicle. 48 h after transfection. Western blot analyses of the protein levels of CREB1, p-CREB1 and CtIP in cells before and after transfection. Changes in intracellular cAMP levels and PKA activity were measured in C4-2B cells using corresponding assay kits following treatment with control, Abi (10 μM), or IR (2 Gy). Molecular levels were determined 48 h after treatment or cell incubation. ^***^P < 0.001, ^****^P < 0.0001, unpaired *t*-test. **b** CCK8 OD value (measured every 24 h for 96 h) and statistical analysis were assessed in C4-2B cells under six treatment conditions: control group; IR (2 Gy) combined with Abi (10 μM, added 48 h prior to IR); CREB1 or CREB1-133 overexpression; CREB1 or CREB1-133 overexpression combined with IR and Abi. n.s., no significance, ^***^P < 0.001, ^****^P < 0.0001, ANOVA. **c** Changes in intracellular cAMP levels and PKA activity were measured in C4-2B cells using corresponding assay kits following treatment with control, Abi (10 μM), or IR (2 Gy). Molecular levels were determined 48 h after treatment or cell incubation. ^***^P < 0.001, ^****^P < 0.0001, unpaired *t*-test. **d** Western blot analysis of the indicated proteins was performed in C4-2B cells following exposure to IR (2 Gy) and a 48-h incubation, with or without pretreatment with Abi (10 μM, 48 h prior to IR) and/or H89 (10 μM, (a protein kinase A inhibitor, 0.5 h prior to IR). RT-qPCR (**e**) and Western blot (**f**) analyses were performed to determine the expression levels of the indicated mRNAs and proteins in C4-2B cells treated with Forskolin (10 μM) for 24 h and 48 h.^*^P < 0.01, unpaired *t*-test. **g** CCK8 OD value (measured every 24 h for 96 h) and statistical analysis of C4-2B cells following a 96-h incubation after treatment. Treatment conditions included: Forskolin (10 μM); IR combined with Abi (10 μM, added 48 h prior to IR); IR combined with Abi and Forskolin (added 0.5 h prior to IR). ^**^P < 0.01, ^****^P < 0.0001, ANOVA. RT-qPCR (**h**) and Western blot (**i**) analyses were performed to determine the expression levels of the indicated mRNAs and proteins in C4-2B cells treated with 666-15 (0.5 μM) for 24 h and 48 h. ^***^P < 0.001, unpaired *t*-test. **j** CCK8 OD value (measured every 24 h for 96 h) and statistical analysis of C4-2B cells following a 96-h incubation after treatment. Treatment conditions included: 666-15 (0.5 μM); IR combined with Abi (10 μM, added 48 h prior to IR); IR combined with Abi and 666-15 (added 0.5 h prior to IR). ^***^P < 0.001, ^****^P < 0.0001, ANOVA.

As a classic transcription factor, CREB1 possesses a critical characteristic: phosphorylation at serine 133 (Ser133) [60] results in the formation of phospho-CREB1 (pCREB1), leading to the activation of its transcriptional activity (prior studies have confirmed that Abi activates CREB1 transcriptional activity via its phosphorylation, thereby promoting CtIP expression). To validate this, we constructed a CREB1 mutant plasmid (CREB1-S133A), in which Ser133 was substituted with alanine. Wild-type CREB1, the CREB1-S133A mutant, and a control vector were overexpressed in C4-2B cells. The results showed that, compared to wild-type CREB1, overexpression of the CREB1-S133A mutant failed to upregulate CtIP expression (Fig. 4a). Similarly, overexpression of CREB1-S133A could not reverse the anti-proliferative effect of the combined Abi and IR treatment on C4-2B cells, compared to the wild-type CREB1 group (Fig. 4b). These findings highlight the crucial role of Ser133 phosphorylation in CREB1-mediated transcriptional activation of CtIP. Studies by Pan et al. [29] indicate that AA can induce CREB1 phosphorylation by elevating cyclic AMP (cAMP) levels. Furthermore, CREB1 participates in the DDR process via the core DDR regulator ATM [61], which can be activated by radiation (e.g., IR). To verify these findings and investigate the influence of both pathways on CREB1 phosphorylation, we utilized the PKA phosphorylation inhibitor H89 and the ATM phosphorylation inhibitor KU-60019. The results demonstrated that both Abi and RT could mediate CREB1 phosphorylation via the cAMP-PKA signaling pathway (Fig. 4c, d), while RT also induced CREB1 phosphorylation via the ATM pathway (Fig. S6a). Consequently, when Abi and RT are combined, inhibition of either PKA or ATM phosphorylation is insufficient to completely block CREB1 phosphorylation due to the compensatory activity of the other pathway (Figs. 4d and S6a). To circumvent this, we employed the CREB1 phosphorylation inhibitor 666-15. To further validate the impact of these two phosphorylation pathways and 666-15 on CtIP, we treated C4-2B cells with Forskolin (a cAMP activator), KU-60019, and 666-15, respectively. Our results demonstrated that Forskolin treatment significantly upregulated CtIP expression in C4-2B cells (Fig. 4e, f) and concurrently counteracted the therapeutic efficacy of the IR and Abi combination (Fig. 4g). Conversely, KU-60019, by inhibiting IR-induced ATM phosphorylation and the subsequent phosphorylation of CREB1, attenuated CtIP expression (Fig. S6b, c). However, its combination with 666-15 enhanced the effect of Abi-IR therapy (Fig. S6d). In contrast, 666-15 directly inhibited CREB1 phosphorylation (Fig. 4h, i), thereby reducing CtIP expression (Fig. 4j) to potentiate the Abi-IR combined treatment.

In summary, our study demonstrates that in metastatic prostate cancer cells, both Abi (via the cAMP-PKA signaling pathway) and IR (through both the cAMP-PKA signaling and ATM phosphorylation pathways) phosphorylate CREB1 at Ser133, thereby activating its transcriptional activity and subsequently promoting CtIP expression.

### TETs promote CREB1-CtIP binding by demethylating CpG islands

**Fig. 5:**
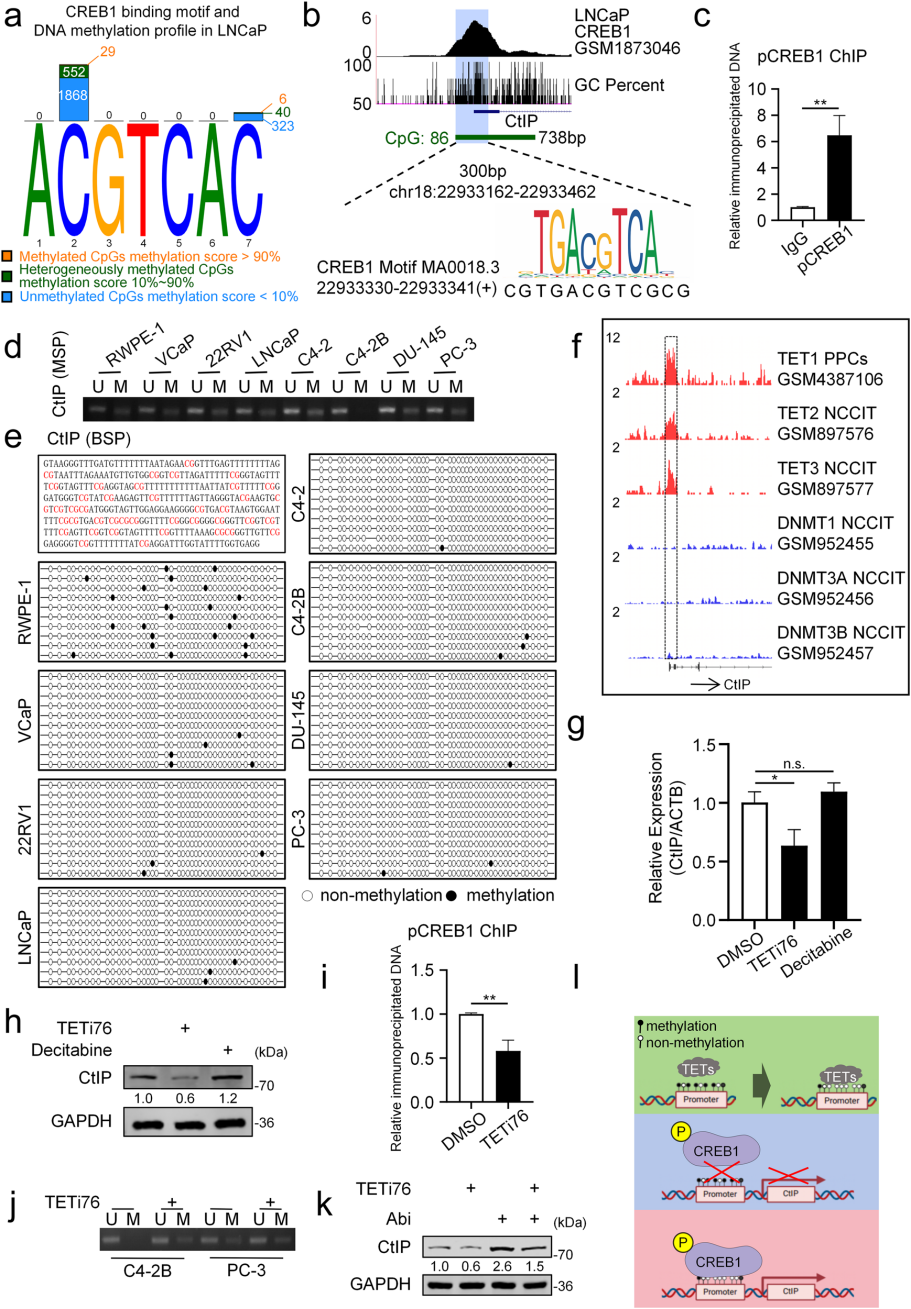
TETs promote CREB1-CtIP binding by demethylating CpG islands. **a** Analysis of CpG methylation levels at CREB1 transcription factor binding motifs in LNCaP cells was performed using the MethMotif database. **b** Through integrated analysis of CREB1 ChIP-seq data and the CtIP promoter binding region (Blue highlighted area), a 300-bp sequence (chr18:22933162-22933462) spanning the overlap between the highest GC-content region (GC%) and a CpG island (738 bp) was identified. Notably, this region coincides with a previously predicted binding motif (chr18:22933330-22933341). **c** ChIP-qPCR demonstrated that in C4‑2B cells, the binding of pCREB1 to the examined promoter region (chr18:22933162‑22933462) of CtIP was significantly stronger than that in the IgG control group. ^**^P < 0.01, unpaired *t*-test. **d** A methylation-specific PCR (MSP) assay was conducted to verify the methylation sites of the CtIP promoter(chr18:22933162-22933462) in different prostate cancer cell lines and the normal prostate epithelial cell line (RWPE-1). **e** Bisulfite sequencing PCR (BSP) analysis was performed to compare the methylation status of the CtIP promoter(chr18:22933162-22933462) in different prostate cancer cell lines and RWPE-1. **f** The ChIP-seq results showed the binding of TET1, TET2, TET3, DNMT1, DNMT2, DNMT3 to the CtIP promoter region. RT-qPCR (**g**) and Western blot (**h**) analyses were performed to determine the expression levels of the indicated mRNAs and proteins in C4-2B cells treated with TETi76(10 μM) or Decitabine (0.2 μM) for 24 h and 48 h. n.s., no significance, ^*^P < 0.05, unpaired *t*-test. **i** ChIP-qPCR experiments after treated with TETi76(10 μM) for 48 h revealed that pCREB1 had significantly higher binding intensity to the promoter region (chr18:22933162‑22933462) of CtIP than the control group (DMSO) in C4-2B cells. ^**^P < 0.01, unpaired *t*-test. **j** MSP assay was conducted to verify the methylation sites of the CtIP promoter (chr18:22933162-22933462) after treated with TETi76 (10 μM) for 48 h in C4-2B and PC-3. **k** Western blot analysis of indicated proteins in C4-2B cells following a 48-h incubation after treatment. Treatment conditions included: control group; TETi76(10 μM); Abi (10 μM); TETi76 combined with Abi. **l** Schematic diagram delineating the mechanistic interplay between TET family-mediated modulation of CtIP promoter methylation and its regulatory effects on pCREB1 binding.

While we have investigated the regulation of target gene expression by transcription factors, DNA methylation in the promoter region of these target genes serves as a key determinant affecting transcription factor binding [62]. To further investigate the transcriptional regulation between CREB1 and CtIP, MethMotif database analysis revealed hypermethylated CpGs at CREB1 binding motifs in LNCaP cells (Fig. 5a). TCGA data analysis showed significantly reduced methylation in the CtIP promoter region of prostate cancer compared to normal prostate tissue (Fig. S7a). UCSC and GEO database analyzes identified a 300 bp sequence (chr18:22933162-22933462) overlapping the CtIP promoter’s highest CREB1 binding peak, GC-rich region, and CpG island (chr18:22933157-22933894, 738 bp) (Fig. 5b, Blue highlighted area). Coincidentally, the integration of our prior predictions revealed that the first predicted binding locus (chr18:22933330-22933341) (Fig. S5d) resides within this overlapping genomic region (Fig. 5d). ChIP-qPCR analysis of this 300 bp region revealed a significant increase in pCREB1 binding occupancy at this site (chr18:22933330-22933341) compared with the control group (Fig. 5c). Methylation-specific PCR (MSP) of the 300 bp region revealed incomplete methylation in normal prostate cells (RWPE-1) and prostate cancer cell lines (Figs. 5d and S7b). Bisulfite sequencing PCR (BSP) further demonstrated significantly reduced methylation in cancer cells compared to normal cells (Figs. 5d and S7c).

These results confirm hypomethylation of the CtIP promoter in prostate cancer. DNA methylation is regulated by DNA methyltransferases (DNMTs) and Ten-Eleven Translocation enzymes (TETs). GEO ChIP-seq data (GSM4387106, GSM897576, etc.) showed TET1/2/3, but not DNMTs, binding at the CtIP promoter (Fig. 5f). Analysis of TCGA data further revealed that compared to normal prostate tissues, primary prostate cancer tissues exhibited no significant alteration in TET1 expression, while TET2 was significantly downregulated and TET3 markedly upregulated (Fig. S7d). Given the complex mechanisms underlying DNA demethylation [63–66], we employed the pan-TET inhibitor TETi76 (targeting TET1, TET2, and TET3) and the DNMT inhibitor Decitabine (targeting DNMT1, DNMT3A, and DNMT3B) to investigate the regulatory effects of TET and DNMT enzymes on CREB1 transcription. TETi76 reduced CtIP expression (Fig. 5g, h) and pCREB1 binding (Fig. 5i), while decitabine showed no effect. MSP confirmed increased methylation at the CREB1 binding site after TETi76 treatment in C4-2B and PC-3 cells (Figs. 5j and S7e). Consistent with this, Western blot analysis revealed that the TET inhibitor TETi76 abolished the ability of Abi to promote CtIP expression (Fig. 5k). Although TETi76 and decitabine are not CtIP-specific, these findings highlight TET-mediated demethylation’s role in CREB1-CtIP regulation.

In summary, TETs demethylate methylated cytosines at CpG islands in the CtIP promoter, facilitating binding of phosphorylated CREB1 and enhancing transcriptional activation (Fig. 5l).

### 666-15 enhances radiosensitivity in prostate cancer cells by inhibiting CREB1 phosphorylation

**Fig. 6:**
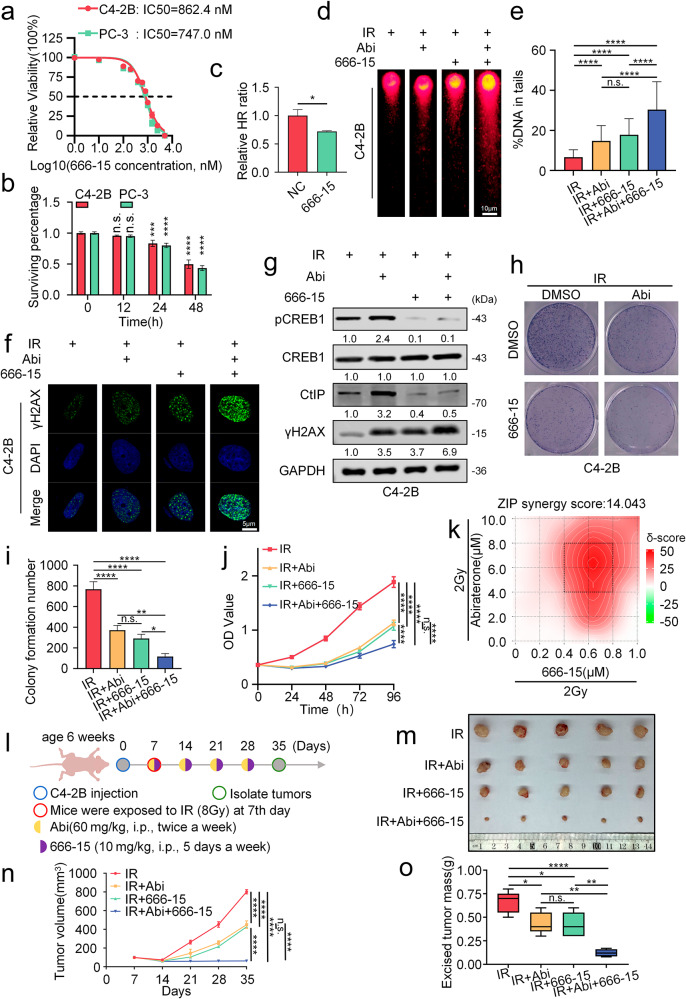
666-15 enhances radiosensitivity in prostate cancer cells by inhibiting CREB1 phosphorylation. **a** To determine the IC50 values of 666-15 in prostate cancer cell lines C4-2B and PC-3, optical density values were measured at 72 h post-treatment across a range of concentrations using the CCK-8 assay, followed by statistical analysis. **b** In C4-2B and PC-3, cell viability was systematically evaluated after treatment with 666-15 (0.8 μM) for different durations (0 h, 12 h, 24 h and 48 h). n.s., no significance, ^***^P < 0.001, ^****^P < 0.0001, unpaired *t*-test. **c** HR repair reporter assay Control and treated with 666-15 (0.5 μM) for 48 h in HeLa cells. The population of mCherry+ cells in BFP+ cells was analyzed. Data from three biological replicates were presented. ^*^P < 0.05, unpaired *t*-test. Comet assay (**d**) and statistical analysis (**e**) were assessed in C4-2B cells following a 48-h incubation after treatment. Treatment conditions included: IR (2 Gy); IR combined with Abi (10 μM, added 48 h prior to IR); IR combined with 666-15(0.5 μM, added 0.5 h prior to IR); IR combined with Abi and 666-15. DNA damage quantified via % DNA in tails. Each data point represents at least 50 cells counted. Scale bar, 10 μm. n.s., no significance, ^****^P < 0.0001, ANOVA. **f** Representative images of γH2AX foci in C4-2B cells following a 48-h incubation after treatment. Treatment conditions included: IR (2 Gy); IR combined with Abi (10 μM, added 48 h prior to IR); IR combined with 666-15(0.5 μM, added 0.5 h prior to IR); IR combined with Abi and 666-15. Scale bar, 5 μm. **g** Western blot analysis of the indicated proteins was performed in C4-2B cells following a 48-h incubation after treatment. Treatment conditions included: IR (2 Gy); IR combined with Abi (10 μM, added 48 h prior to IR); IR combined with 666-15(0.5 μM, added 0.5 h prior to IR); IR combined with Abi and 666-15. Representative images (**h**) and quantitative analysis (**i**) of colony formation assays in C4-2B cells following a 14-day incubation after treatment. Treatment conditions included: IR (2 Gy); IR combined with Abi (10 μM, added 48 h prior to IR); IR combined with 666-15 (0.5 μM, added 0.5 h prior to IR); IR combined with Abi and 666-15. n.s., no significance, ^*^P < 0.05, ^**^P < 0.01, ^****^P < 0.0001, ANOVA. **j** CCK8 OD value (measured every 24 h for 96 h) and statistical analysis were assessed in C4-2B cells under three treatment conditions: IR (2 Gy); IR combined with Abi (10 μM, added 48 h prior to IR); IR combined with 666-15(0.5 μM, added 0.5 h prior to IR); IR combined with Abi and 666-15. n.s., no significance, ^****^P < 0.0001, ANOVA. **k** Synergistic interactions between Abi and 666-15 under IR(2 Gy) conditions were quantitatively analyzed using SynergyFinder 3.0 with the Zero Interaction Potency (ZIP) model. Synergistic effects were defined as ZIP scores >10. The heatmap axes display concentration gradients corresponding to the maximal synergy region (demarcated by a black rectangle). **l** The specific process of xenotransplantation in nude mouse models. The representative image illustrates the C4-2B tumor xenograft model (**m**) in male BALB/c nude mice (*n* = 5) and the statistical data of tumor volume (**n**) and weight (**o**) under various treatments. The treatment groups were as follows: IR (8 Gy) irradiation alone; IR plus Abi (60 mg/kg, i.p., twice weekly, Abiraterone was administered 4 h prior to IR.); IR plus 666-15 (10 mg/kg, i.p., 5 days a week, 666-15 was administered 4 h prior to IR.); and IR combined with both Abi and 666-15. n.s., no significance, ^*^P < 0.05, ^**^P < 0.01, ^****^P < 0.0001, ANOVA.

To further investigate the impact of the CREB1 phosphorylation inhibitor 666-15, identified in this study, on the combined treatment of Abi and IR, we conducted a series of related experiments. The results revealed that the IC50 values of 666-15 in prostate cancer cell lines C4-2B and PC-3 were 862.4 nM and 747.0 nM (Fig. 6a), respectively. Cytotoxic effects began to manifest at 24 h post-treatment, reaching approximately half-maximal inhibition around 48 h (Fig. 6b); 666-15 treatment significantly reduced cellular HR repair efficiency (Fig. 6c). In C4-2B cells, the addition of 666-15 significantly enhanced the efficacy of RT compared to the group treated with IR and Abi alone, as evidenced by: increased DNA damage demonstrated by the comet assay (Fig. 6d, e), elevated levels and fluorescence intensity of γH2AX protein (Fig. 6f, g), and a significant inhibition of cell growth (Fig. 6h, i). In PC-3 cells, which exhibit AR deficiency and therefore no increased radiosensitivity from Abi, the addition of 666-15 still significantly improved the therapeutic outcome of radiotherapy (Fig. S8a–g). Analysis using SynergyFinder 3.0 software indicated a synergistic interaction (ZIP synergy score = 14.043 > 10) between Abi and 666-15 under IR conditions in C4-2B cells, rather than a merely additive effect (Fig. 6k). These in vitro findings were validated in a C4-2B cell xenograft mouse model. Compared to the group treated with IR combined with Abi, the group receiving the triple combination of IR, Abi, and 666-15 exhibited significantly reduced tumor size, weight, and volume (Fig. 6l–o). Immunohistochemistry (IHC) analysis of tumor tissues showed consistent results with the in vitro experiments regarding the expression levels of pCREB1, CREB1, and CtIP (Fig. S8h, i). To evaluate the safety of the combination therapy, we first examined the viability of normal prostate epithelial cells (RWPE‑1). Cells were treated with 666‑15 at varying concentrations, followed by the combination of Abi and IR, or exposed to a fixed concentration of 666‑15 combined with Abi and IR for different durations (using drug concentrations previously demonstrated to exert effective killing in prostate cancer cell lines). After accounting for the cytotoxic effect of IR, the results showed no significant difference in the killing of RWPE‑1 cells either with different concentrations of 666‑15 combined with Abi, or with the same concentration applied for different treatment periods (Fig. S9a, b). Next, we collected major potential target organs (heart, liver, spleen, lung, and kidney) that are most susceptible to drug‑ and metabolite‑related toxicity. H&E staining revealed no obvious damage in the combination therapy group compared with the control group (Fig. S9c). Similarly, liver function (assessed by serum aspartate aminotransferase (AST) and alanine aminotransferase (ALT) levels) and kidney function (assessed by blood urea nitrogen (UREA) and creatinine (CREA) levels) showed no significant differences from the control group (Fig. S9d–g).

In summary, 666-15 enhances the radiosensitizing effect of Abi in metastatic prostate cancer cells. Notably, even in PC-3 cells where Abi alone is ineffective, the combination of 666-15 with IR effectively improves radiosensitivity. Our findings provide a novel strategy for the treatment of prostate cancer, particularly in the context of radiotherapy.

## Discussion

In recent years, for the treatment of prostate cancer, the combination of abiraterone and radiotherapy has been recommended in multiple clinical guidelines [15–18] and extensively investigated, owing to challenges such as disease progression, emergence of drug resistance, and limited efficacy of monotherapy. However, multiple clinical studies have indicated that in advanced prostate cancer, particularly metastatic prostate cancer, this combination regimen does not appear to achieve ideal outcomes (Fig. 1a) [19–23]. Compared with the control group, the combination group showed no significant improvement in overall survival (OS), with only partial benefits observed in certain progression‑free survival endpoints (such as Biochemical PFS and Radiographic PFS (LVD)). Certainly, numerous underlying factors may contribute to this phenomenon, including loss of AR expression, emergence of splice variants (AR‑Vs), and resistance to Abi. These factors, as well as the combination with Abi, are based on AR‑mediated regulation of DDR‑related genes. Thus, a question arises: Are there non‑AR‑regulated DDR‑related genes that are also modulated by Abi and radiotherapy, thereby influencing the efficacy of this combined therapeutic approach?

**Fig. 7:**
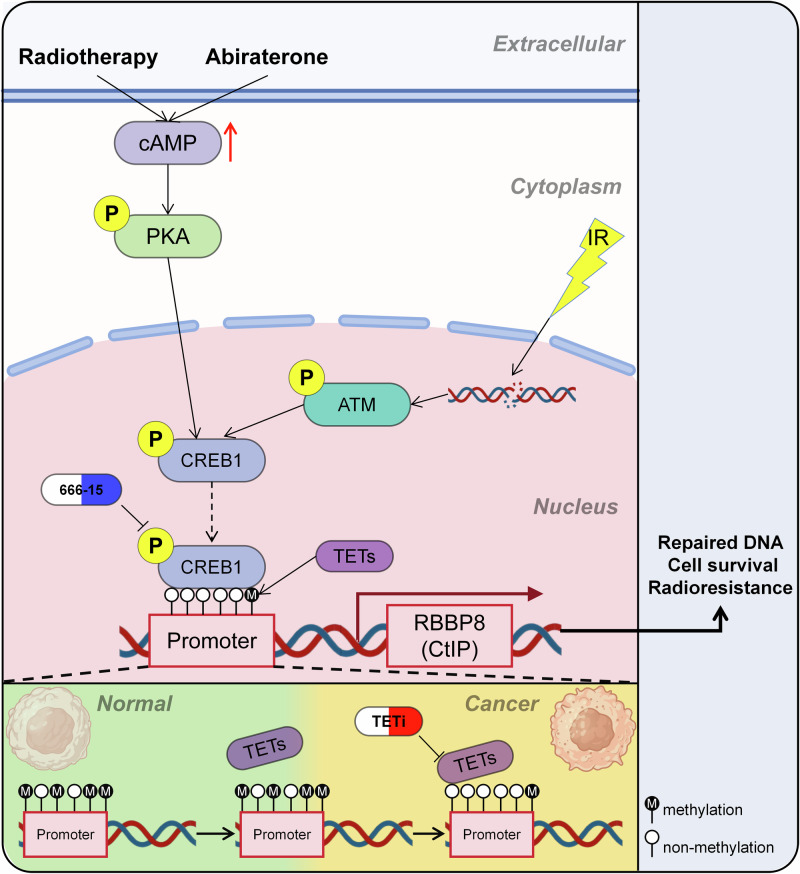
Targeted inhibition of the CREB-CtIP axis enhances sensitivity to abiraterone when combined with radiotherapy. Both Abiraterone and ionizing radiation (IR) activate CREB1 phosphorylation through cAMP/PKA signaling axis, while IR additionally stimulates ATM kinase-mediated CREB1 phosphorylation via DNA damage response. Phosphorylated CREB1 (pCREB1) significantly enhances CtIP transcriptional activity. The resultant CtIP protein participates in homologous recombination (HR) repair pathway, thereby compromising radiosensitivity and enhancing prostate cancer cell survival. During prostate carcinogenesis, TET (Ten-Eleven Translocation) family enzymes catalyze active demethylation in the CtIP promoter region, establishing a hypomethylated chromatin state that facilitates pCREB1 binding accessibility.

Based on this hypothesis, our study experimentally revealed that the DDR protein CtIP, although induced to high expression by both Abi and radiotherapy, is not regulated by AR. Moreover, elevated CtIP expression attenuates the therapeutic efficacy of the Abi and IR combination. Through further investigation, we not only identified CREB1 as the key transcription factor driving CtIP expression, but also found that high CtIP expression is associated with poor prognosis. Simultaneously, we discovered that both Abi and IR can activate the transcriptional activity of CREB1. Specifically, Abi and IR promote extracellular cAMP expression, which in turn activates PKA via phosphorylation. Activated PKA then translocates into the nucleus and phosphorylates CREB1, thereby enhancing its transcriptional activity and subsequently upregulating CtIP expression. Additionally, IR can activate ATM (via phosphorylation) through DNA damage, and activated ATM also phosphorylates and activates CREB1. Consistent with previous findings, our study confirms that both activation pathways involve phosphorylation at serine 133 (Ser133) of CREB1. Building on this, we further validated experimentally that the CREB1 phosphorylation inhibitor 666-15 sensitizes prostate cancer cells to the combination of Abi and IR. Furthermore, during prostate cancer progression, TET family enzyme-mediated DNA demethylation at the CtIP promoter region facilitates an open chromatin conformation, thereby creating a more favorable condition for CREB1 binding (Fig. 7). In summary, this study reveals a novel mechanism by which inhibition of the CREB1-CtIP axis can enhance the efficacy of combined Abi and radiotherapy, providing a potential new strategy for radiosensitization in prostate cancer.

As a key molecule in DDR, CtIP plays a tumor‑suppressive role by preventing oncogenic mutations and genomic instability resulting from erroneous repair of DNA DSBs, primarily through its central function in homologous recombination (HR). However, the same mechanism can also promote tumorigenesis, reflecting the “dual‑faced” nature of CtIP in cancer biology [67]. For example, low CtIP expression has been associated with increased aggressiveness and poor response to hormone therapy in breast cancer [68, 69]. Similarly, low CtIP expression is associated with a favorable prognosis in bladder cancer [70]. whereas in luminal breast cancer, reduced CtIP levels correlate with better efficacy of DNA‑damaging chemotherapy [68]. Our study further revealed that in prostate cancer, CtIP expression is negatively correlated with prognosis, illustrating that the prognostic significance of CtIP expression varies across different types of cancer. The work by Eke et al. demonstrated that in prostate cancer cells (PC‑3), CtIP and XPC remain elevated relative to other DDR genes even 2 months after a single exposure to irradiation (10 Gy) [71]. Moreover, CtIP participates in DDR in an ATM‑phosphorylation‑dependent manner [72] and closely interacts with the DSB‑sensing Mre11‑Rad50‑NBS1 (MRN) complex [73]. Additionally, inhibition of CtIP‑mediated replication fork protection has been shown to induce γH2AX‑driven fork degradation [74]. Collectively, these findings indicate that CtIP not only responds to DNA damage in the short term but also exhibits long‑term persistence, maintaining close functional links with key DDR nodal players such as ATM, MRN, and γH2AX. Given these properties, CtIP holds promise as a predictive biomarker for treatment response in specific patient populations, particularly as an indicator of radiosensitization.

Previous studies on the regulatory mechanisms of CtIP expression have primarily focused on post-translational modifications such as phosphorylation [72], ubiquitination [75, 76], and deubiquitination [77]. Starting from a clinical perspective, this study has stepwise elucidated that CREB1 functions as a transcriptional regulator of CtIP. CREB1 is a key transcription factor involved in modulating multiple biological processes and has also been identified as a direct driver in the pathogenesis of various cancers [24, 26]. A hallmark of CREB1 is its transcriptional activation via phosphorylation at Ser133, mediated by kinases such as ATM, PKA, and PKB [25]. Elevated pCREB1 levels are observed in poorly differentiated prostate cancer and bone metastasis samples compared to benign tissues, with its expression positively correlating with tumor grade and metastatic burden [27], underscoring CREB1’s pivotal role in cancer progression and metastasis. Moreover, studies have shown that during the treatment of prostate cancer cells, fractionated IR can promote CREB1 phosphorylation and drive their differentiation into neuroendocrine (NE)-like cells [28], which may further contribute to tumor recurrence and reduced therapeutic sensitivity. This further underscores the importance of inhibiting CREB1 phosphorylation in prostate cancer.

DNA methylation at CREB1 binding sites critically influences its transcriptional activity [62]. Our study revealed hypomethylation in the CtIP promoter during prostate cancer development, facilitated by TET enzymes (TET1, TET2, TET3), which enhances CREB1 binding. While TET1 downregulation promotes prostate cancer cell growth and metastasis, and TET2 interacts with AR to influence disease progression, DNA methylation exerts complex, context-dependent roles in tumorigenesis [63–66]. This study specifically demonstrates that TET-mediated demethylation at the CtIP promoter facilitates CREB1 binding, though the precise mechanisms and enzyme contributions require further investigation.

Previous studies have revealed that resistance to AA is associated with phosphorylation of pCREB1 [29]. Based on this, we established abiraterone‑resistant cell lines and experimentally demonstrated that adding 666‑15 to the combination of Abi and radiotherapy in these cells also restored sensitivity to Abi (Fig, S10a, b), which may represent another mechanism by which 666‑15 enhances radiosensitivity. Additionally, 666‑15 was shown to reverse resistance to BET inhibitors in retinoblastoma protein (RB)‑deficient cells [78]. Similarly, at the beginning of this study, we observed that besides CtIP, the expression levels of *FANCI*, *USP1*, *NBN*, and *LIG3* were also upregulated upon Abi treatment, but decreased after AR knockdown. Combining previous findings with analysis of ChIP‑seq database data, we found that these genes are not only transcriptionally regulated by AR, but CREB1 may also act as their transcription factor (Fig. S10c). Based on this, we hypothesize that CREB1, like AR, transcriptionally regulates a series of DDR‑related genes [12], many of which are dually regulated by both AR and CREB1. The variable expression patterns of these genes after Abi treatment—some increased while others decreased—may result from the combined regulation of both factors or the involvement of other regulatory mechanisms. This further highlights the specificity of targeting the CREB1‑CtIP axis to enhance radiosensitivity, given that CtIP is solely regulated by CREB1. Furthermore, consistent with our experimental findings (Figs. S1g–h and S2a), while abiraterone exerted a radiosensitizing effect in AR‑positive cell lines (C4‑2B), it not only failed to enhance radiosensitivity in AR‑negative PC‑3 cells but even showed a tendency toward antagonism (though without reaching statistical significance). However, upon addition of 666‑15, radiosensitization was observed in both C4‑2B and PC‑3 cells (Figs. 6 and S8). These findings point to future research directions, such as investigating the set of genes regulated by CREB1 in DDR and their associated DDR pathways. Given these characteristics, it is worthwhile to further explore the combination of 666‑15 with PARP inhibitors (PARPi) to induce synthetic lethality, which may represent a promising therapeutic strategy. Thus, 666‑15 demonstrates significant potential for clinical application in the treatment of prostate cancer.

Anyway, this study proposes a novel therapeutic strategy for advanced prostate cancer: targeting the CREB1-CtIP axis to improve radiotherapy efficacy or AA combination therapy, independent of AR status. This approach offers new insights for managing complex prostate cancer cases, particularly those resistant to conventional therapies.

## Conclusion

This study provides novel mechanistic evidence demonstrating that transcription factor CREB1 positively regulates CtIP expression in prostate cancer, with Abi and radiation therapy (RT) exerting their effects through phosphorylation-dependent activation of CREB1 to enhance its transcriptional activity. Furthermore, we reveal that hypomethylation of CpG islands in the CtIP promoter region during prostate cell malignant transformation critically facilitates this regulatory process. Collectively, our findings establish CtIP as a pivotal DDR mediator in prostate cancer therapeutics. The CREB1/CtIP signaling axis represents a promising therapeutic target for advanced prostate cancer, offering new directions for treatment optimization.

## Supplementary information

Supplementary material

Supplementary Figure 1

Supplementary Figure 2

Supplementary Figure 3

Supplementary Figure 4

Supplementary Figure 5

Supplementary Figure 6

Supplementary Figure 7

Supplementary Figure 8

Supplementary Figure 9

Supplementary Figure 10

Original western blots

Original Table 1

Original Table 2

## Data Availability

The raw data generated in this study are available upon request from the corresponding author.
