# Supplementary material for: Targeted inhibition of the CREB1-CtIP axis enhances the efficacy of abiraterone combined with radiotherapy in prostate cancer
Source: Cell Death Dis. 2026 Mar 30;17(1):435. doi: 10.1038/s41419-026-08633-0 (PMC13158305; doi:10.1038/s41419-026-08633-0)
Supplement: Supplementary file 1 — Supplementary material [file 41419_2026_8633_MOESM1_ESM.doc]

**Targeted Inhibition of the CREB1-CtIP Axis Enhances the Efficacy of Abiraterone Combined with Radiotherapy in Prostate Cancer**

Xu Han, Liang Song, Yuankang Feng, Zihao Wang, Lina Wang, Ruoyang Liu, Yu Liu, Ningyang Li, Saiyu Ma, Fubo Lu, Jinjian Yang, Zhenlin Huang, Zhankui Jia

**Files included in supplementary information**

**Supplementary Figure 1, 2 (Supplemental to Figure 1)** CtIP is involved in the administration of the abiraterone and RT combination therapy.

**Supplementary Figure 3 (Supplemental to Figure 2)** CtIP Promotes DDR via HR to Influence Radiosensitivity in Prostate Cancer Cells

**Supplementary Figure 4, 5 (Supplemental to Figure 3)** CREB1 Transcriptionally Regulates CtIP Expression

**Supplementary Figure 6 (Supplemental to Figure 4)** Abi and IR Regulate CtIP Expression by Activating CREB1 Transcriptional Activity via Phosphorylation

**Supplementary Figure 7 (Supplemental to Figure 5)** TETs Promote CREB1-CtIP Binding by Demethylating CpG Islands

**Supplementary Figure 8, 9 (Supplemental to Figure 6)** 666-15 Enhances Radiosensitivity in Prostate Cancer Cells by Inhibiting CREB1 Phosphorylation

**Supplementary Figure 10 (Supplemental to Discussion)**

**Supplementary Table S1.** Sequence information of shRNAs

**Supplementary Table S2.** Sequence information of primers for RT-qPCR

**Supplementary Table S3.** Sequence information of primers for MSP

**Supplementary Table S4.** Antibodies and other reagents and resources

**Supplementary Table S5.** The CREB1/CtIP top 100 co-expressed gene set (comprising genes with the highest co-expression and functional relevance to CREB1 and CtIP).


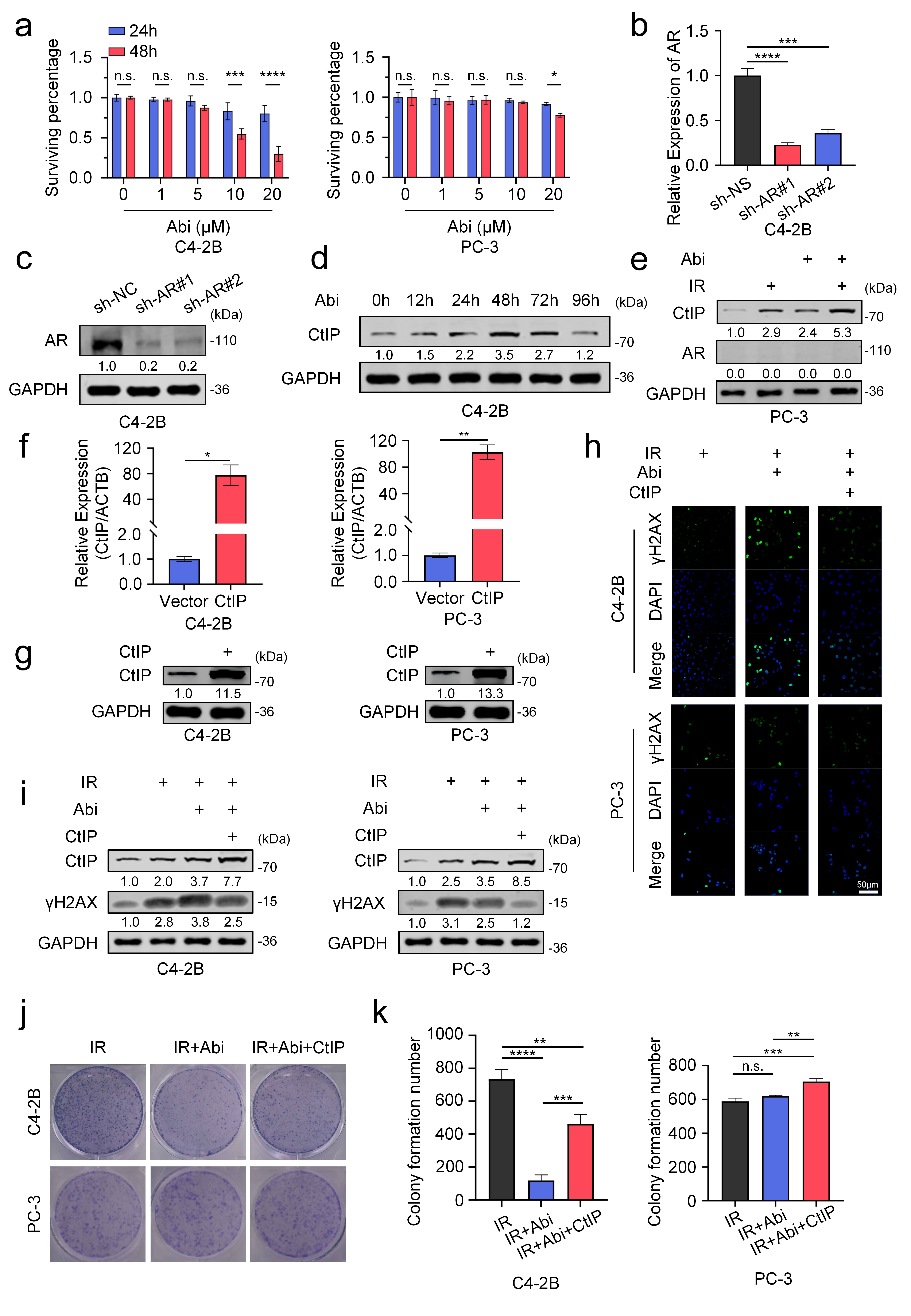


**Supplementary Figure 1**

(a) Cell viability was systematically assessed in prostate cancer C4-2B and PC-3 cell lines following 24- and 48-hour treatments with Abiraterone at escalating concentrations (0, 1, 5, 10, 20 μM), revealing time- and dose-dependent cytotoxic effects. n.s., no significance, * *P* < 0.01, *** *P* < 0.001, **** *P* < 0.0001, unpaired t-test.

(b, c) RT-qPCR and Western blot analysis showed the changes in AR mRNA (b) and protein (c) expression with/without knockdown of AR in C4-2B cells. *** *P* < 0.001, **** *P* < 0.0001, unpaired t-test.

(d) Western blot analysis delineated time-dependent modulation of CtIP protein expression in C4-2B cells following Abi (10 μM) treatment across sequential timepoints (0, 12, 24, 48, 72, and 96 hours).

(e) Western blot analysis of the indicated proteins was performed in PC-3 cells following a 48-hour incubation after treatment. Treatment conditions included: control group; IR (2 Gy); Abi (10 μM); IR combined with Abi (10 μM, added 48 hours prior to IR).

(f, g) RT-qPCR and Western blot analyses demonstrated successful CtIP overexpression at both transcriptional (mRNA) (f) and translational (protein)(g) levels in C4-2B and PC-3 cells, as evidenced by significant upregulation compared to empty vector controls. * *P* < 0.01, ** *P* < 0.01, unpaired t-test.

(h) Representative images of γH2AX(Ser139) foci in C4-2B and PC-3 cells following a 48-hour incubation after treatment. Treatment conditions included: IR (2 Gy); IR combined with Abi (10 μM, added 48 hours prior to IR); IR combined with Abi and CtIP overexpression (added 48 hours prior to IR). Scale bar, 50 μm.

(i) Western blot analysis of indicated proteins in C4-2B and PC-3 cells following a 48-hour incubation after treatment. Treatment conditions included: control group; IR (2 Gy); Abi (10 μM); IR combined with Abi (10 μM, added 48 hours prior to IR); IR combined with Abi and CtIP overexpression (added 48 hours prior to IR).

(j, k) Representative images(j) and quantitative analysis(k) of colony formation assays in C4-2B and PC-3 cells following a 14-day incubation after treatment. Treatment conditions included: IR (2 Gy); IR combined with Abi (10 μM, added 48 hours prior to IR); IR combined with Abi and CtIP overexpression (added 48 hours prior to IR). n.s., no significance, ** *P* < 0.01, *** *P* < 0.001, **** *P* < 0.0001, ANOVA.


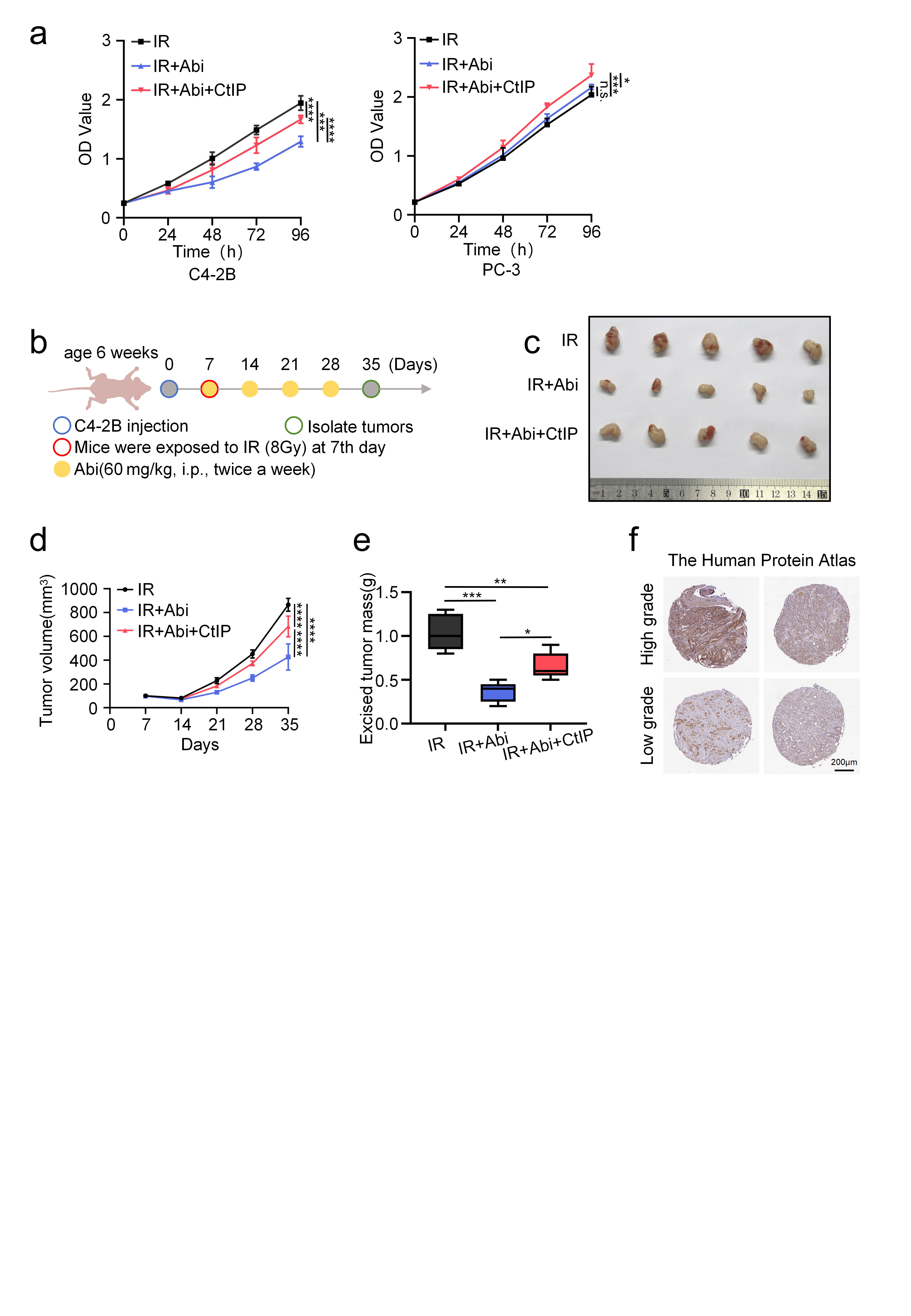


**Supplementary Figure 2**

1. CCK8 OD value (measured every 24 hours for 96 hours) and statistical analysis of C4-2B and PC-3 cells following a 96-hour incubation after treatment. Treatment conditions included: IR (2 Gy); IR combined with Abi (10 μM, added 48 hours prior to IR). n.s., no significance, * *P* < 0.05, *** *P* < 0.001, **** *P* < 0.0001, ANOVA.
2. The specific process of xenotransplantation in nude mouse models

(c-e) The representative image illustrates the C4-2B tumor xenograft model(c) established in male BALB/c nude mice (n=5), along with the corresponding statistical data of tumor volume(d) and weight(e) across different treatment groups. The groups included: IR (8 Gy) irradiation alone; IR combined with Abi (60 mg/kg, intraperitoneal injection, twice weekly, Abiraterone was administered 4 hours prior to IR.); and IR combined with both Abi and CtIP overexpression. * *P* < 0.01, ** *P* < 0.01, *** *P* < 0.001, **** *P* < 0.0001, ANOVA.

(f) IHC staining showed CtIP expression in different grades of prostate cancer (immunohistochemical data from the HPA database).


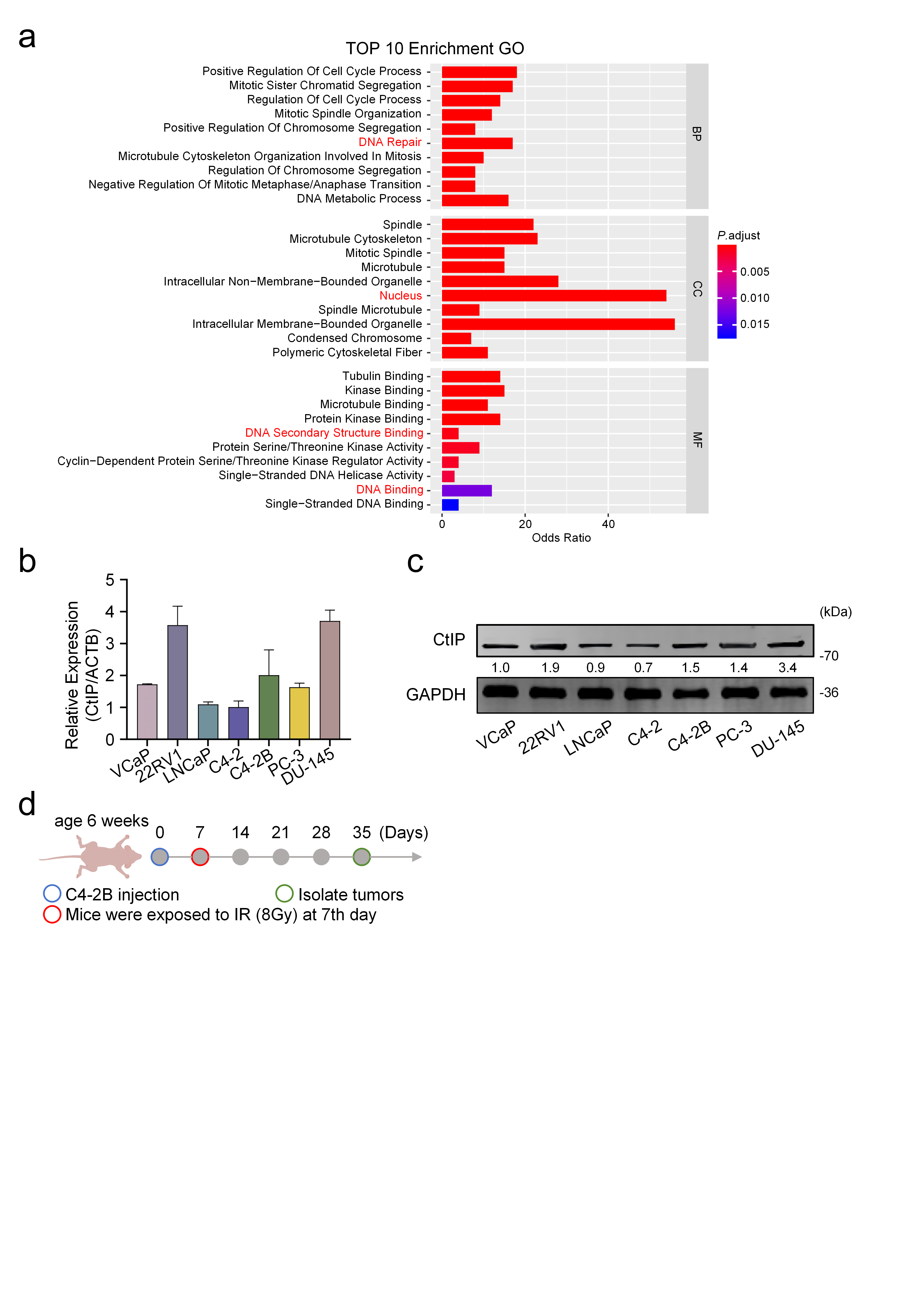


**Supplementary Figure 3**

1. Gene Ontology (GO) enrichment analysis was performed via the Enrichr database using a gene set comprising the top 100 genes exhibiting the highest co-expression with CtIP (identified by expanding its functionally associated gene set via the ARCHS4 RNA-seq gene-gene co-expression matrix).

(b, c) RT-qPCR (b) and Western blot analysis (c) were used to detect the expression of CtIP in different prostate cancer cell lines. One representative experiment of 3 independent experiments is shown.

(d) The specific process of xenotransplantation in nude mouse models.


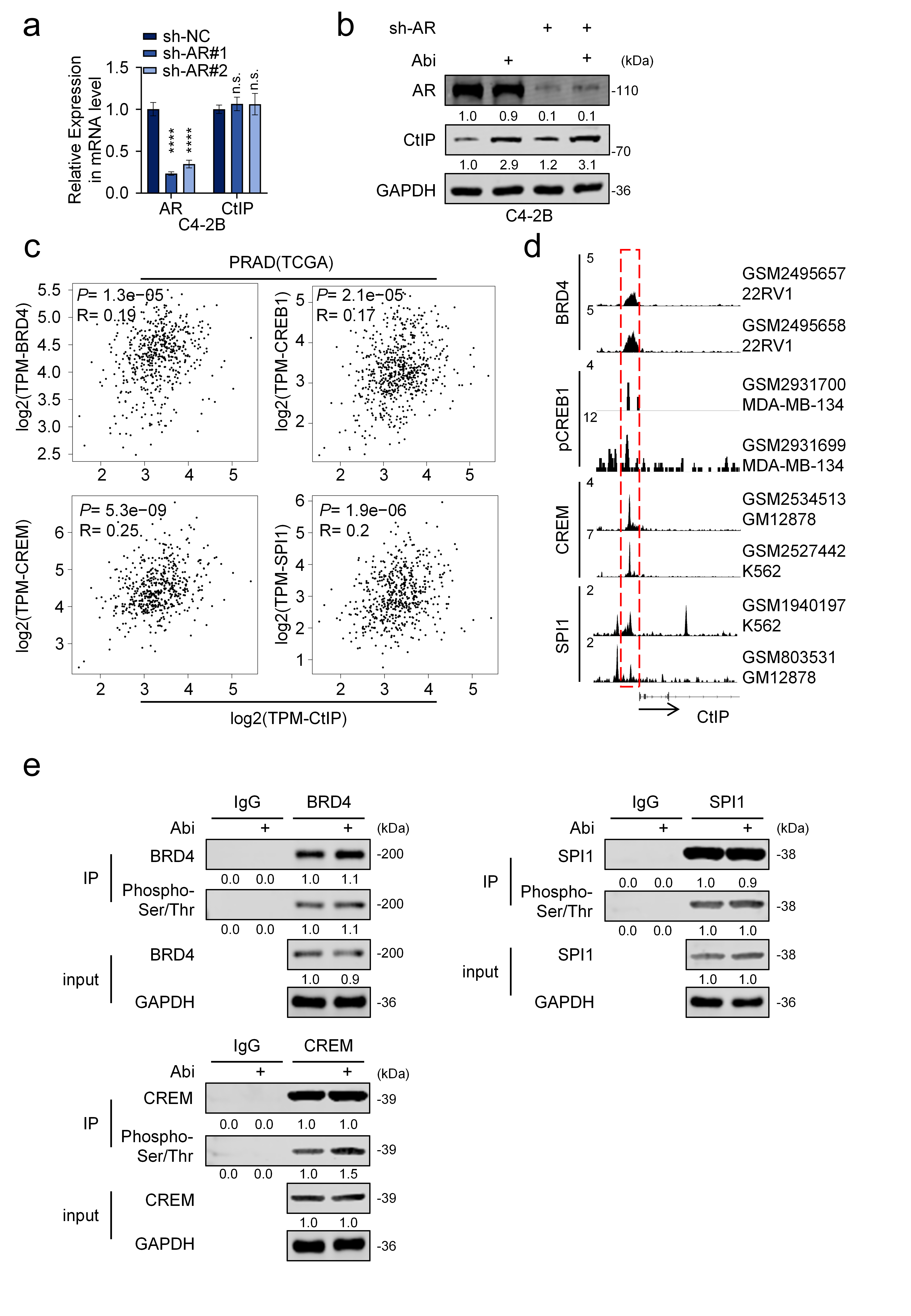


**Supplementary Figure 4**

(a) RT-qPCR analysis showed the changes in CtIP expression with/without knockdown AR in C4-2B cells. n.s., no significance, **** *P* < 0.0001, unpaired t-test.

(b) Western blot analysis of indicated proteins in C4-2B cells following a 48-hour incubation after treatment. Treatment conditions included: control group; Abi (10 μM); AR knockdown; AR knockdown combined with Abi.

(c) Correlation analysis of CtIP with BRD4, CREB1, CREM and SPI1 in Prostate adenocarcinoma (PRAD) from the TCGA database.

(d) The ChIP-seq results showed the binding of BRD4, pCREB1, CREM and SPI1 to the CtIP promoter region (red dotted line aera).

(e) Western blot analysis was performed to assess the phosphorylation levels of BRD4, CREM, and SPI1 in C4-2B cells via immunoprecipitation after a 48-hour treatment with Abi (10 μM).


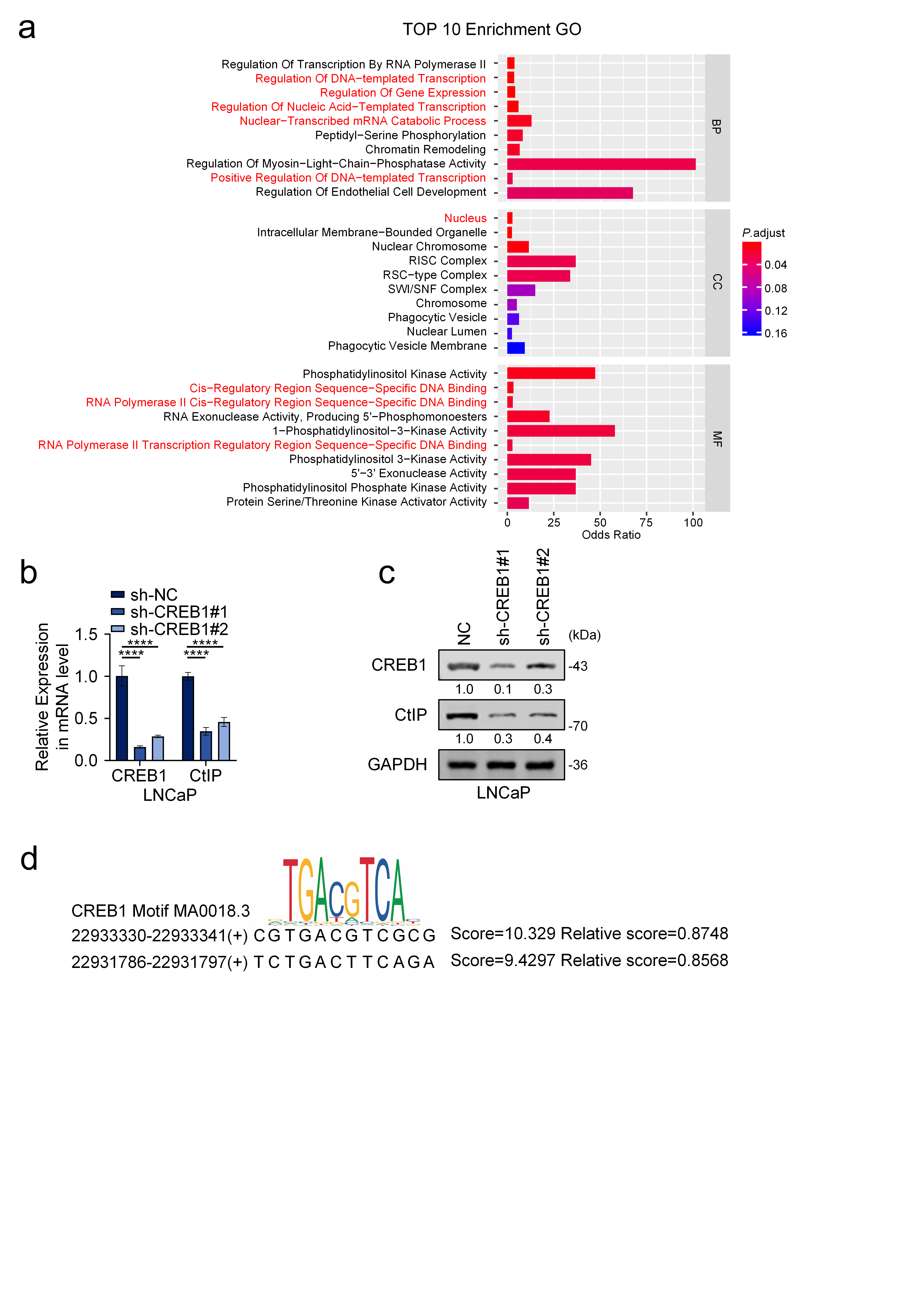


**Supplementary Figure 5**

(a) Gene Ontology (GO) enrichment analysis was performed via the Enrichr database using a gene set comprising the top 100 genes exhibiting the highest co-expression with CREB1 (identified by expanding its functionally associated gene set via the ARCHS4 RNA-seq gene-gene co-expression matrix).

(b, c) RT-qPCR (b) and Western blot (c) analysis showed the changes in CtIP expression with/without knockdown CREB1 in LNCaP cells. **** *P* < 0.0001, unpaired t-test.

(d) Lists of potential binding sequences of CREB1 in the CtIP promoter region.


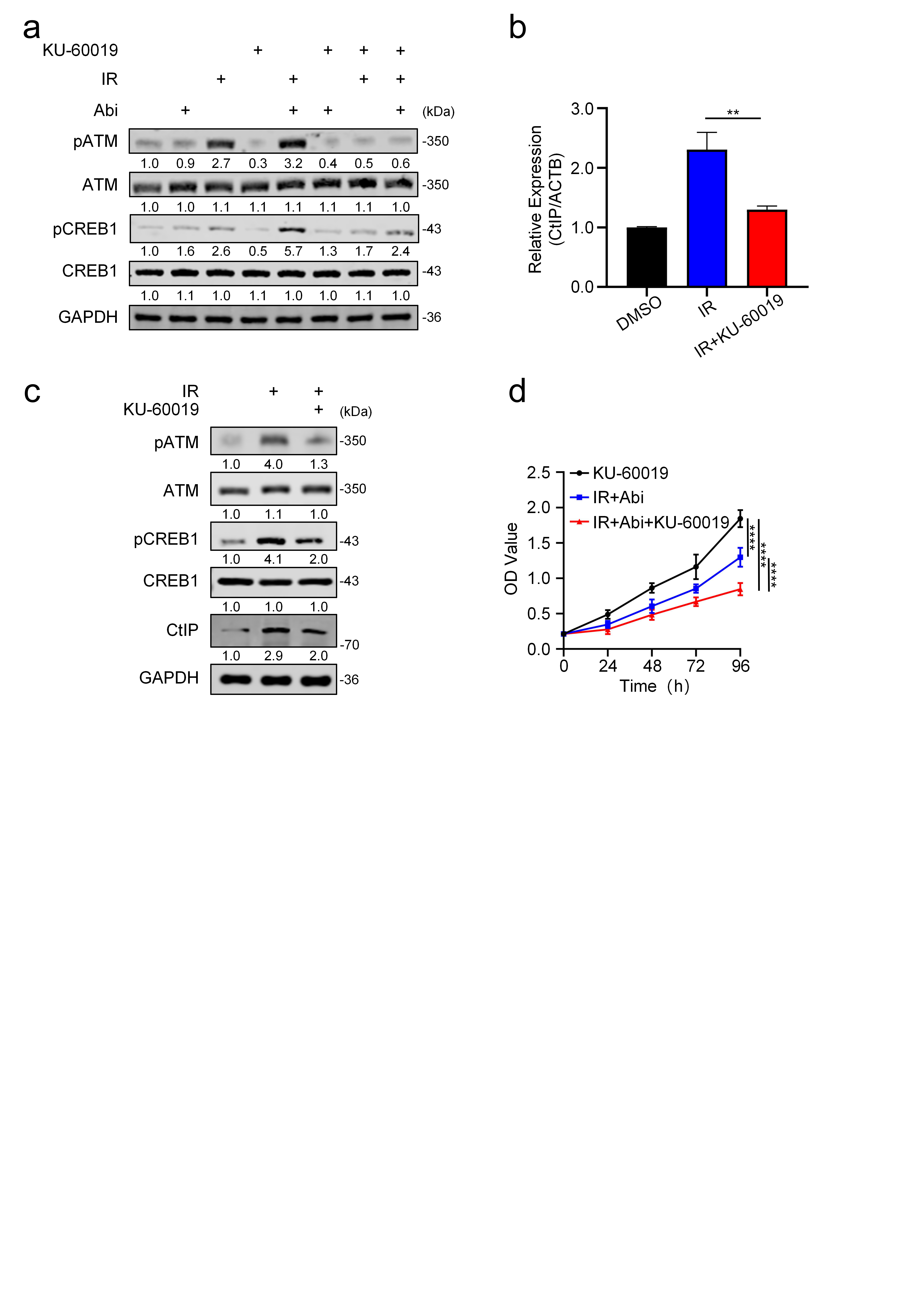


**Supplementary Figure 6**

1. Western blot analysis of the indicated proteins was performed in C4-2B cells following exposure to IR (2 Gy) and a 48-hour incubation, with or without pretreatment with Abi (10 μM, 48 hours prior to IR) and/or KU-60019 (2 μM, 0.5 hours prior to IR).

(b) RT-qPCR analysis showed the changes in CtIP mRNA expression was performed in C4-2B cells following exposure to IR (2 Gy) and a 24-hour incubation, with or without pretreatment with KU-60019 (2 μM, 0.5 hours prior to IR). ** *P* < 0.01, ANOVA.

(c) Western blot analysis of the indicated proteins was performed in C4-2B cells following exposure to IR (2 Gy) and a 48-hour incubation, with or without pretreatment with KU-60019 (2 μM, 0.5 hours prior to IR).

(d) CCK8 OD value (measured every 24 hours for 96 hours) and statistical analysis were assessed in C4-2B cells under three treatment conditions: KU-60019 (2 μM) alone; IR(2 Gy) combined with Abi(10 μM, added 48 hours prior to IR); and IR combined with Abi(10 μM, added 48 hours prior to IR) and KU-60019 (2 μM, added 0.5 hours prior to IR).**** *P* < 0.0001, ANOVA.

**
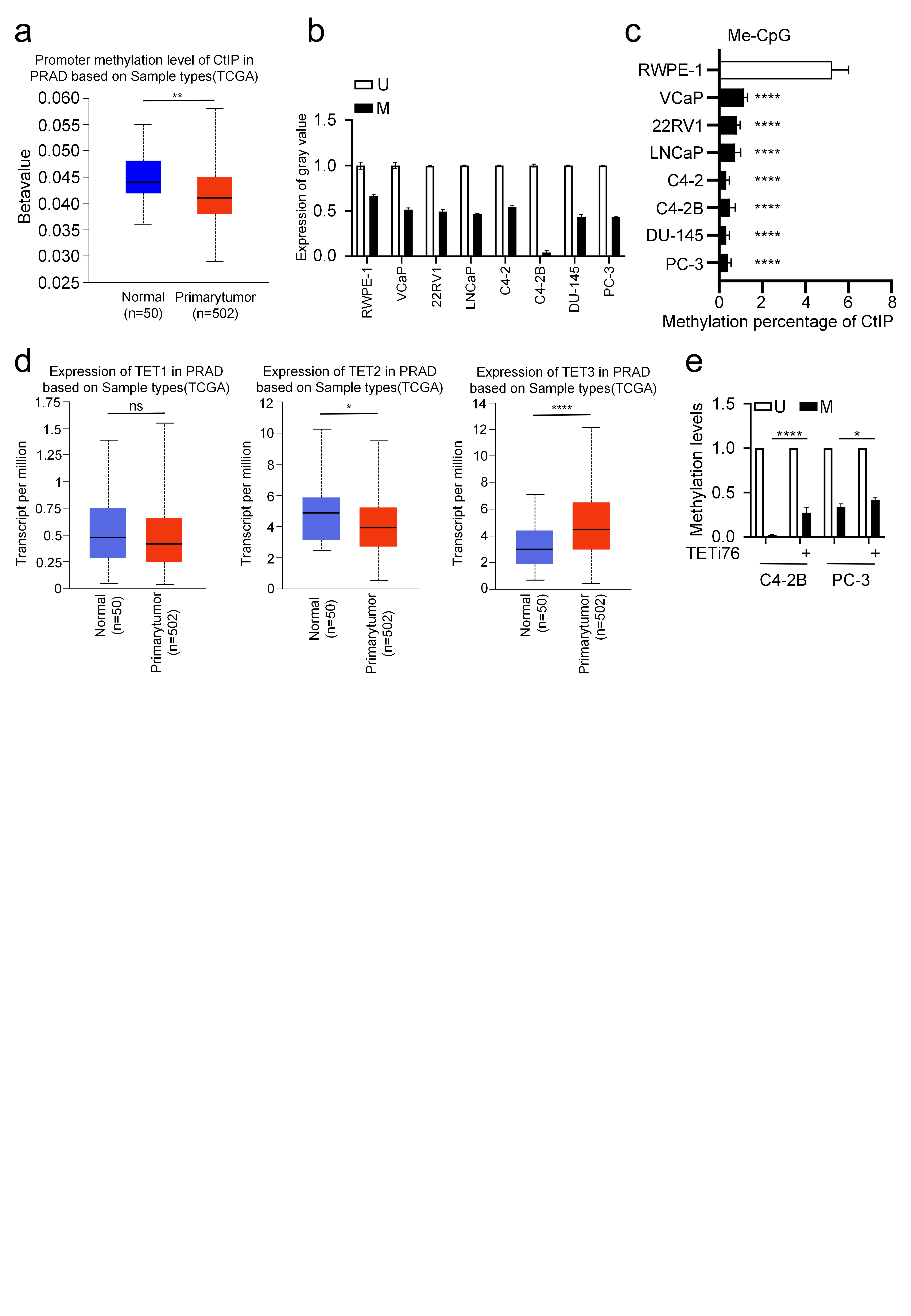
**

**Supplementary Figure 7**

1. Promoter methylation level of CtIP in PRAD. ** *P* < 0.01, unpaired t-test.
2. The bar chart shows the results of the MSP experiment. One representative experiment of 3 independent experiments is shown.

(c) The bar chart shows the results of the BSP experiment. One representative experiment of 3 independent experiments is shown. *****P* < 0.0001, unpaired t-test.

(d) Expression of TET1, TET2 and TET3 in normal prostate tissue and primary prostate cancer tissues. The data comes from the TCGA database. n.s., no significance, * *P* < 0.05, *****P* < 0.0001, unpaired t-test.

(e) The bar chart shows the results of the MSP experiment. One representative experiment of 3 independent experiments is shown. * *P* < 0.05, **** *P* < 0.0001, ANOVA.


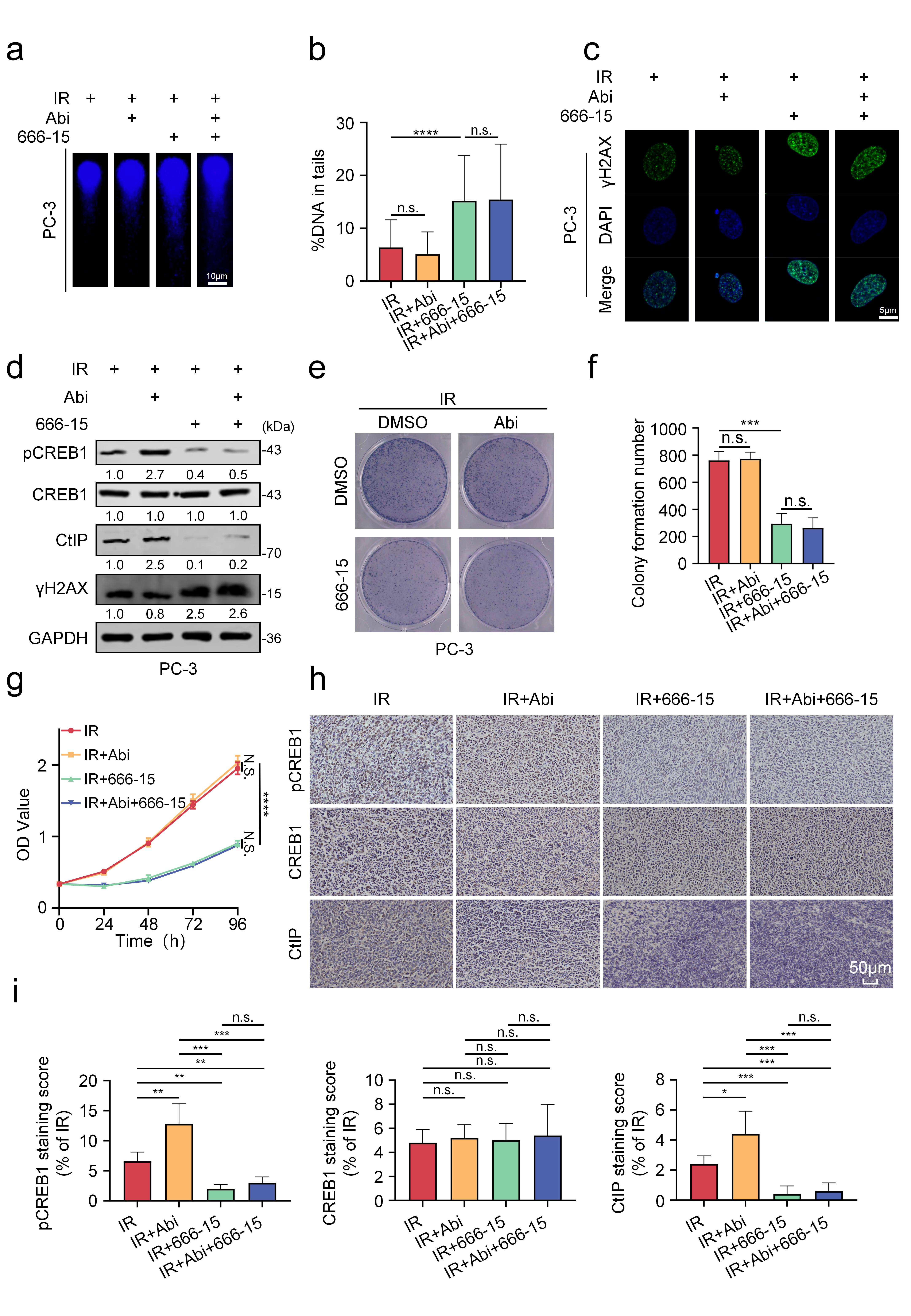


**Supplementary Figure 8**

(a, b) Comet assay(a) and statistical analysis(b) were assessed in PC-3 cells following a 48-hour incubation after treatment. Treatment conditions included: IR (2 Gy); IR combined with Abi (10 μM, added 48 hours prior to IR); IR combined with 666-15(0.5 μM, added 0.5 hours prior to IR); IR combined with Abi and 666-15. DNA damage quantified via % DNA in tails. Each data point represents at least 50 cells counted. Scale bar, 10 μm. n.s., no significance, *****P* < 0.0001, ANOVA.

1. Representative images of γH2AX foci in PC-3 cells following a 48-hour incubation after treatment. Treatment conditions included: IR (2 Gy); IR combined with Abi (10 μM, added 48 hours prior to IR); IR combined with 666-15(0.5 μM, added 0.5 hours prior to IR); IR combined with Abi and 666-15. Scale bar, 5 μm.
2. Western blot analysis of the indicated proteins was performed in PC-3 cells following a 48-hour incubation after treatment. Treatment conditions included: IR (2 Gy); IR combined with Abi (10 μM, added 48 hours prior to IR); IR combined with 666-15(0.5 μM, added 0.5 hours prior to IR); IR combined with Abi and 666-15.

(e, f) Representative images(e) and quantitative analysis(f) of colony formation assays in PC-3 cells following a 14-day incubation after treatment. Treatment conditions included: IR (2 Gy); IR combined with Abi (10 μM, added 48 hours prior to IR); IR combined with 666-15(0.5 μM, added 0.5 hours prior to IR); IR combined with Abi and 666-15. n.s., no significance, *** *P* < 0.001, ANOVA.

(g) CCK8 OD value (measured every 24 hours for 96 hours) and statistical analysis were assessed in PC-3 cells under three treatment conditions: IR (2 Gy); IR combined with Abi (10 μM, added 48 hours prior to IR); IR combined with 666-15(0.5 μM, added 0.5 hours prior to IR); IR combined with Abi and 666-15. n.s., no significance, **** *P* < 0.0001, ANOVA.

(h) IHC analysis of pCREB1, CREB1, and CtIP expression in xenografts across treatment groups. Scale bar, 50 μm

(i) IHC-based statistical analysis of pCREB1, CREB1, and CtIP expression in xenograft tissues across different treatment groups. n.s., no significance, * *P* < 0.05, ** *P* < 0.01, *** *P* < 0.001, ANOVA.


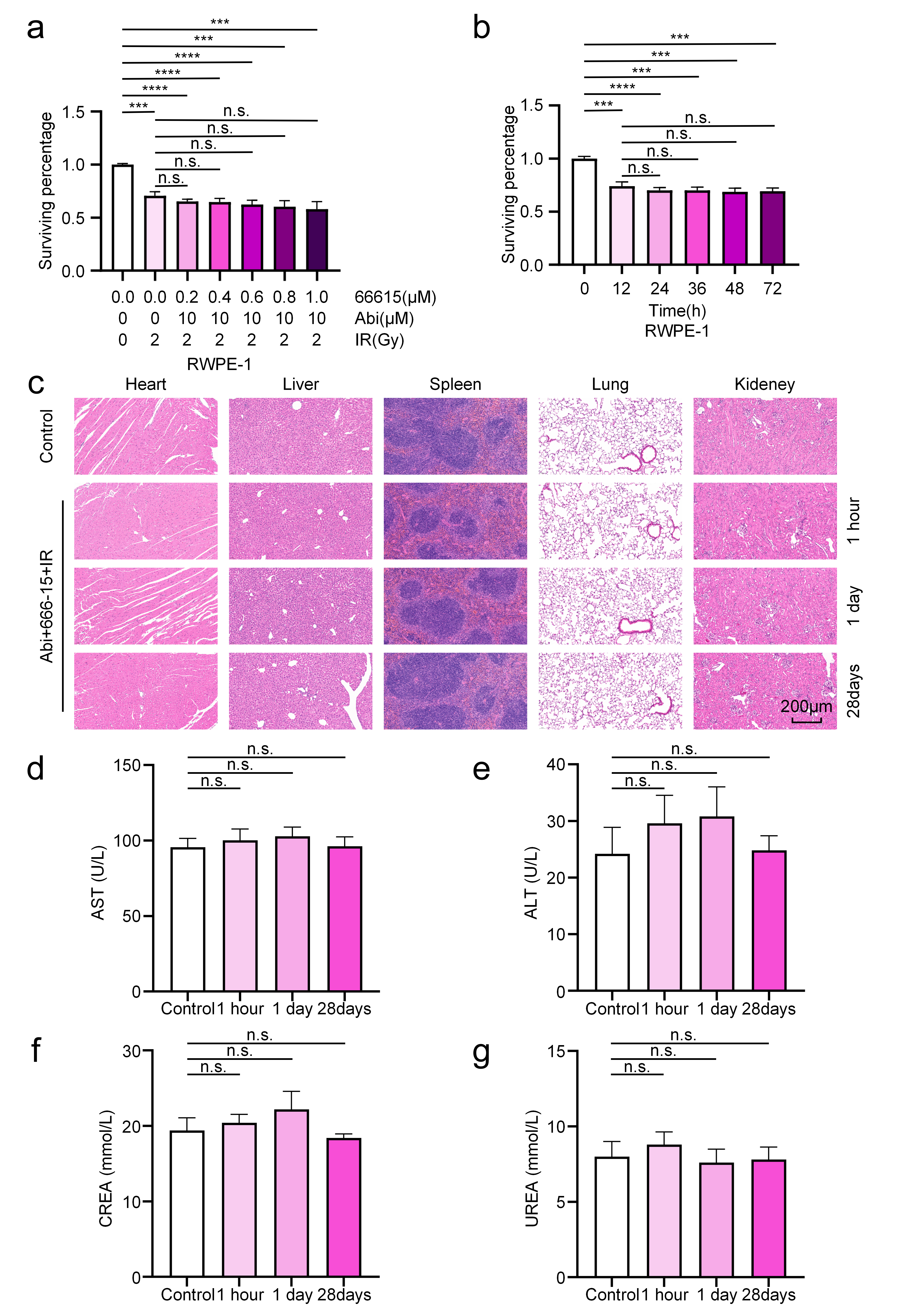


**Supplementary Figure 9**

(a) Cell viability of normal prostate epithelial cells RWPE-1 was assessed after 48 hours of treatment under different conditions. The treatment groups included: a vehicle control (equivalent dose of DMSO); a radiation control: IR (2 Gy); and experimental groups: different concentrations of 666‑15 (0.2, 0.4, 0.6, 0.8, 1.0 μM, added 0.5 h before IR) and Abi (10 μM, added 48 h before IR) and IR (2 Gy). n.s., no significance, *** *P* < 0.001, **** *P* < 0.0001, unpaired t-test.

(b) Cell viability of RWPE-1 cells was also measured at different time points (0, 12, 24, 36, 72, 96 h) after treatment in the vehicle control (equivalent dose of DMSO) and the experimental group: 666‑15(0.5 μM, added 0.5 h before IR) and Abi (10 μM, added 48 h before IR) and IR (2 Gy). n.s., no significance, *** *P* < 0.001, **** *P* < 0.0001, unpaired t-test.

(c) H&E staining images of the major organs (heart, liver, spleen, lung, and kidney) collected from the mice after treated with IR(8Gy) combined with 666-15(10 mg/kg, i.p., 5 days a week) and Abi (60 mg/kg, i.p., twice weekly) (Abiraterone and 666-15 were administered 4 hours prior to IR.) for 1 h, 1day, and 14 days. Scale bar, 200 μm.

(d-g) Blood biochemical analysis of mice at 1 h, 1day, and 14 days after treated with IR(8Gy) combined with 666-15(10 mg/kg, i.p., 5 days a week) and Abi (60 mg/kg, i.p., twice weekly) (Abiraterone and 666-15 were administered 4 hours prior to IR.). n.s., no significance, ANOVA.


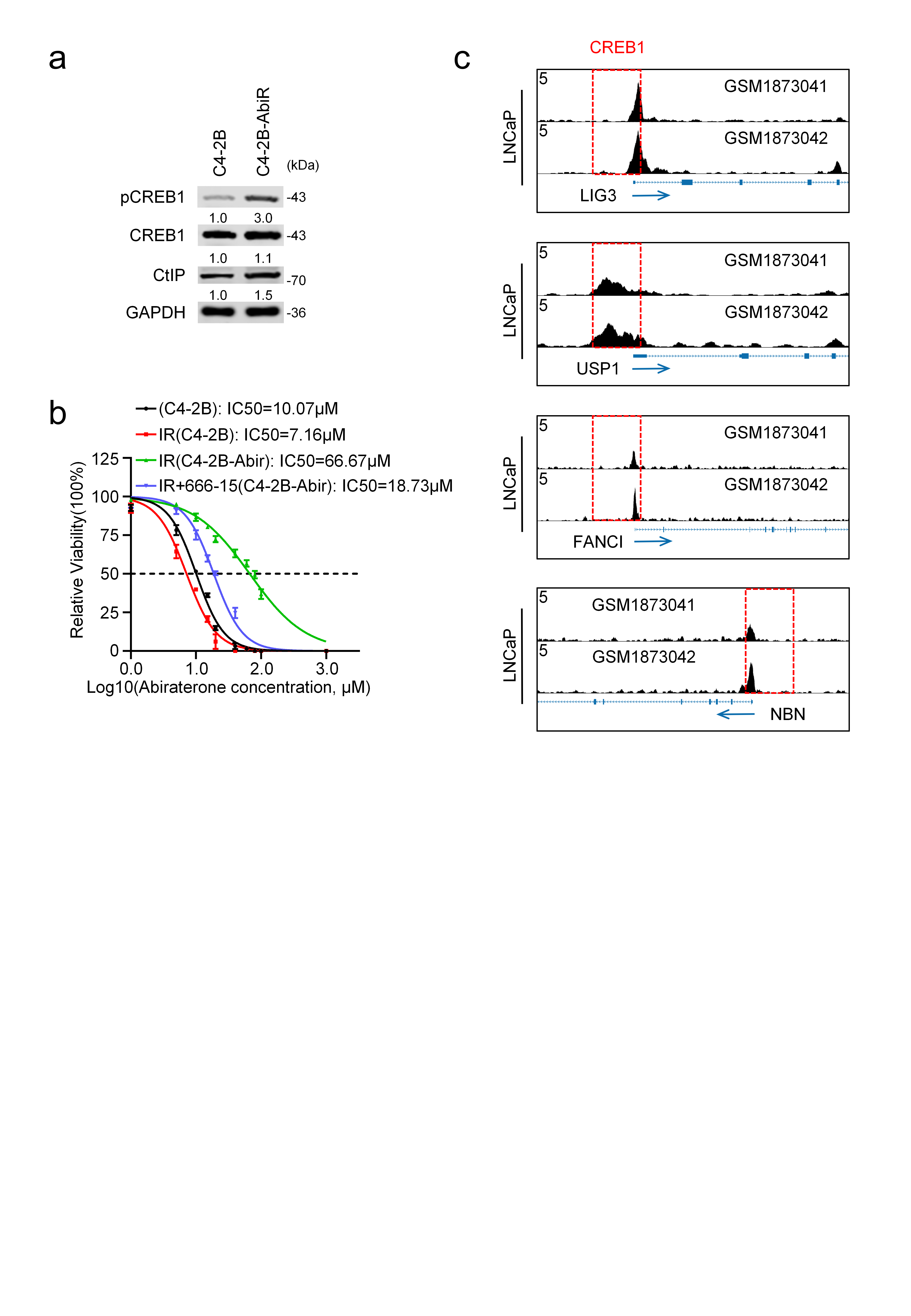


**Supplementary Figure 10**

(a) Western blot analysis of the indicated proteins was performed in C4-2B and C4-2B-AbiR cells

(b) The IC50 values were calculated to compare abiraterone sensitivity, based on the optical density measured by CCK-8 assay at 72 hours post-treatment. The experimental design included the following groups: Abi treatment alone in C4-2B cells; IR (2 Gy) combined with Abi (administered 48 hours prior to irradiation) in both C4-2B and C4-2B-AbiR cells; and a triple combination of IR (2 Gy), Abi (added 48 hours before irradiation), and 666-15 (0.5 μM, added 0.5 hours before irradiation) in C4-2B-AbiR cells.

(c) The ChIP-seq results showed the binding of CREB1 to the FANCI, USP1, NBN and LIG3 promoter region (red dotted line aera).

**Supplementary Table S1. Sequence information of shRNAs**

| **Gene** | **Sequence (5’-3’)** |
| --- | --- |
| sh-control | 5′- -3′CAACAAGATGAAGAGCACCAA |
| sh-*CREB1* -#1 | 5′- -3′GCTCGATAAATCTAACAGTTA |
| sh-*CREB1* -#2 | 5′- -3′CAGTGGATAGTGTAACTGATT |
| sh-*AR*-#1 | 5′- -3′CTGAAGAAACTTGGTAATCTT |
| sh-*AR*-#2 | 5′- -3′CGTGGACTTTCCGGAAATGAT |

**Supplementary Table S2. Sequence information of primers for RT-qPCR**

| **Species** | **Gene** | **Forward (5’-3’)** | **Reverse (5’-3’)** |
| --- | --- | --- | --- |
| Human | *ACTB* | CTCCATCCTGGCCTCGCTGT | GCTGTCACCTTCACCGTTCC |
| Human | *CtIP* | GGCTTATGTGATCGCTGTGC | ATGTGCTTTGGCCATTGGAG |
| Human | *AR* | TGCCAAGAAGTCCAAGAACCACATC | TGTCGGAGGTCCTGGGTCAAC |
| Human | *KLK3* | CCACTACTGGCCATCCCAGA | GCCAGGACTCCTCAAACAGC |
| Human | *CCNH* | GCGATTGCTGGGTGTGGTGTC | GTTGAGGTATGCCATGCCGTCTG |
| Human | *FANCI* | TGGACTGACAAATGCTGGAGAACTG | GTTACCGCTGGGGCTGTTGC |
| Human | *LIG3* | TGAGCATAAGCGTTCACTGACCAAG | GAGTCTGCGTTGCCTCAGTTGTC |
| Human | *MCM2* | CCCAAAGAAGCTGTGGAGGAGAATG | GCCTAGTTCCCAACGCTGCATAG |
| Human | *NBN* | ACCTTGCCTGCTTCAGCTAGAAAAG | TCCCTTAGTGCAGCATTTAGCCTTG |
| Human | *PLOA2* | AGGAGCTAGAGACATTGTTTCCA | CTCGCTTCTGAGAACCCTTTG |
| Human | *PLOE2* | ATTTACTCCTCCGGTGATAGGTT | GCATCTCCGATTTTGGTTGTACT |
| Human | *TDP1* | CCTTTATTTGGGACGCTTGTTTCTTC | GATTGGCTTCTTCCTGAACTCTGG |
| Human | *USP1* | CCAGAACCATTGAATGAGGAGGAAG | TCCACCAACTCTAATTGACACTTCTTC |
| Human | *XRCC5* | GCACTGACAATCCCCTTTCTG | TCAATGTCCTCCAGCAAATCAAA |
| Human | *CREB1* | TCATCTGCTCCCACCGTAACTC | TTGGACTTGTGGAGACTGAATAACTG |
| Human | *BRD4* | CTCCCCGCTTATGATACATTCC | GTTTCTTAGGCTGGACGTTTTG |
| Human | *CREM* | TGATGGCACACAGCAGTTCTTTG | GGATCTGGTAAGTTGGCATGTCAC |
| Human | *SPI1* | CAACCGCAAGAAGATGACCTACC | GCACTTCGCCGCTGAACTG |
| ChIP-qPCR | | | |
| Human | *CtIP* | GATGGGCAGCTGGAGGAA | AAATCCTCGGTGGGAAAGCC |

**Supplementary Table S3. Sequence information of primers for MSP**

| **Species** | **Gene** | **Status** | **Forward (5’-3’)** | **Reverse (5’-3’)** |
| --- | --- | --- | --- | --- |
| Human | *CtIP* | M | AATTTAGAAATGTTGTGGCGGTC | CCTTCCTCCAACTACCCATCG |
| Human | *CtIP* | U | GTAATTTAGAAATGTTGTGGTGGTTG | CCTTCCTCCAACTACCCATCA |

**Supplementary Table S4. Antibodies and other reagents and resources**

| **REAGENT or RESOURCE** | **SOURCE** | **IDENTIFIER** |
| --- | --- | --- |
| **Antibodies** | | |
| Rabbit polyclonal anti-GAPDH  (WB: 1/1000) | Abcam | Cat# ab9485 |
| Rabbit polyclonal anti-CtIP  (WB: 1 µg/ml) | Abcam | Cat# ab117722 |
| Rabbit monoclonal anti-CREB1  (WB: 1/1000) | Cell Signaling Technology | Cat# 9197 |
| Rabbit monoclonal anti-Phospho-  CREB1 (Ser133)  (WB: 1/1000) | Cell Signaling Technology | Cat# 9198 |
| Rabbit monoclonal anti-AR  (WB: 1/2000) | Abcam | Cat# ab133273 |
| Rabbit monoclonal anti-Phospho-  Histone H2AX-S139  (WB: 1/5000) | ABclonal | Cat# AP0687 |
| Rabbit monoclonal anti-BRD4  (WB:1/1000) | Cell Signaling Technology | Cat# 13440 |
| Rabbit polyclonal anti- CREM  (WB:1/200) | ABclonal | Cat# A5624 |
| Rabbit polyclonal anti-SPI1  (WB: 1/800) | ABclonal | Cat# A24910 |
| Rabbit monoclonal anti-ATM  (WB: 1/1000) | Cell Signaling Technology | Cat# 2873 |
| Rabbit monoclonal anti-Phospho-  ATM (Ser1981)  (WB: 1/1000) | Cell Signaling Technology | Cat# 5883 |
| Rabbit monoclonal anti-Phospho-  CREB1 (Ser133)  (ChIP-qPCR) | Cell Signaling Technology | Cat# 9198 |
| Rabbit monoclonal anti-PKA  (WB: 1/1000) | Cell Signaling Technology | Cat# 4782 |
| Rabbit monoclonal anti-Phospho-  PKA (Thr197)  (WB: 1/1000) | Cell Signaling Technology | Cat# 4781 |
| Rabbit monoclonal anti-Phospho-  (Ser/Thr) Phe  (WB: 1/100) | Abcam | Cat# ab17464 |
|  | | |
| **Chemicals, Peptides, and**  **Recombinant Proteins** |  |  |
| Abiraterone | MedChemExpress | Cat#HY-70013 |
| 666-15 | MedChemExpress | Cat#HY-101120 |
| Forskolin | MedChemExpress | Cat#HY-15371 |
| KU-60019 | MedChemExpress | Cat#HY-12061 |
| TETi76 | MedChemExpress | Cat#HY-402410 |
| Decitabine | MedChemExpress | Cat#HY-A0004 |
| H89 | MedChemExpress | Cat#HY-15979 |

**Supplementary Table S5. The CREB1/CtIP top 100 co-expressed gene set (comprising genes with the highest co-expression and functional relevance to CREB1 and CtIP).**

| **Gene** | **Gene set** |
| --- | --- |
| *RBBP8(CtIP)* | *ARHGAP11A；ASPM；ATAD2；AURKA；BRCA1；BRCA2；BRIP1；BUB1；BUB1B；CASC5；CCNA2；CCNB1；CCNB2；CCT2；CCT5；CCT6A；CDC25C；CDC6；CDCA2；CDK1；CDKN3；CENPE；CENPF；CENPI；CENPN；CENPU；CENPW；CEP55；CHEK1；CKAP2；CKAP2L；CKS2；CLSPN；CSE1L；DBF4；DDX21；DEPDC1；DEPDC1B；DIAPH3；DLGAP5；DSCC1；DTL；E2F7；ECT2；ERCC6L；EXO1；EZH2；FANCI；FBXO5；HELLS；HJURP；HMMR；KIAA1524；KIF11；KIF14；KIF15；KIF18A；KIF20A；KIF20B；KIF23；KIF4A；KPNA2；MASTL；MCM10；MELK；MND1；NCAPG；NCAPH；NDC1；NEIL3；NEK2；NPM1；NUF2；NUSAP1；PARPBP；PBK；PLK4；POLE2；POLQ；PRC1；PTTG1；RACGAP1；RAD18；RAD51AP1；RFC3；RRM1；RRM2；SGO1；SGO2；SKA3；SMC2；SMC4；STIL；TOP2A；TPX2；TRIP13；TTK；WDHD1；WDR43；ZWILCH* |
| *CREB1* | *ACAP2；AGTPBP1；ANGEL2；APAF1；ARID2；ARID4A；ARID4B；ATAD2B；ATF2；ATF7IP；ATM；BACH1；BNIP2；C21orf91；CASP8AP2；CCNT2；CENPC；CEP135；CEP85L；CHD1；CNOT6L；CRLF3；CTDSPL2；DCP2；DICER1；DNAJB14；ELF2；FAM126B；FAM76B；FOXN2；GPATCH2L；INO80D；JMJD1C；KIAA1033；KIAA1551；KLHL28；MAP3K1；MAP3K2；45723；MIER1；MORC3；MSL2；N4BP2；NCOA2；NPAT；OSBPL8；PAN3；PAPOLG；PARP11；PBRM1；PDE7A；PGGT1B；PHIP；PIAS1；PIK3C3；PIK3CA；PIKFYVE；PPP1R12A；RAB33B；RAB8B；RAPGEF6；RASA2；RC3H1；RICTOR；ROCK1；RSBN1；SENP7；SETX；SF3B1；SHOC2；SMCHD1；SOS2；SP3；SP4；SPAST；STAG2；STK4；SWT1；TET2；TRDMT1；USP15；VCPIP1；WHSC1L1；XRN1；ZBTB37；ZFP14；ZNF100；ZNF107；ZNF136；ZNF141；ZNF148；ZNF267；ZNF292；ZNF33A；ZNF430；ZNF484；ZNF624；ZNF654；ZNF708；ZNF720* |
