# Supplementary figures and images for: Targeted inhibition of the CREB1-CtIP axis enhances the efficacy of abiraterone combined with radiotherapy in prostate cancer
Source: Cell Death Dis. 2026 Mar 30;17(1):435. doi: 10.1038/s41419-026-08633-0 (PMC13158305; doi:10.1038/s41419-026-08633-0)

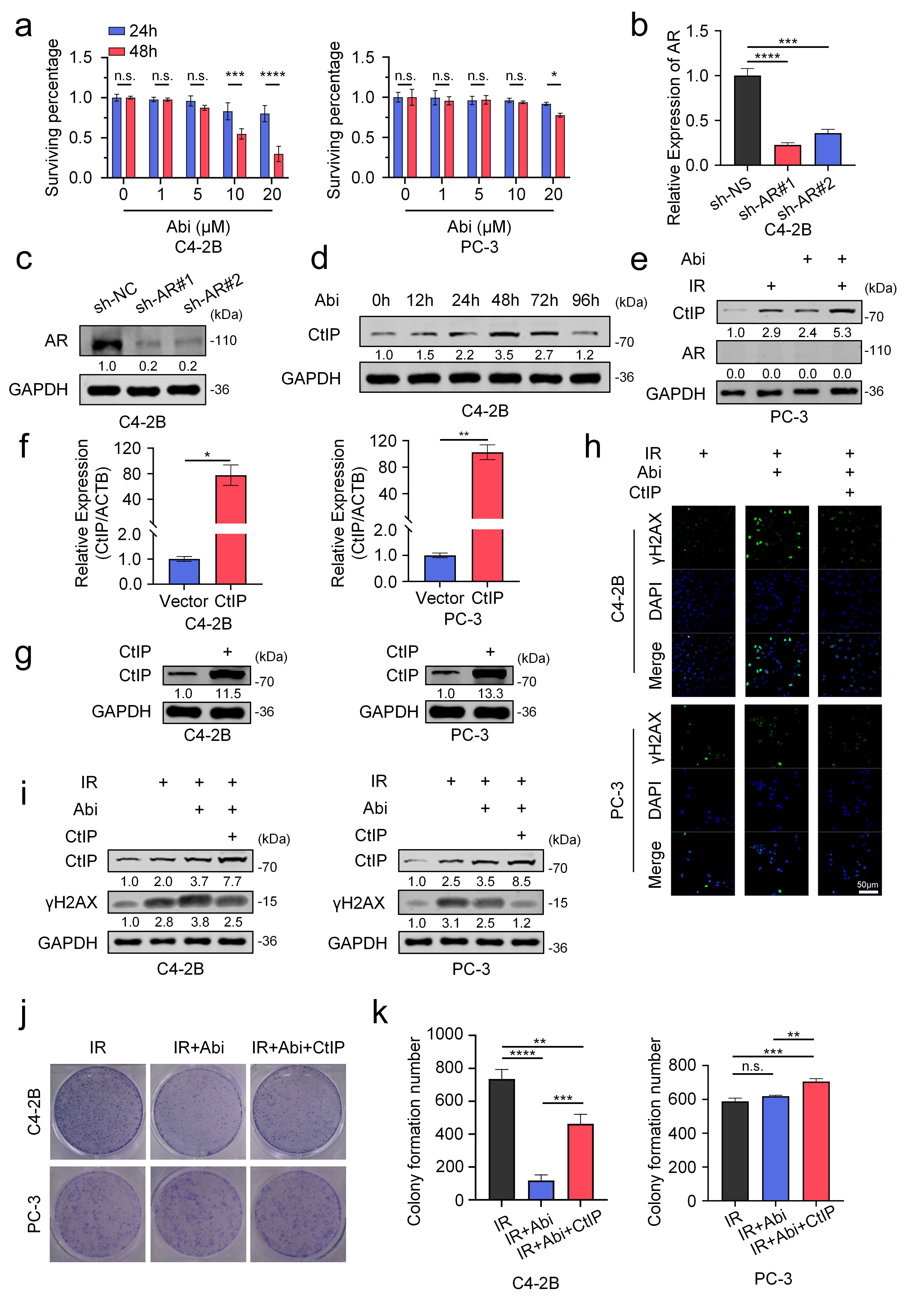

Supplement: Supplementary file 2 — Supplementary Figure 1 [file 41419_2026_8633_MOESM2_ESM.png]

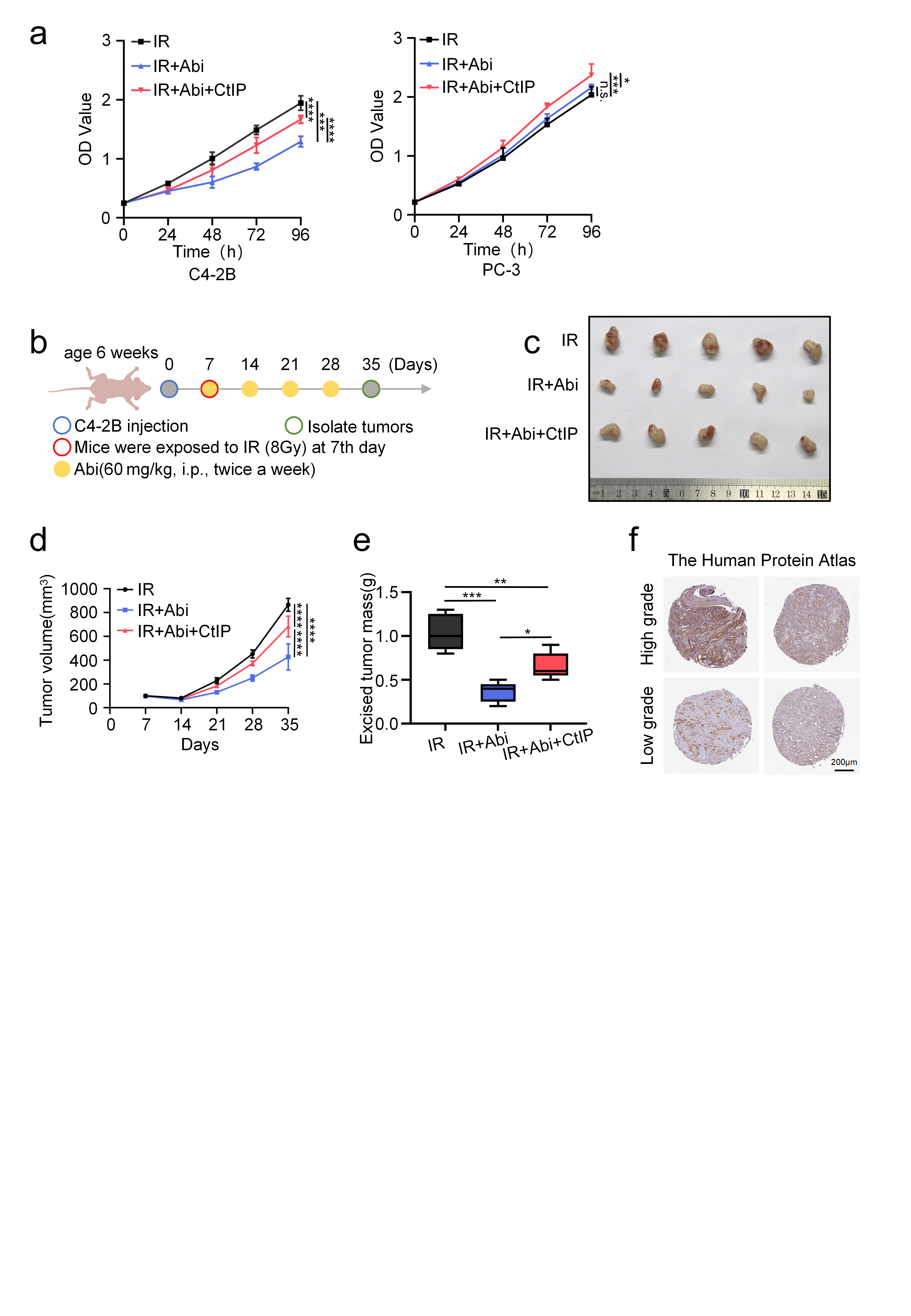

Supplement: Supplementary file 3 — Supplementary Figure 2 [file 41419_2026_8633_MOESM3_ESM.png]

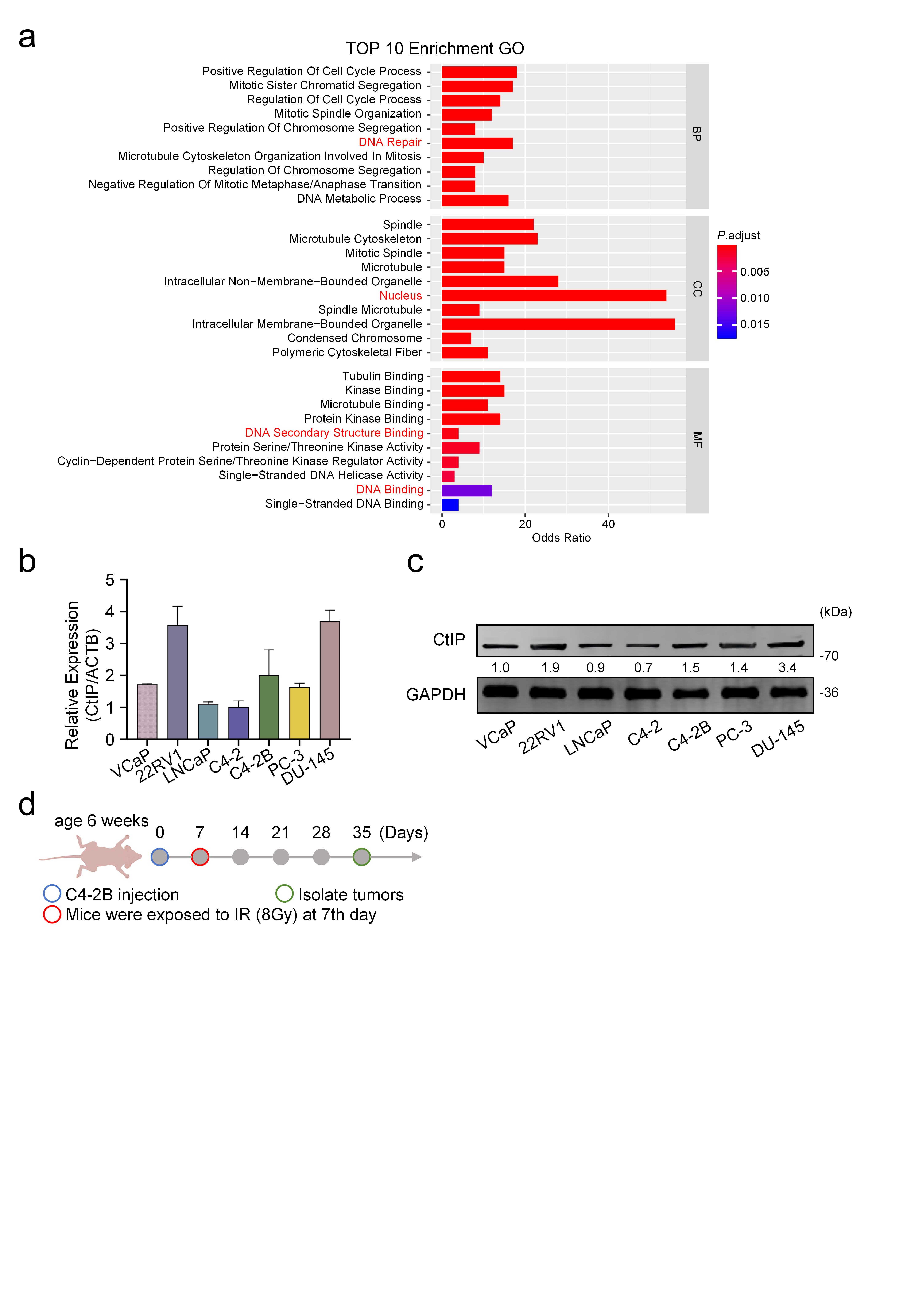

Supplement: Supplementary file 4 — Supplementary Figure 3 [file 41419_2026_8633_MOESM4_ESM.png]

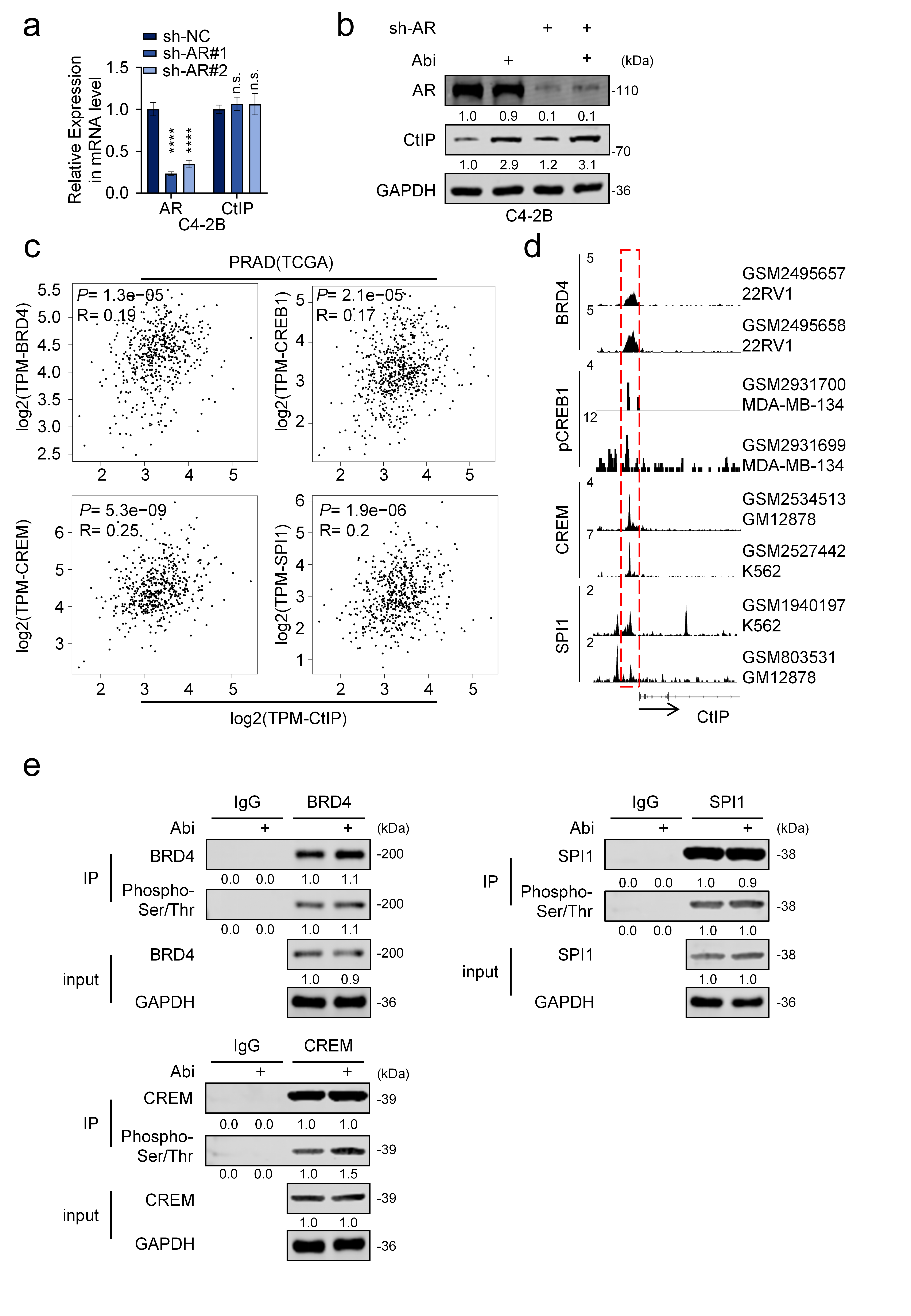

Supplement: Supplementary file 5 — Supplementary Figure 4 [file 41419_2026_8633_MOESM5_ESM.png]

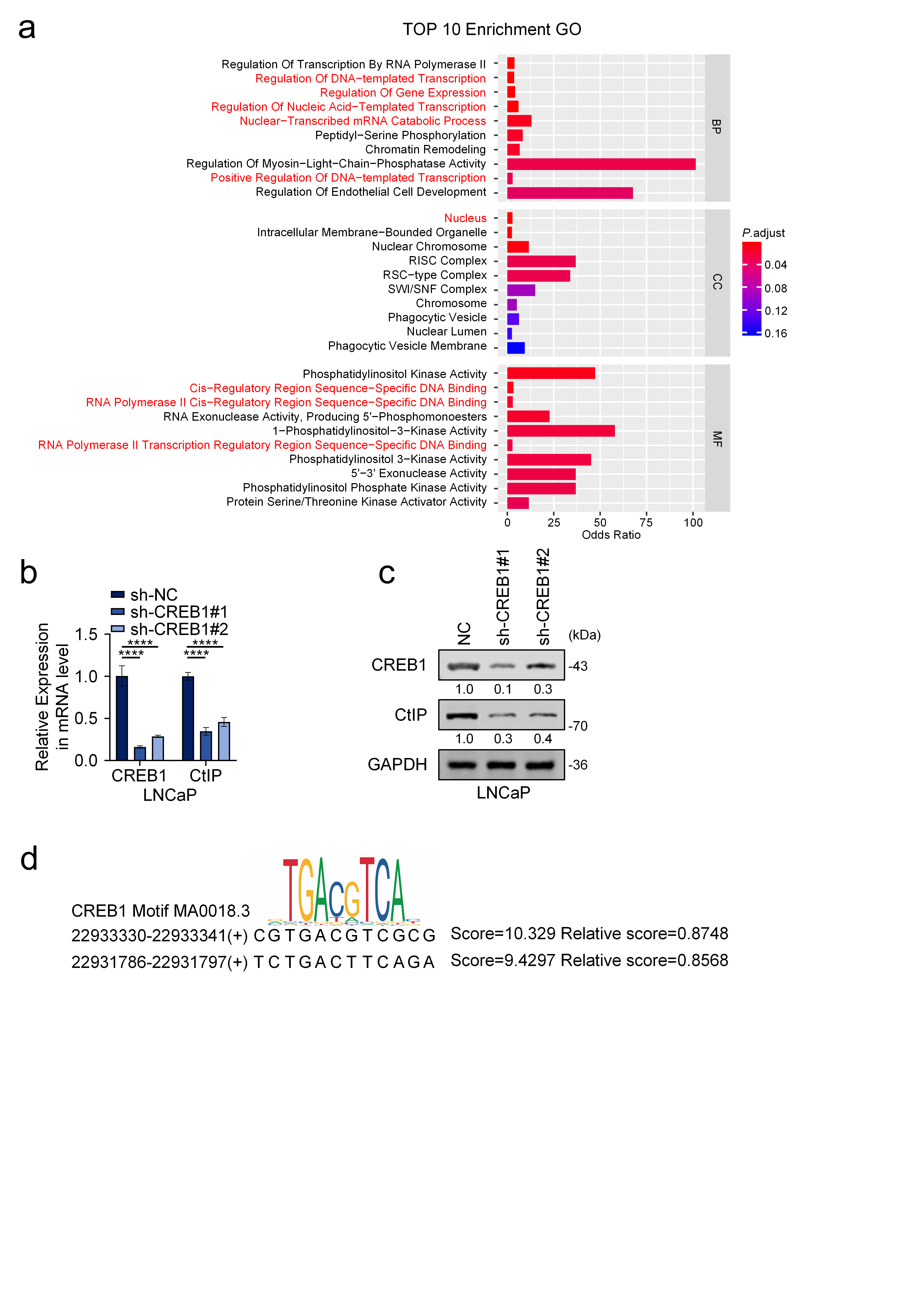

Supplement: Supplementary file 6 — Supplementary Figure 5 [file 41419_2026_8633_MOESM6_ESM.png]

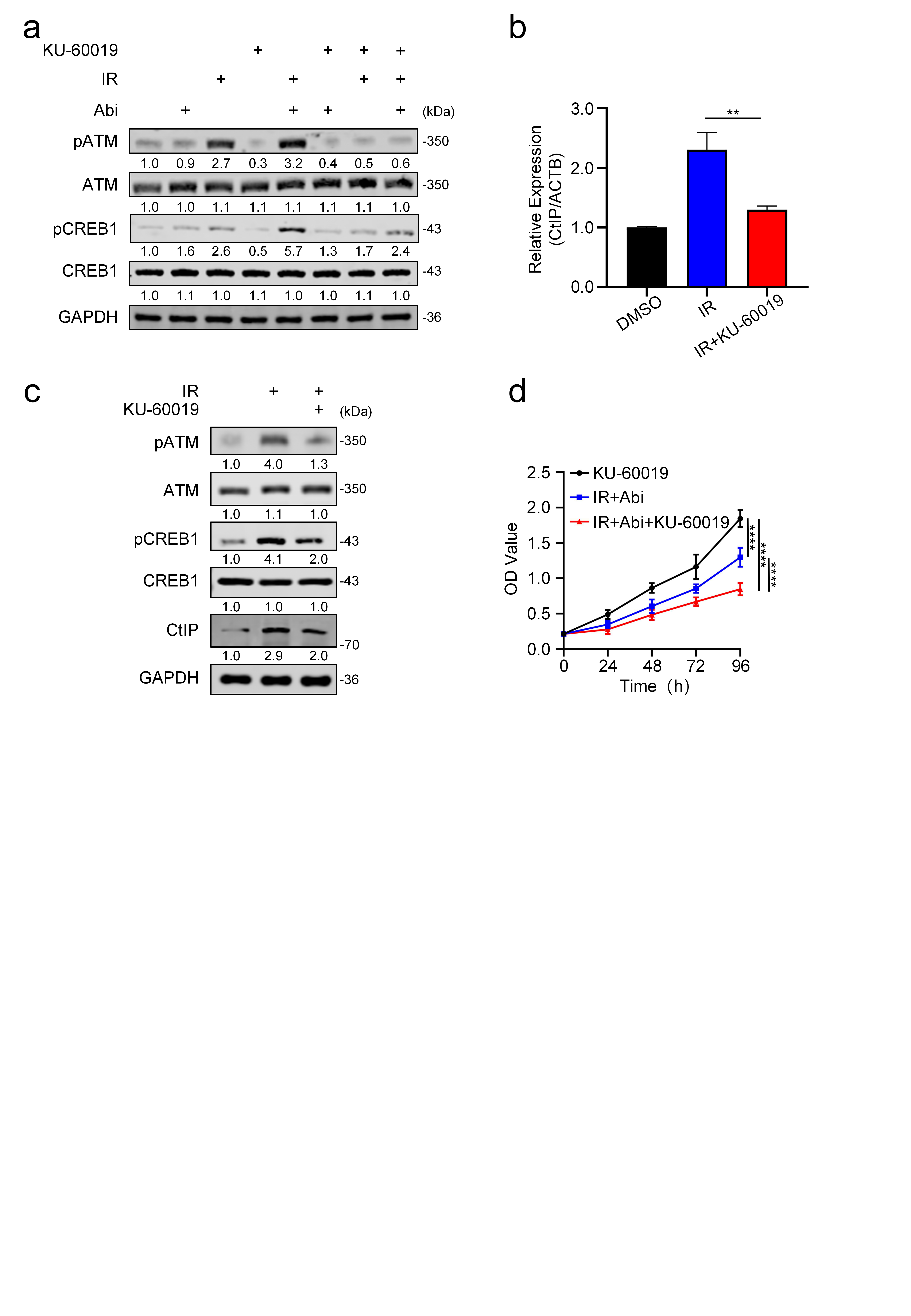

Supplement: Supplementary file 7 — Supplementary Figure 6 [file 41419_2026_8633_MOESM7_ESM.png]

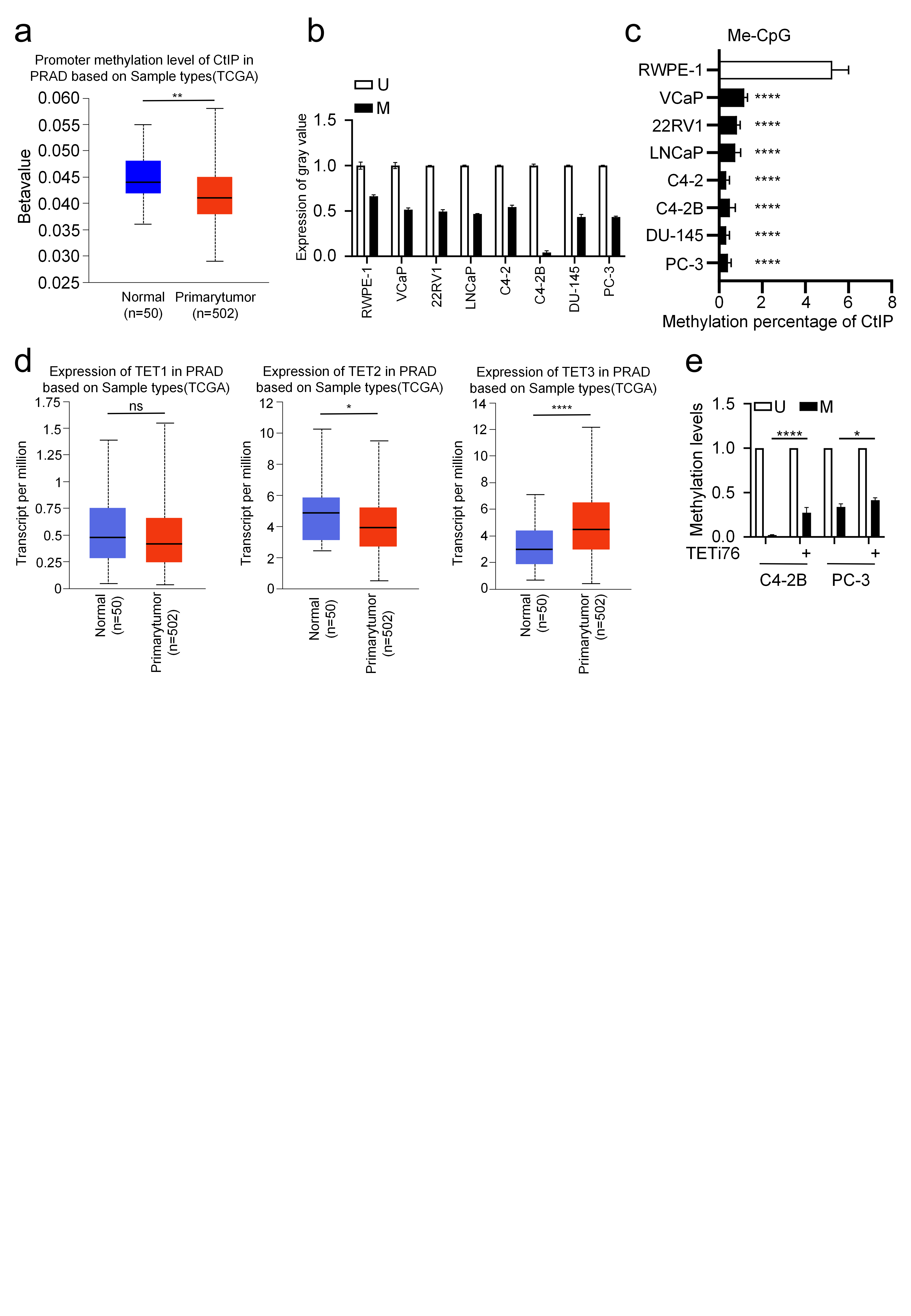

Supplement: Supplementary file 8 — Supplementary Figure 7 [file 41419_2026_8633_MOESM8_ESM.png]

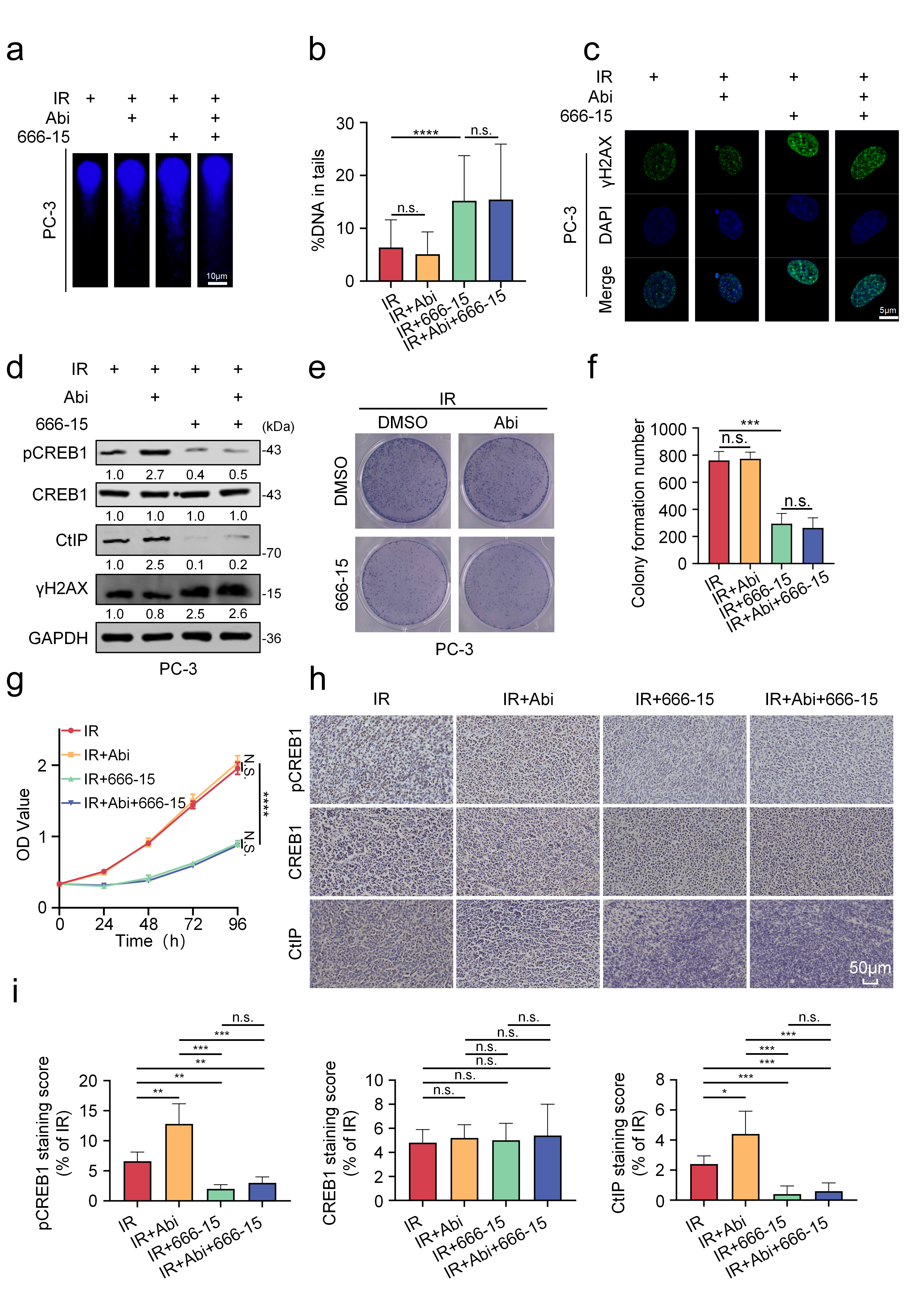

Supplement: Supplementary file 9 — Supplementary Figure 8 [file 41419_2026_8633_MOESM9_ESM.png]

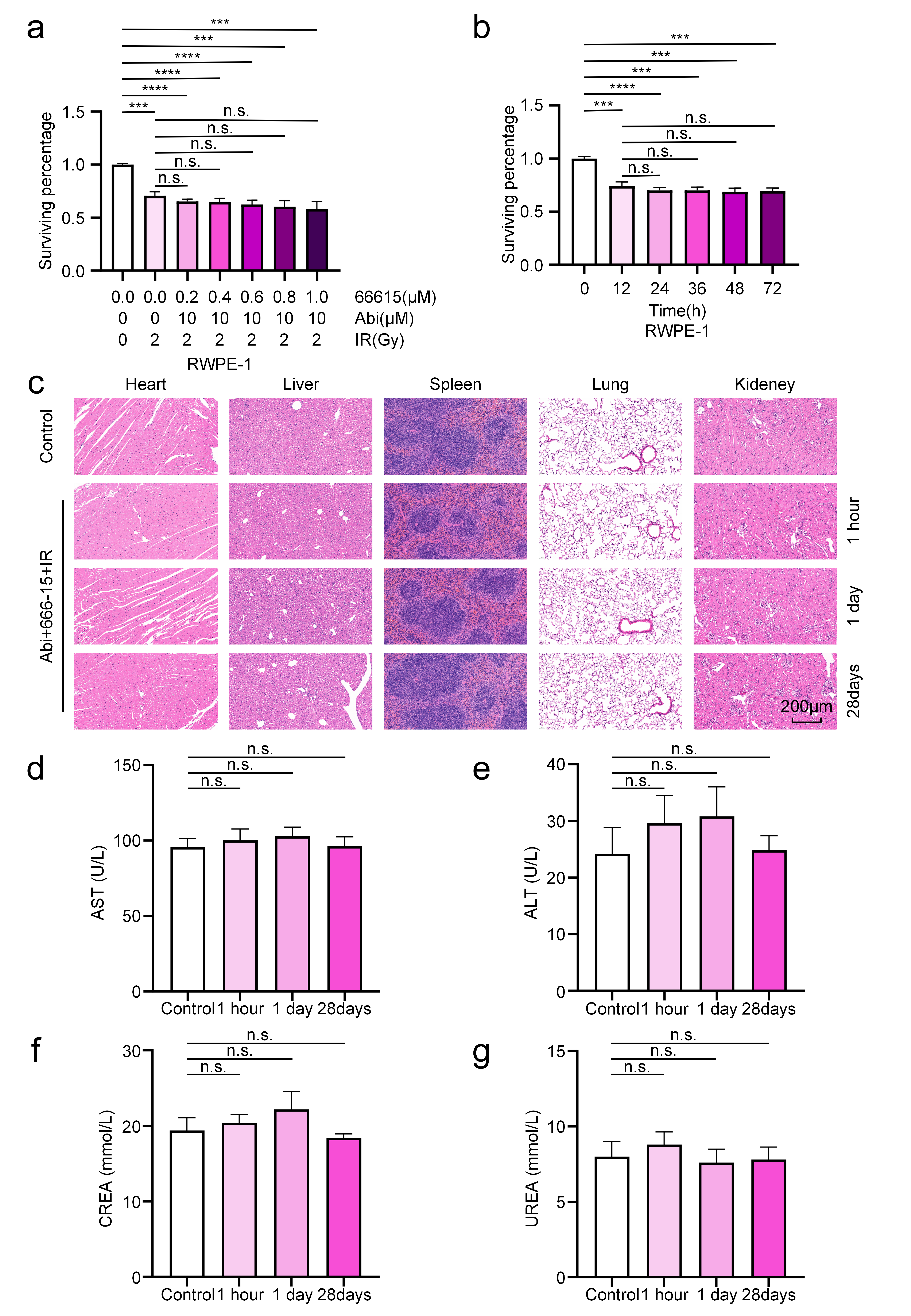

Supplement: Supplementary file 10 — Supplementary Figure 9 [file 41419_2026_8633_MOESM10_ESM.png]

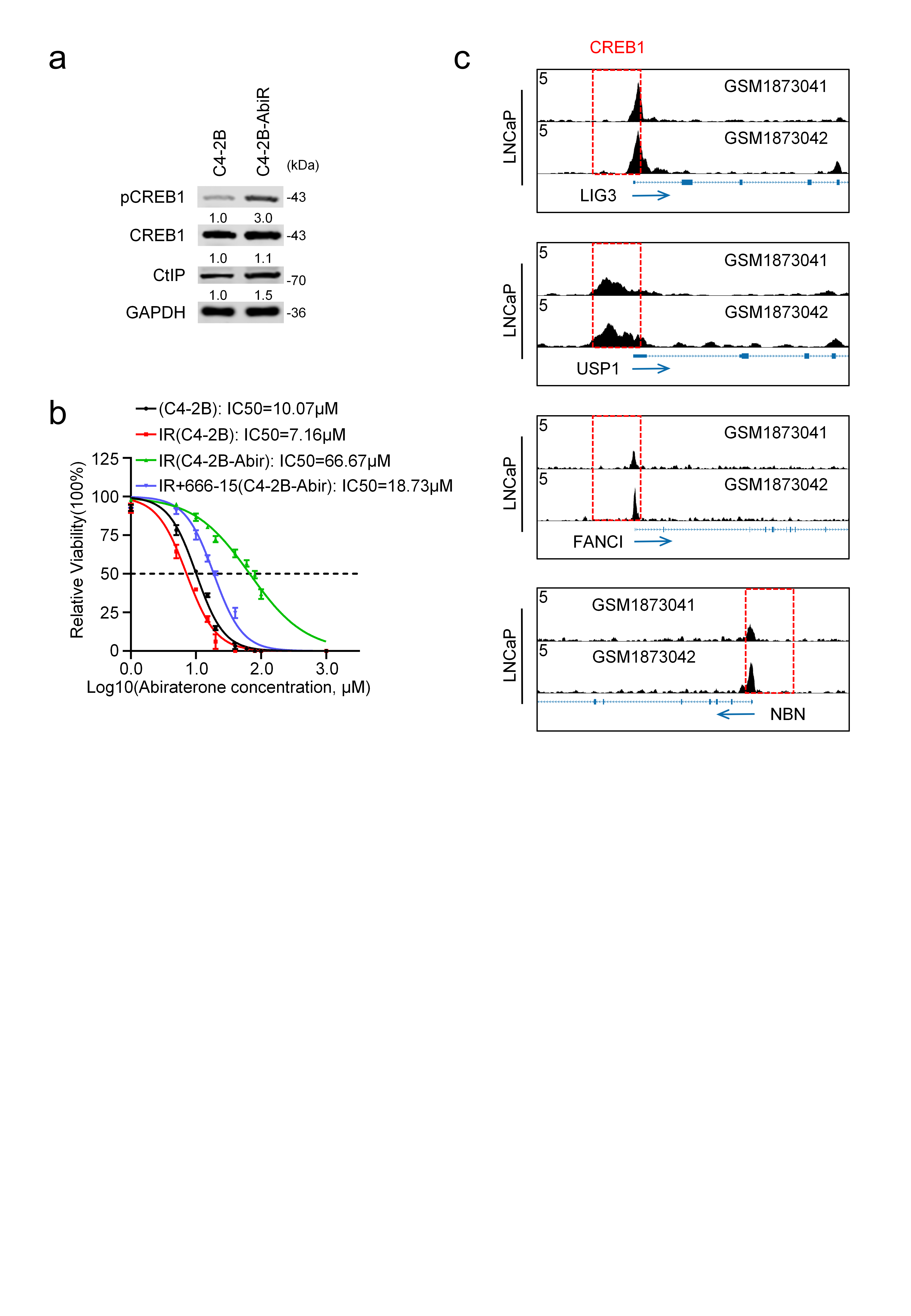

Supplement: Supplementary file 11 — Supplementary Figure 10 [file 41419_2026_8633_MOESM11_ESM.png]
