# Supplementary material for: Targeted inhibition of the CREB1-CtIP axis enhances the efficacy of abiraterone combined with radiotherapy in prostate cancer
Source: Cell Death Dis. 2026 Mar 30;17(1):435. doi: 10.1038/s41419-026-08633-0 (PMC13158305; doi:10.1038/s41419-026-08633-0)

Figure 1

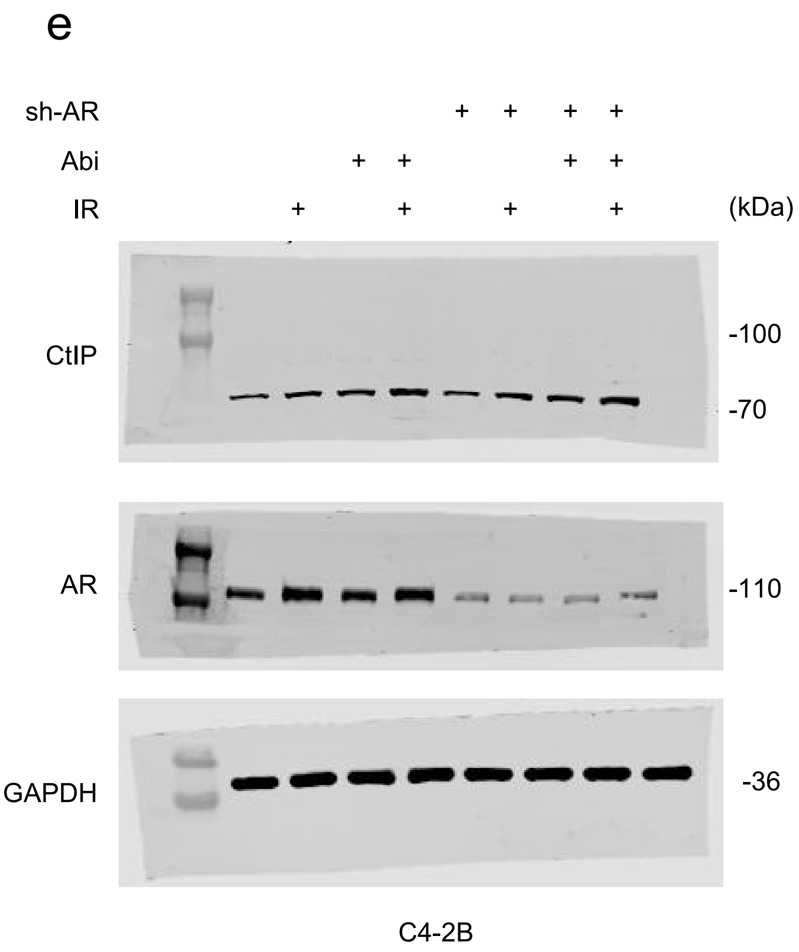

Figure 2

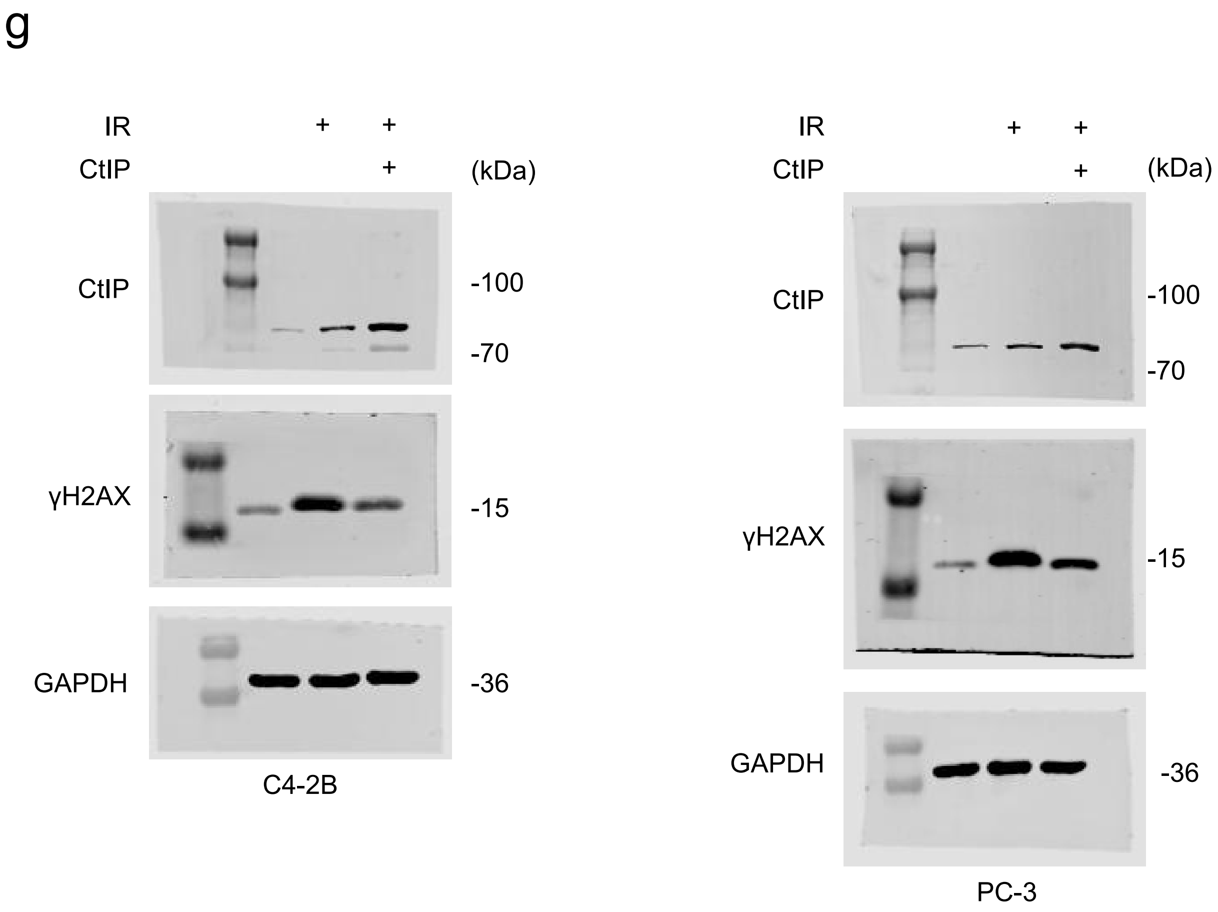
g
IR
+
+
CtIP
+
(kDa)
CtIP
-100
-70
γH2AX
-15
GAPDH
-36
C4-2B
IR
+
+
CtIP
+
(kDa)
CtIP
-100
-70
γH2AX
-15
GAPDH
-36
PC-3

Figure 3

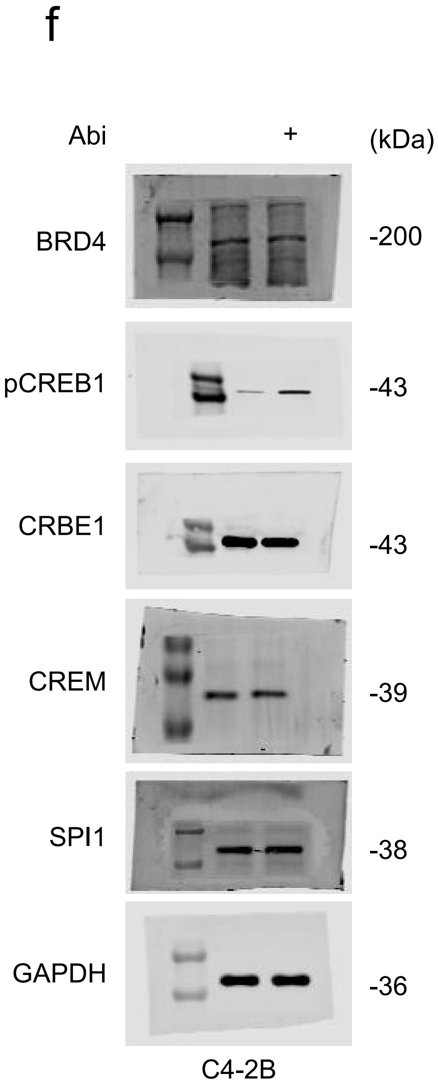
f
Abi
+
(kDa)
BRD4
-200
pCREB1
-43
CRBE1
-43
CREM
-39
SPI1
-38
GAPDH
-36
C4-2B

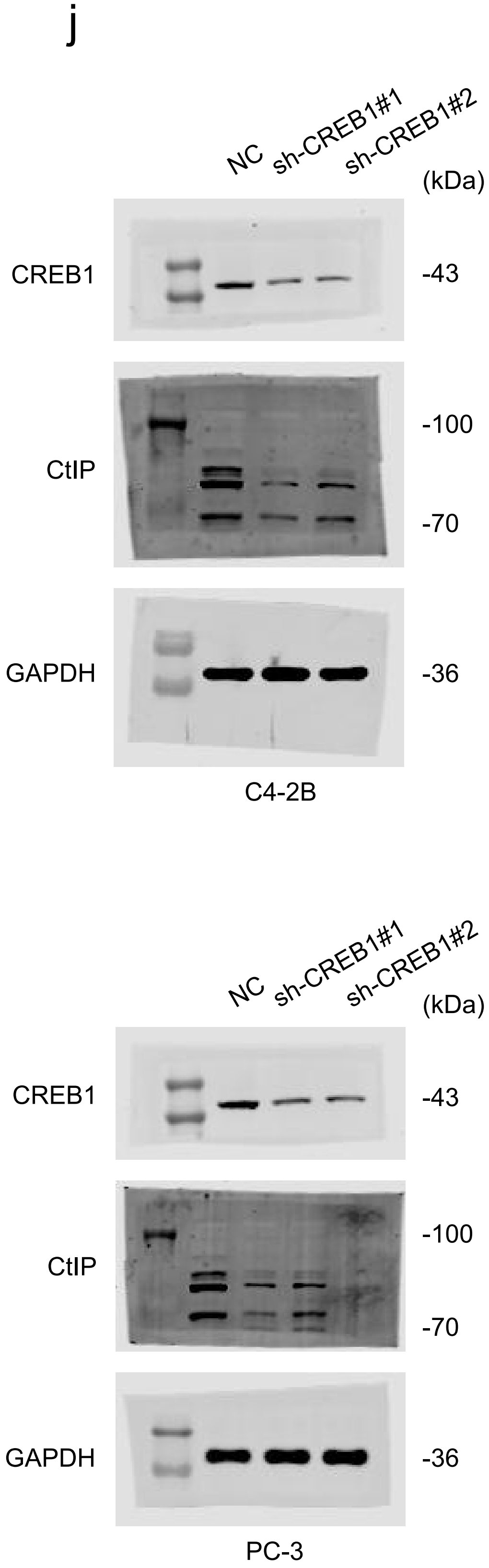
j
NC
sh-CREB1#1
sh-CREB1#2
(kDa)
CREB1
-43
CtIP
-100
-70
GAPDH
-36
C4-2B
NC
sh-CREB1#1
sh-CREB1#2
(kDa)
CREB1
-43
CtIP
-100
-70
GAPDH
-36
PC-3

Figure 4

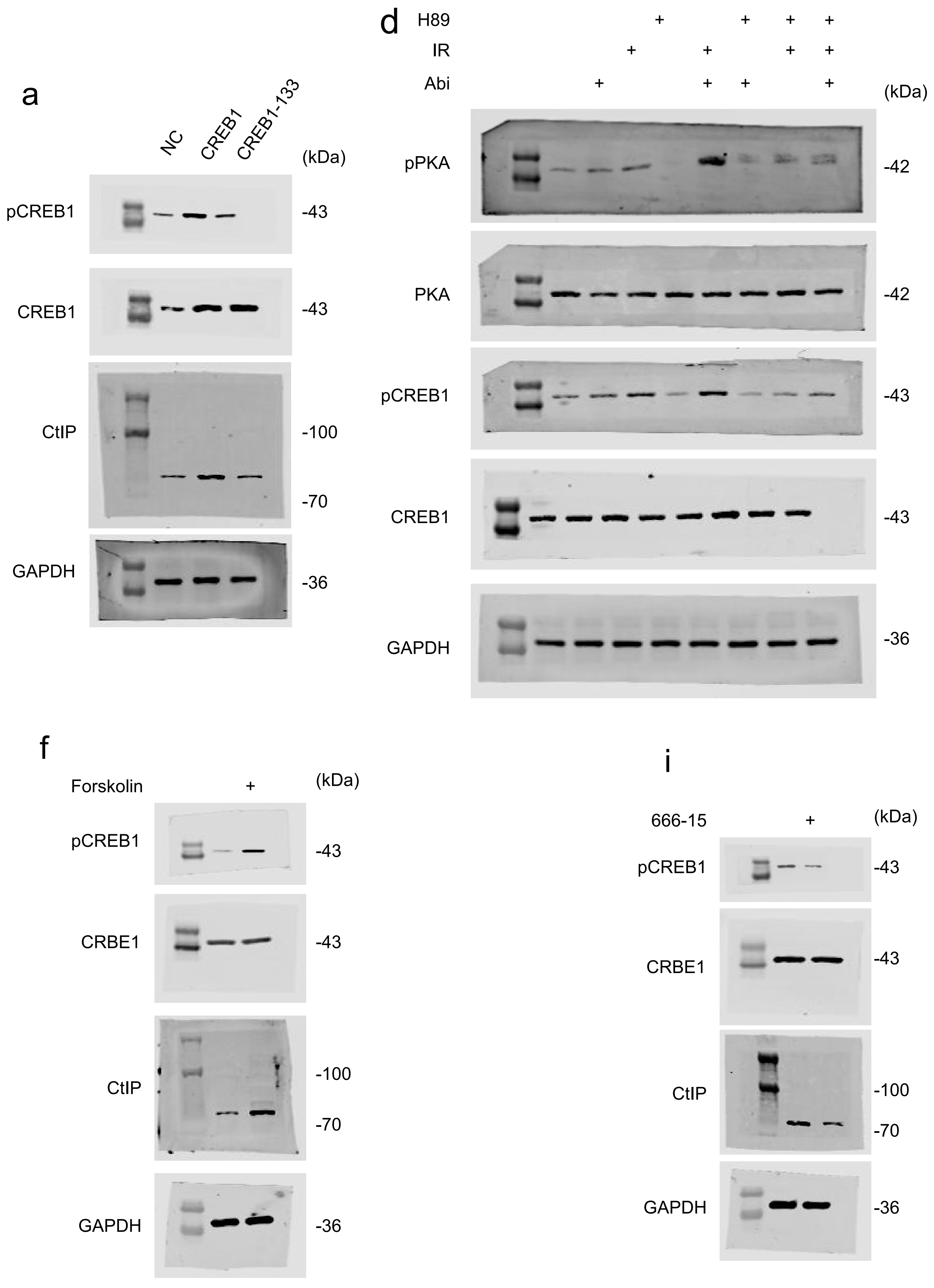

a
NC
CREB1
CREB1-133
(kDa)
pCREB1
-43
CREB1
-43
CtIP
-100
-70
GAPDH
-36
d
H89
IR
Abi
(kDa)
pPKA
-42
PKA
-42
pCREB1
-43
CREB1
-43
GAPDH
-36
f
Forskolin
(kDa)
pCREB1
-43
CRBE1
-43
CtIP
-100
-70
GAPDH
-36
i
666-15
(kDa)
pCREB1
-43
CRBE1
-43
CtIP
-100
-70
GAPDH
-36

Figure 5

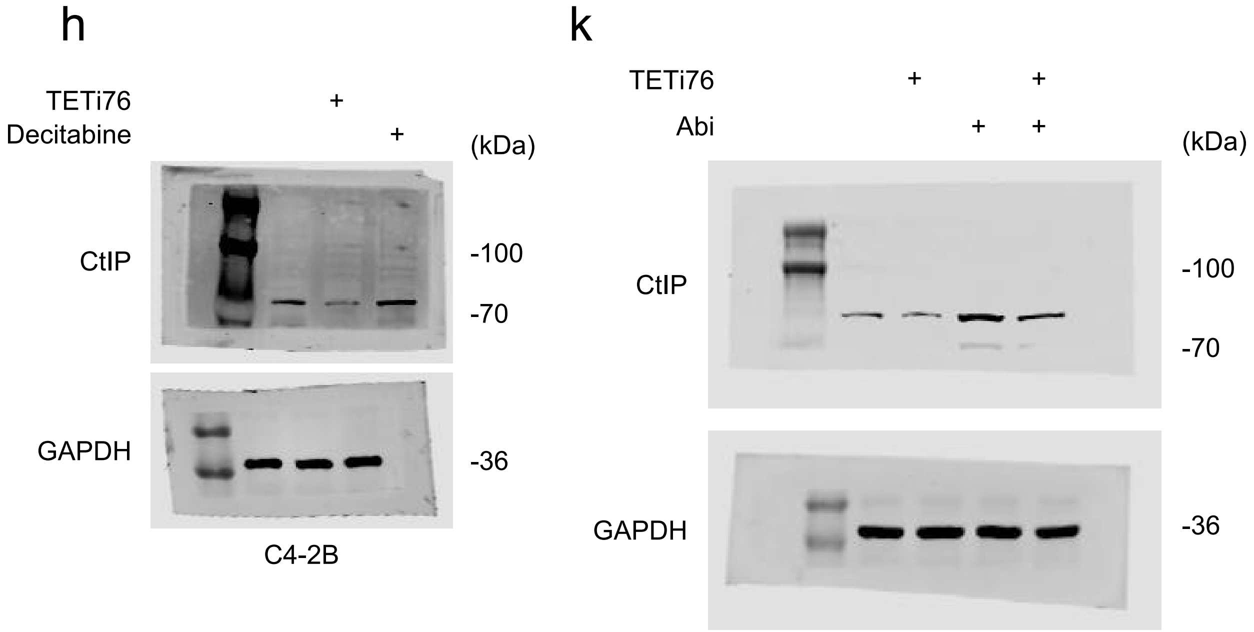

h
TETi76 +
Decitabine +
(kDa)
CtIP
-100
-70
GAPDH
-36
C4-2B
k
TETi76 + +
Abi + +
(kDa)
CtIP
-100
-70
GAPDH
-36

Figure 6

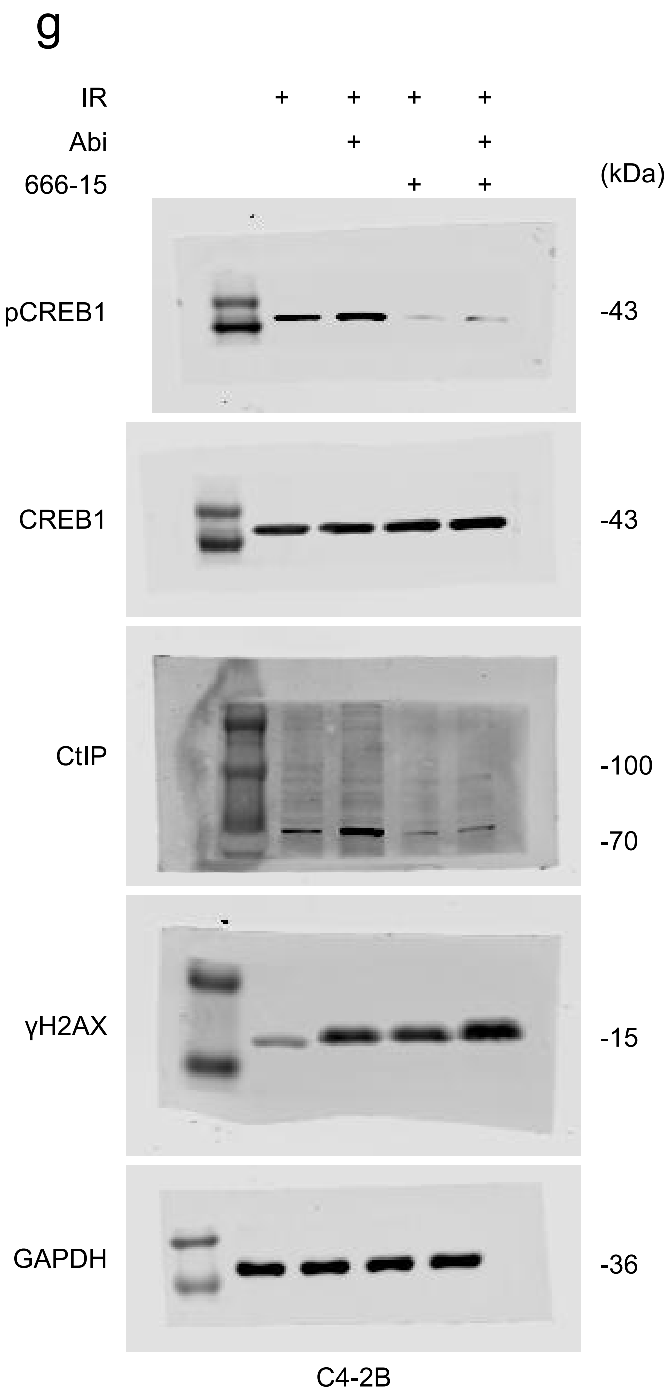
g
IR + + + +
Abi + +
666-15 + +
(kDa)
pCREB1 -43
CREB1 -43
CtIP -100 -70
γH2AX -15
GAPDH -36
C4-2B

Supplementary Figure 1

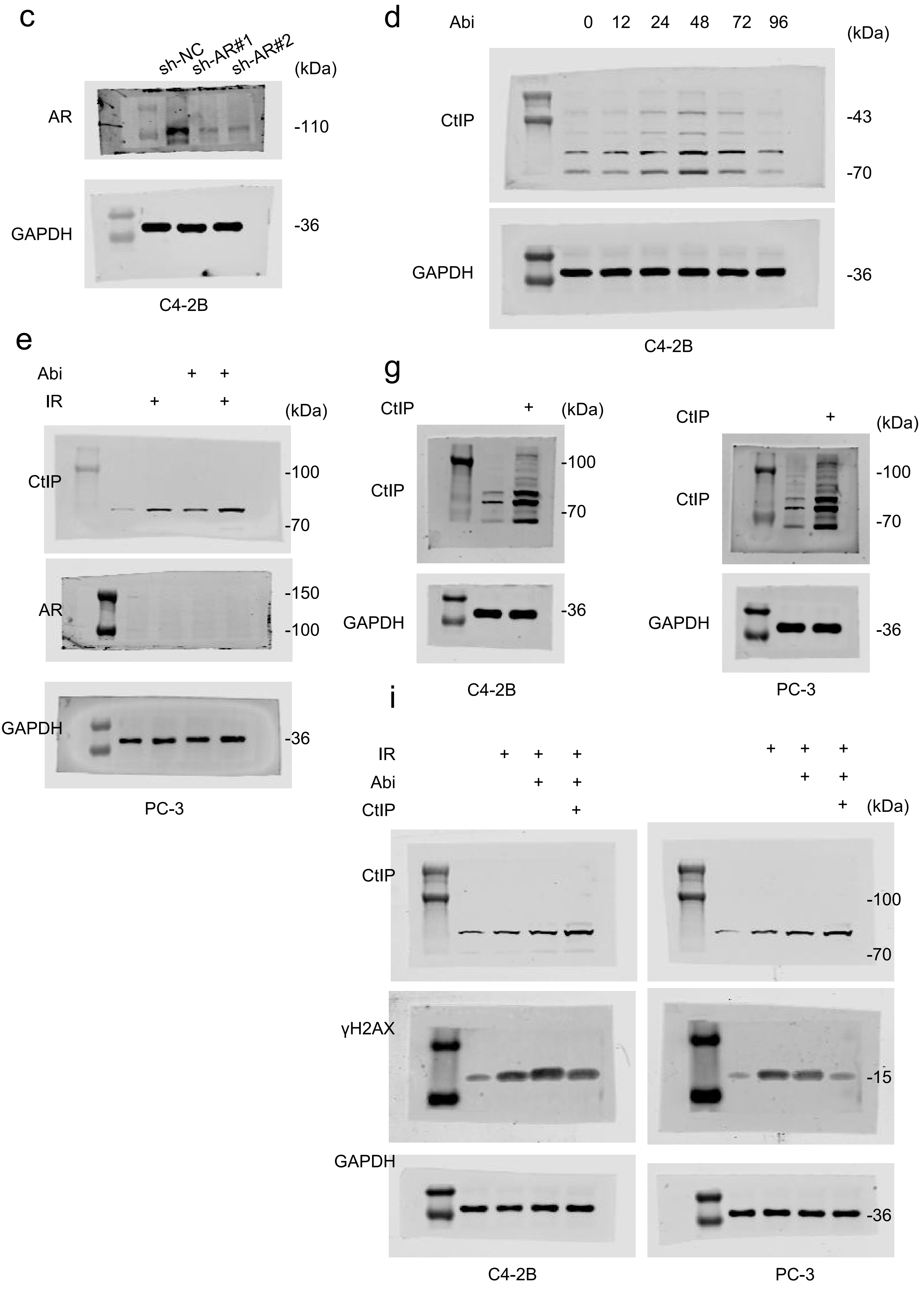

Supplementary Figure 3

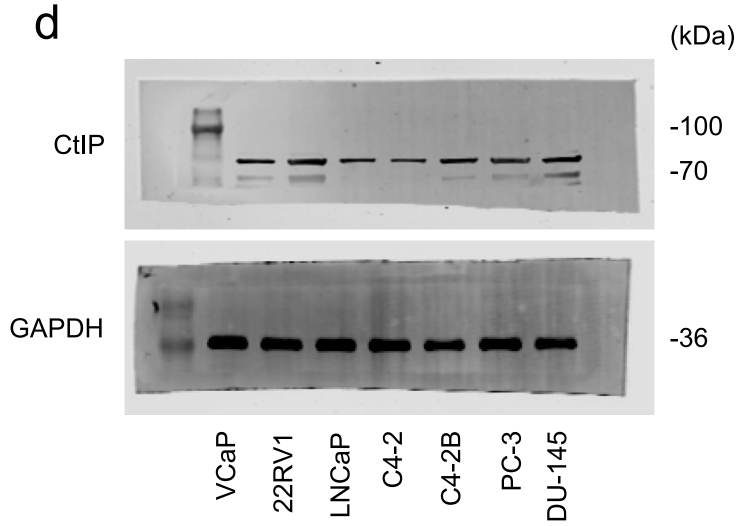

Supplementary Figure 4

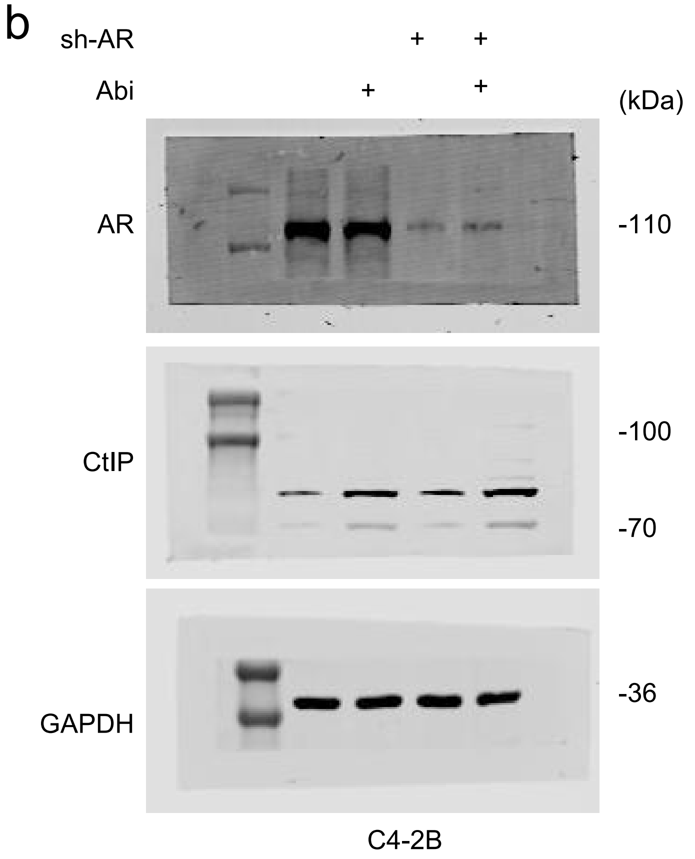

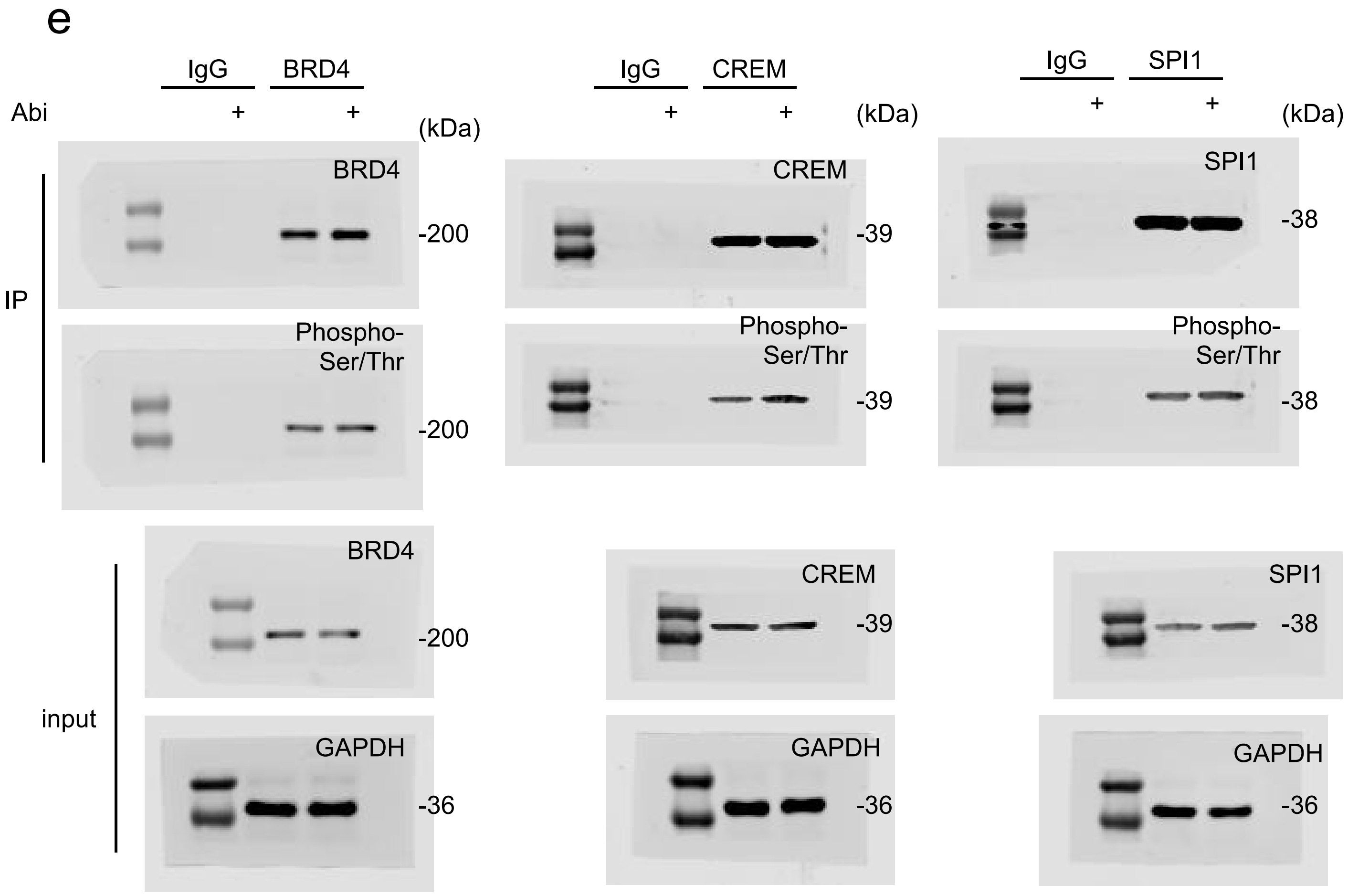

Supplementary Figure 5

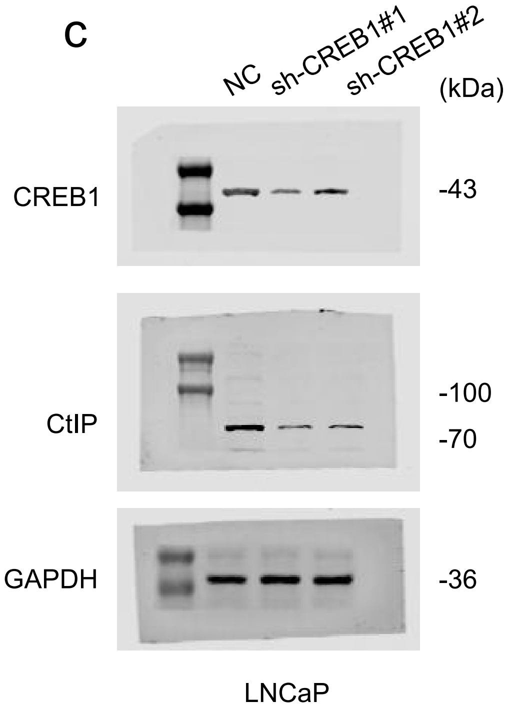

Supplementary Figure 6

a

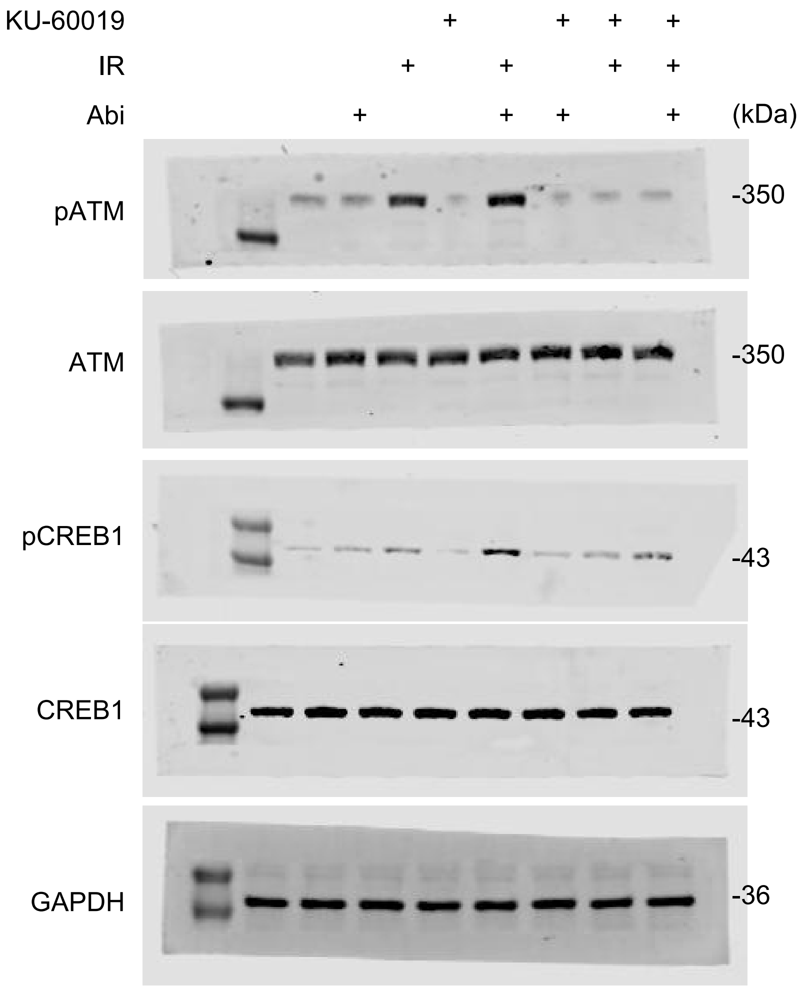

c

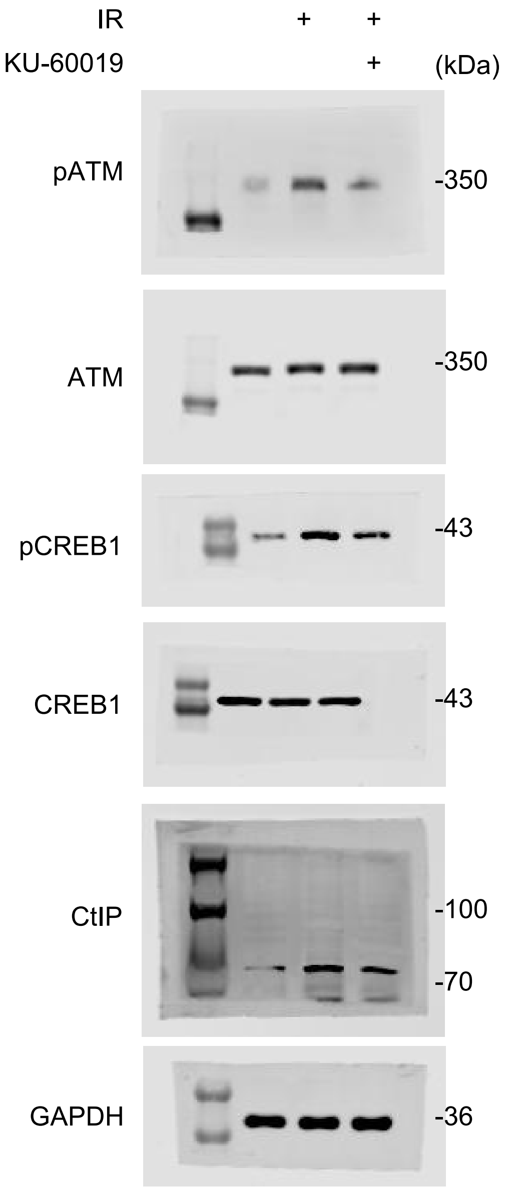

Supplementary Figure 8

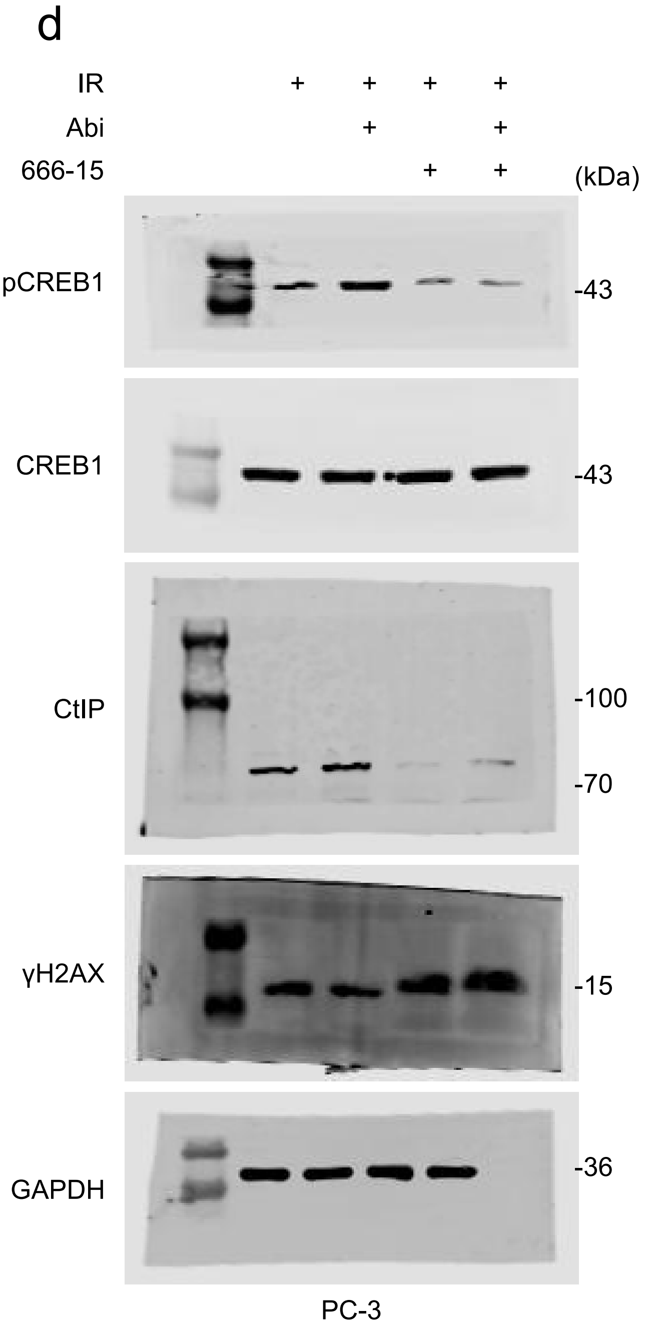

Supplementary Figure 10

a

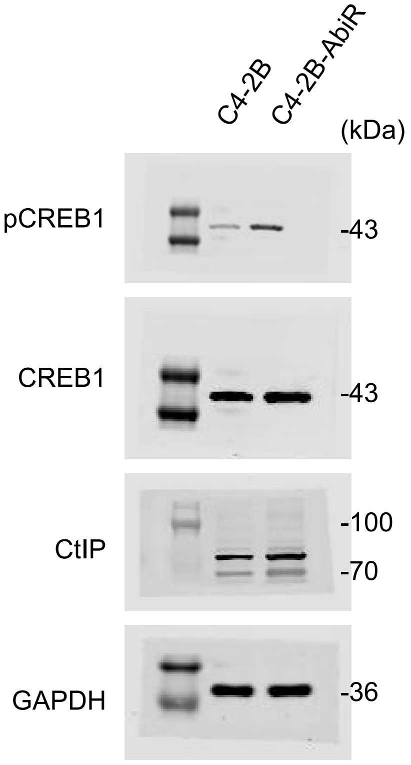

Supplement: Supplementary file 12 — Original western blots [file 41419_2026_8633_MOESM12_ESM.pdf]
